# Synthesis and Biological Activity of Novel (*Z*)- and (*E*)-Verbenone Oxime Esters

**DOI:** 10.3390/molecules22101678

**Published:** 2017-10-12

**Authors:** Qiong Hu, Gui-Shan Lin, Wen-Gui Duan, Min Huang, Fu-Hou Lei

**Affiliations:** 1 School of Chemistry and Chemical Engineering, Guangxi University, Nanning 530004, Guangxi, China; hq460175625@163.com (Q.H.); gaominhuang@mail.gxu.cn (M.H.); 2 Guangxi Key Laboratory of Chemistry and Engineering of Forest Products, Nanning 530008, Guangxi, China; dwg105@gxu.edu.cn

**Keywords:** *α*-pinene, verbenone, oxime, (*Z*)- and (*E*)-isomer, antifungal activity, herbicidal activity

## Abstract

Twenty-seven (*Z*)- and (*E*)-verbenone derivatives bearing an oxime ester moiety were designed and synthesized in search of novel bioactive molecules. Their structures were confirmed by UV-Vis, FTIR, NMR, ESI-MS, and elemental analysis. The antifungal and herbicidal activities of the target compounds were preliminarily evaluated. As a result, compound (*E*)-**4n** (R = *β*-pyridyl) exhibited excellent antifungal activity with growth inhibition percentages of 92.2%, 80.0% and 76.3% against *Alternaria solani*, *Physalospora piricola*, and *Cercospora arachidicola* at 50 µg/mL, showing comparable or better antifungal activity than the commercial fungicide chlorothalonil with growth inhibition of 96.1%, 75.0% and 73.3%, respectively, and 1.7−5.5-fold more growth inhibition than its stereoisomer (*Z*)-**4n** (R = *β*-pyridyl) with inhibition rates of 22.6%, 28.6% and 43.7%, respectively. In addition, seven compounds displayed significant growth inhibition activity of over 90% against the root of rape (*Brassica campestris*) at 100 µg/mL, exhibiting much better herbicidal activity than the commercial herbicide flumioxazin with a 63.0% growth inhibition. Among these seven compounds, compound (*E*)-**4n** (R = *β*-pyridyl) inhibited growth by 92.1%, which was 1.7-fold more than its stereoisomer (*Z*)-**4n** (R = *β*-pyridyl) which inhibited growth by 54.0%.

## 1. Introduction

Verbenone—a natural bicyclic monoterpene containing a ketone group, a carbon-carbon double bond, and a four-membered ring―is found in medicinal plants such as *Verbena triphvlla* and *Eucalyptus globulus* Labill [1]. It can also be conveniently prepared by the regioselective oxidation reaction of *α*-pinene [2], the main component of turpentine oil, which is an abundant natural product. Verbenone was found to have good pesticidal properties such as antiaggregation pheromone activity [3] and pine bark beetle repellent activity [4,5,6], as well as some pharmacological properties like bronchodilating, anti-inflammatory, and haemolytic activities [7]. Also, some verbenone-based amine derivatives were synthesized and found to show insecticidal and antifungal activities [8]. Based on its bioactive properties and chemical reactivity, verbenone deserves further study for pharmaceutical or agrochemical use. On the other hand, oxime ester derivatives were reported to possess diverse biological activities, such as anticancer [9,10], antiviral [11,12], antioxidant [13,14], insecticidal [15,16], antifungal [17,18], and herbicidal [19,20] properties. Although asymmetric oxime esters have (*E*)- and (*Z*)-isomers, the differences between their biological activities rarely receives attention. In fact, taking advantage of the distinction in properties between (*E*)- and (*Z*)-isomers, azobenzene derivatives can perform some amazing functions, for example, to be used as control ion channel molecules [21], molecular devices [22], and photoswitchable antibacterial agents [23], etc., which inspired us to investigate the different biological activities of (*E*)- and (*Z*)-oxime esters with the prospect of potential application. In continuation of our interest in the bioactive properties of natural product-based compounds [24,25,26,27,28,29], a series of novel (*E*)- and (*Z*)-verbenone oxime esters were designed and synthesized by integrating the bioactive oxime ester moiety into the skeleton of verbenone converted from *α*-pinene. Structural characterization, antifungal and herbicidal evaluation of all the title compounds were carried out as well.

## 2. Results and Discussion

### 2.1. Synthesis and Characterization

**Scheme 1:**
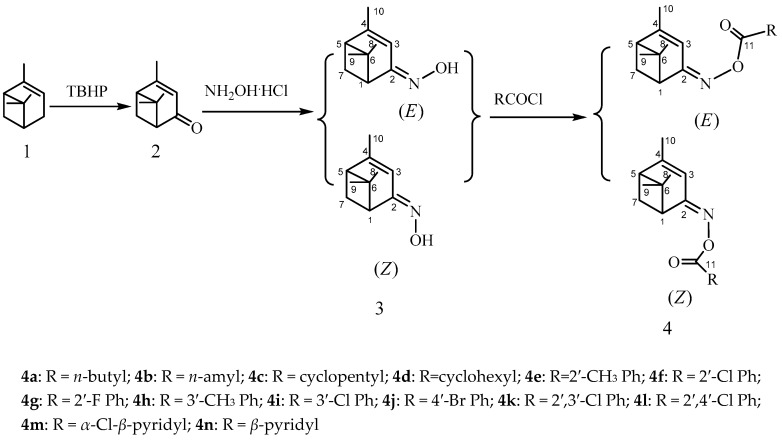
Synthesis of (*Z*)- and (*E*)-verbenone oxime esters **4a**–**4n**.

As illustrated in the Scheme 1, verbenone (**2**) was prepared in 76.2% yield by regioselective oxidation of *α*-pinene using *t*-butyl hydroperoxide (TBHP) as oxidant and CuCl_2_ as catalyst [2], then it underwent condensation with hydroxylamine to give a mixture of (*Z*)- and (*E*)-verbenone oximes (**3**), which were effectively separated in 30.5% and 45.5% yields, respectively, by silica gel column chromatography with step gradient elution with a mixed eluent (petroleum ether:EtOAc = 10:1 to 4:1, *v*/*v*), respectively. Finally, the (*Z*)- and (*E*)-verbenone oxime esters **4a**–**4n** were synthesized by *O*-acylation reactions of the corresponding oximes with acyl chlorides.

**Figure 1:**
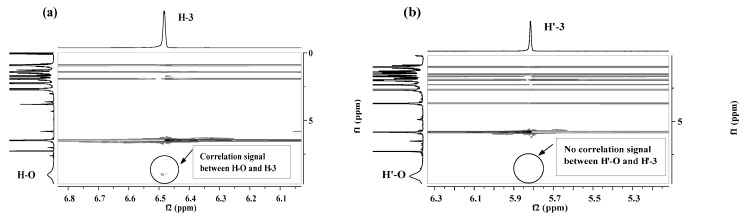
Expanded NOESY spectra of the (*Z*)- and (*E*)-verbenone oximes **3**, (**a**) (*E*)-verbenone oxime; (**b**) (*Z*)-verbenone oxime.

NOESY experiments were employed to identify the (*E*)- and (*Z*)-verbenone oxime isomers **3**, as shown in Figure 1. It was found that there was correlation between the olefinic H-3 proton at 6.48 ppm and the hydroxyl hydrogen H-O (Figure 1a), however, for the other isomer, no correlation signal between the H′-3 olefinic proton at 5.81 ppm and the hydroxyl hydrogen H′-O was found (Figure 1b), implying that H-3 was near to H-O in space, identifying the compound with the H-3 signal at 6.48 ppm as the (*E*)-verbenone oxime (Figure 1a), while the other isomer (H′-3 at 5.81 ppm) was the (*Z*)-verbenone oxime (Figure 1b).

The structures of the target compounds were characterized by IR, ^1^H-NMR, ^13^C-NMR, ESI-MS, and elemental analysis. In the IR spectra, the weak absorption bands at about 3045 cm^−1^ were attributed to the stretching vibrations of the unsaturated C-H in the verbenone moiety. The absorption bands at about 1760 cm^−1^ were due to the vibrations of the carbonyl group C=O.

The weak absorption bands at 1604–1640 cm^−1^ and the strong absorption bands at 1456–1522 cm^−1^ were assigned to the vibrations of C=C in the verbenone moiety and the carbon-nitrogen double bonds C=N, respectively. In the ^1^H-NMR spectra of the *E*-isomers, the olefinic protons of verbenone scaffold showed signals at about 6.00 ppm, but the *Z*-isomers showed at about 6.50 ppm, and the other protons bonded to the saturated carbons of the verbenone moiety displayed signals at about 3.60 ppm in the *E*-form of the target compounds, but at about 3.00 ppm in the *Z*-forms. The ^13^C-NMR spectra of the *E*-form target compounds showed peaks for the olefinic carbons of the verbenone moiety at about 115.0 and 160.0 ppm, but the *Z*-form target compounds showed them at about 110.0 and 163.0 ppm. The carbon-nitrogen double bonds of the *E*-form target compounds appreared at about 168.0 ppm, but the *Z*-form target compounds showed them at about 165.0 ppm. The other saturated carbons displayed signals in the 21.8–49.7 ppm region. Their molecular weights and the C, H, and N element ratios were confirmed by ESI-MS and elemental analysis, respectively.

### 2.2. Antifungal Activity

The antifungal activities of the target compounds (*Z*)- and (*E*)-**4a**–**4n** were evaluated by an in vitro method against fusarium wilt on cucumber (*Fusarium oxysporum f. sp. cucumerinum*), speckle on peanut (*C. arachidicola*), apple root spot (*P. piricola*), tomato early blight (*A. solani*), wheat scab (*Gibberella zeae*), rice sheath blight (*Rhzioeotnia solani*), corn southern leaf blight (*Bipolaris maydis*), and watermelon anthracnose (*Colleterichum orbicalare*) at 50 μg/mL. The results are listed in Table 1.

It was found that, at 50 µg/mL, most of target compounds exhibited certain antifungal activity against the eight tested fungi. On the whole, all the target compounds exhibited the best antifungal activity against *P. piricola*, with an average inhibition activity of 33.5%. Also, compound (*E*)-**4n** (R = *β*-pyridyl) showed the best antifungal activity against all the eight tested fungi, with an average inhibition activity of 63.2%. Compared with that of the commercial fungicide chlorothalonil (positive control), compound (*E*)-**4n** (R = *β*-pyridyl) exhibited excellent antifungal activity with inhibition rates of 92.2%, 80.0%, and 76.3% against *A. solani*, *P. piricola*, and *C. arachidicola*, respectively, displaying better or comparable antifungal activity than that of the positive control with inhibition rates of 96.1%, 75.0%, and 73.3%, respectively. Besides, some compounds displayed moderate activity in the region of 60–80% inhibition rates, although their antifungal activities were inferior to that of the positive control. For example, compound (*E*)-**4h** (R = 3′-CH_3_ Ph) held 70.6%, 65.4%, and 62.6% inhibitory rates against *C. orbiculare*, *C. arachidicola*, and *R. solani*, respectively, as well as compounds (*Z*)-**4j** (R = 4′-Br Ph), (*Z*)-**4i** (R = 3′-Cl Ph), and (Z)-**4m** (R = *α*-Cl-*β*-pyridyl) had inhibition rates of 65.4%, 64.3%, and 62.6% against *P. piricola*, *F. oxysporum*, and *R. solani*, respectively. However, the title compounds showed weak activity against *B. maydis*. To our surprise, some compounds showed large antifungal activity differences between the (*Z*)- and (*E*)-isomers, even against the same fungal species. Particularly, compound (*E*)-**4n** (R = *β*-pyridyl) showed 5.5-, 4.1-, 2.8-, and 1.7-fold higher antifungal activity against *F. oxysporum* f. sp. *cucumerinum*, *R. solani*, *P. piricola*, and *C. arachidicola*, respectively, than its stereoisomer (*Z*)-**4n** (R = *β*-pyridyl).

### 2.3. Herbicidal Activity

The herbicidal activities of the target compounds (*Z*)- and (*E*)-**4a**–**4n** were evaluated by the rape petri dish method and the barnyard grass beaker method against the root-growth of rape (*B. campestris*) and the seedling-growth of barnyard grass (*Echinochloa crusgalli*) at 10 µg/mL and 100 µg/mL, respectively. The results are listed in Table 2.

As shown in Table 2, at 100 µg/mL, the target compounds exhibited remarkable herbicidal activity against the root-growth of rape (*B. campestris*). Among them, seventeen target compounds displayed better herbicidal activity with 63.6–99.3% inhibition rates than that of the commercial herbicidal flumioxazin (positive control) with inhibition rate of 63.0%, in which seven compounds held growth inhibition rates of over 90%. However, the title compounds showed extremely weak inhibition activity against the seedling-growth of barnyard grass (*E. crusgalli*). Interestingly, it was also found that, some compounds showed certain herbicidal activity difference between (*Z*)- and (*E*)-isomers, in which compound (*E*)-**4n** (R = *β*-pyridyl) showed 1.7-fold greater inhibition compared to its stereoisomer (*Z*)-**4n** (R = *β*-pyridyl).

## 3. Materials and Methods

### 3.1. General Information

The GC analysis was performed on an Agilent 6890 GC (Agilent Technologies Inc., Santa Clara, CA, USA) equipped with a HP-1 column (30 m, 0.530 mm, 0.88 μm). IR spectra were recorded on a Nicolet iS50 FT-IR spectrometer (Thermo Scientific Co., Ltd., Madison, WI, USA) using the KBr pellet method. NMR spectra (including ^1^H-NMR, ^13^C-NMR, NOESY) were recorded in CDCl_3_ on an Avance III HD 600 MHz spectrometer (Bruker Co., Ltd., Zurich, Switzerland) and chemical shifts are expressed in ppm (δ) downfield relative to TMS as an internal standard. MS spectra were obtained by means of the electrospray ionization (ESI) method on TSQ Quantum Access MAX HPLC-MS instrument (Thermo Scientific Co., Ltd., Waltham, MA, USA). Elemental analyses were measured using a PE 2400 II elemental analyzer (Perkin-Elmer Instruments Co., Ltd., Waltham, MA, USA). The UV spectra were measured on a UV-1800 spectrophotometer (Shimadzu Corp., Kyoto, Japan). Melting points were determined on a MP420 automatic melting point apparatus (Hanon Instruments Co., Ltd., Jinan, China) and were not corrected. *α*-Pinene (GC purity 96%) was provided by Wuzhou Pine Chemicals Co., Ltd. (Wuzhou, Guangxi, China). Other reagents were purchased from commercial suppliers and used as received.

### 3.2. Synthesis of Verbenone *(**2**)* from α-Pinene

Verbenone (**2**) was prepared as a pale yellow liquid (GC purity 98.9%), according to the literature method [2]. Yield 76.2%. UV-vis (EtOH) λ_max_(log ε): 253.8 (4.26) nm; IR (KBr, cm^−1^): 3040 (=CH), 2975, 2941, 2871 (C-H), 1681 (C=O); ^1^H-NMR (600 MHz, CDCl_3_) δ = 5.72 (s, 1H, H-3), 2.80 (dd, *J* = 8.5, 5.5 Hz, 1H, H-1), 2.66−2.62 (t, *J* = 5.8 Hz, 1H, H-5), 2.41 (t, *J* = 5.8 Hz, 1H, H-7), 2.07 (d, *J* = 9.2 Hz, 1H, H-7), 2.01 (s, 3H, H-10), 1.49 (s, 3H, H-9), 1.01 (s, 3H, H-8); ^13^C-NMR (150 MHz, CDCl_3_) δ = 204.0 (C-2), 170.2 (C-4), 121.2 (C-3), 57.6 (C-1), 53.0 (C-6), 49.7 (C-5), 40.8 (C-7), 26.6 (C-8), 23.6 (C-10), 22.0 (C-9); ESI-MS *m*/*z*: 151.24 ([M + H]^+^).

### 3.3. Synthesis of (Z)- and (E)-verbenone Oximes ***3***

A solution of NH_2_OH·HCl (0.835 g, 12.1 mmol) in H_2_O (5 mL) was added slowly in 1 h to the solution of verbenone (**2**, 1.500 g, 9.99 mmol) in C_2_H_5_OH (10 mL). The reaction mixture was refluxed for 4 h. Then, the reaction mixture was distilled in vacuum to remove solvent, and dichloromethane (10 mL) was added. The mixture was washed with deionized water and purified in silica gel column chromatography by step gradient elution with a mixed eluent (petroleum ether-EtOAc = 25:1, 10:1, *v*/*v*) to give the (Z)- and (*E*)-verbenone oximes.

*(Z)-verbenone oximes* ((*Z*)-**3**). Yield 30.5%. melting point: 105.2–106.9 °C UV-Vis (EtOH) λ_max_(log ε): 258.2 (4.01) nm; IR (KBr, cm^−1^): 3189 (-OH), 3062 (=CH), 2936, 2897 (C-H), 1637, 1470, 1434 (C=N, C=C); ^1^H-NMR (600 MHz, CDCl_3_) δ: 9.20 (s, 1H, H-11), 5.81 (d, *J* = 1.4 Hz, 1H, H-3), 3.65 (td, *J* = 5.9, 1.4 Hz, 1H, H-1), 2.62 (dt, *J* = 8.9, 5.5 Hz, 1H, H-5), 2.26–2.23 (m, 1H, H-7), 1.88 (d, *J* = 1.6 Hz, 3H, H-10), 1.63 (d, *J* = 8.9 Hz, 1H, H-7), 1.47 (s, 3H, H-9), 0.91 (s, 3H, H-8); ^13^C-NMR (150 MHz, CDCl_3_) δ = (150 MHz, CDCl_3_) 159.7 (C-2), 158.7 (C-4), 109.6 (C-3), 49.4 (C-1), 48.3 (C-6), 48.0 (C-5), 37.5 (C-7), 26.1 (C-8), 23.6 (C-10), 21.9 (C-9); MS (ESI) *m*/*z*: 165.93 ([M + H]^+^).

*(E)-verbenone oxime* ((*E*)-**3**). Yield 45.5%. melting point: 132.7–135.9 °C UV-Vis (EtOH) λ_max_(log ε): 247.0 (4.08) nm; IR (KBr, cm^−1^): 3189 (-OH), 3059 (=CH), 2968, 2900 (C-H), 1637, 1480, 1442 (C=N, C=C); ^1^H-NMR (600 MHz, CDCl_3_) δ: 9.05 (s, 1H, H-11), 6.48 (dd, *J* = 3.0, 1.5 Hz, 1H, H-3), 2.71 (td, *J* = 6.0, 1.5 Hz, 1H, H-1), 2.66 (dt, *J* = 8.9, 5.5 Hz, 1H, H-5), 2.26 (dd, *J* = 8.4, 3.2 Hz, 1H, H-7), 1.93 (d, *J* = 1.5 Hz, 3H, H-10), 1.73 (d, *J* = 8.9 Hz, 1H, H-7), 1.43 (s, 3H, H-9), 0.92 (s, 3H, H-8); ^13^C-NMR (150 MHz, CDCl_3_) δ = 162.1 (C-2), 154.3 (C-4), 115.6 (C-3), 49.1 (C-1), 47.3 (C-6), 41.5 (C-5), 36.3 (C-7), 26.1 (C-8), 23.1 (C-10), 22.2 (C-9); ESI-MS *m*/*z*: 165.94. ([M + H]^+^).

### 3.4. General Procedure for the Synthesis of (Z)- and (E)-verbenone Oxime Esters ***4***

Under an anhydrous atmosphere, acyl chloride (1.1 mmol) was added slowly to a stirred solution of (*Z*)- or (*E*)-verbenone oxime (**3**, 0.17 g, 1.00 mmol) in dichloromethane (5 mL) and ten drops of triethylamine in an ice-water bath. The reaction process was monitored by TLC. Upon completion, 5 mL deionized water was added to destroy the unreacted acyl chloride. Then, the organic layer was separated, washed with deionized water three times, and concentrated in vacuum. The crude product was further purified by silica gel chromatography to afford the target compounds (*Z*)- and (*E*)-**4a**–**4n**.

*(Z)-verbenone O-n-pentanoyl oxime* ((*Z*)-**4a**). Light yellow liquid. Yield: 77.0%, melting point: 69.3–70.3 °C. UV-Vis (EtOH) λ_max_(log ε): 255.4 (4.33) nm; IR (KBr, cm^−1^): 3084 (=C-H), 2958, 2930, 2872 (C-H), 1759 (C=O),1622, 1603 (C=N, C=C); ^1^H-NMR (600 MHz, CDCl_3_): δ = 6.46–6.23 (m, 1H, H-3), 2.93 (t, *J* = 5.9 Hz, 1H, H-1, 2.74–2.70 (m, 1H, H-5), 2.43 (t, *J* = 7.6 Hz, 2H, H-12), 2.31 (t, *J* = 5.7 Hz, 1H, H-7a), 1.97 (dd, *J* = 1.5, 0.7 Hz, 3H, H-10), 1.80 (d, *J* = 9.2 Hz, 1H, H-7b), 1.73–1.69 (m, 2H, H-13), 1.45 (s, 3H, H-9), 1.42–1.39 (m, 2H, H-14), 0.95–0.92 (m, 6H, H-8,15); ^13^C-NMR (150 MHz, CDCl_3_): δ = 171.5 (C-11), 165.8 (C-2), 163.4 (C-4), 110.0 (C-3), 49.7 (C-1), 49.4 (C-6), 48.3 (C-5), 38.4 (C-12), 32.8 (C-7), 27.1 (C-13), 26.1 (C-8), 23.8 (C-14), 22.3 (C-10), 21.8 (C-9), 13.7 (C-15); ESI-MS *m*/*z*: 249.91 [M + H]^+^. Anal. calcd. For C_15_H_23_NO_2_: C, 72.25; H, 9.30; N, 5.62; Found: C, 72.22; H, 9.21; N, 5.59.

*(E)-verbenone O-n-pentanoyl oxime* ((*E*)-**4a**) as a yellow liquid, Yield 86.0% melting point: 78.8–81.3 °C. UV-Vis (EtOH) λ_max_(log ε): 252.1 (4.37) nm; IR (KBr, cm^−1^): 3053 (=C-H), 2958, 2933, 2872 (C-H), 1760 (C=O),1628, 1595 (C=N, C=C); ^1^H-NMR (600 MHz, CDCl_3_): δ = 6.00–5.97 (m, 1H, H-3), 3.52 (td, *J* = 5.8, 1.6 Hz, 1H, H-1), 2.66–2.63 (m, 1H, H-5), 2.42–2.39 (m, 2H, H-12), 2.30–2.27 (m, 1H, H-7a), 1.93 (d, *J* = 1.5 Hz, 3H, H-10), 1.73 (d, *J* = 9.1 Hz, 1H, H-7b), 1.69–1.66 (m, 2H, H-13), 1.48 (s, 3H, H-9), 1.41–1.38 (m, 2H, H-14), 0.92 (t, *J* = 3.7 Hz, 6H, H-8,15); ^13^C-NMR (150 MHz, CDCl_3_): δ = 171.7 (C-11), 168.1 (C-2), 159.2 (C-4), 115.1 (C-3), 49.2 (C-1), 49.1 (C-6), 43.7 (C-5), 37.2 (C-12), 32.8 (C-7), 27.0 (C-13), 26.2 (C-8), 23.4 (C-14), 22.3 (C-10), 22.2 (C-9), 13.7 (C-15); ESI-MS *m*/*z*: 249.84 [M + H]^+^. Anal. calcd. For C_15_H_23_NO_2_: C, 72.25; H, 9.30; N, 5.62; Found: C, 72.20; H, 9.23; N, 5.60.

*(Z)-verbenone O-n-hexanoyl oxime* ((*Z*)-**4b**). Yellow liquid. Yield: 80.0%, melting point: 62.1–63.2 °C. UV-Vis (EtOH) λ_max_(log ε): 254.0 (4.38) nm; IR (KBr, cm^−1^): 3050 (=C-H), 2958, 2933, 2872 (C-H), 1760 (C=O),1628, 1600 (C=N, C=C); ^1^H-NMR (600 MHz, CDCl_3_): δ = 6.37–6.33 (m, 1H, H-3), 2.93 (t, *J* = 5.9 Hz, 1H, H-1), 2.72 (dt, *J* = 10.9, 5.5 Hz, 1H, H-5), 2.42 (t, *J* = 7.6 Hz, 2H, H-12), 2.31 (t, *J* = 5.7 Hz, 1H, H-7a), 1.96 (d, *J* = 1.6 Hz, 3H, H-10), 1.80 (d, *J* = 9.2 Hz, 1H, H-7b), 1.74–1.69 (m, 2H, H-13), 1.45 (s, 3H, H-9), 1.37–1.34 (m, 4H, H-14,15), 0.94 (s, 3H, H-8), 0.91 (t, *J* = 6.2 Hz, 3H, H-16); ^13^C-NMR (150 MHz, CDCl_3_): δ = 171.5 (C-11), 165.8 (C-2), 163.4 (C-4), 110.0 (C-3), 49.7 (C-1), 49.4 (C-6), 48.3 (C-5), 38.4 (C-12), 33.1 (C-14), 31.4 (C-7), 26.1 (C-8), 24.7 (C-13), 23.8 (C-15), 22.3 (C-10), 21.8 (C-9), 13.9 (C-16); ESI-MS *m*/*z*: 263.80 [M + H]^+^. Anal. calcd. For C_16_H_25_NO_2_: C, 72.97; H, 9.57; N, 5.32; Found: C, 72.65; H, 9.47; N, 5.29.

*(E)-verbenone O-n-hexanoyl oxime* ((*E*)-**4b**). Light yellow liquid. Yield: 72.0%, melting point: 78.2–79.6 °C. UV-Vis (EtOH) λ_max_(log ε): 252.5 (4.33) nm; IR (KBr, cm^−1^): 3053 (=C-H), 2957, 2933, 2872 (C-H), 1760 (C=O), 1629, 1600 (C=N, C=C); ^1^H-NMR (600 MHz, CDCl_3_): δ = 5.99 (d, *J* = 1.7 Hz, 1H, H-3), 3.52 (td, *J* = 5.8, 1.6 Hz, 1H, H-1), 2.65 (dt, *J* = 9.1, 5.5 Hz, 1H, H-5), 2.40 (t, *J* = 7.6 Hz, 2H, H-12), 2.30–2.27 (m, 1H, H-7a), 1.93 (d, *J* = 1.6 Hz, 3H, H-10), 1.73 (d, *J* = 9.1 Hz, 1H, H-7b), 1.71–1.66 (m, 2H, H-13), 1.48 (s, 3H, H-9), 1.34 (q, *J* = 3.6 Hz, 4H, H-14,15), 0.92 (s, 3H, H-8), 0.91–0.89 (m, 3H, H-16); ^13^C-NMR (150 MHz, CDCl_3_): δ = 171.7 (C-11), 168.1 (C-2), 159.2 (C-4), 115.1 (C-3), 49.2 (C-1), 49.1 (C-6), 43.7 (C-5), 37.2 (C-12), 33.1 (C-14), 31.3 (C-7), 26.2 (C-8), 24.7 (C-13), 23.4 (C-15), 22.3 (C-10), 22.2 (C-9), 13.9 (C-16); ESI-MS *m*/*z*: 263.88 [M + H]^+^. Anal. calcd. For C_16_H_25_NO_2_: C, 72.97; H, 9.57; N, 5.32; Found: C, 72.71; H, 9.49; N, 5.30.

*(Z)-verbenone O-cyclopentylcarbonyl oxime* ((*Z*)-**4c**). Slight brown liquid. Yield: 90.0%, melting point: 90.2–91.3 °C. UV-Vis (EtOH) λ_max_(log ε): 256.9 (4.28) nm; IR (KBr, cm^−1^): 3082, 3061 (Ar-H, =C-H), 2961, 2899, 2876 (C-H), 1755 (C=O), 1621, 1599, 1577 (C=N, Ar-C=C, C=C); ^1^H-NMR (600 MHz, CDCl_3_): δ = 6.35 (d, *J* = 1.5 Hz, 1H, H-3), 2.94 (td, *J* = 6.0, 1.6 Hz, 1H, H-12), 2.85 (p, *J* = 8.1 Hz, 1H, H-1), 2.72 (dt, *J* = 9.2, 5.5 Hz, 1H, H-5), 2.33–2.29 (m, 1H, H-7a), 1.97 (d, *J* = 1.6 Hz, 3H, H-10), 1.94 (d, *J* = 5.5 Hz, 2H, H-16), 1.93–1.85 (m, 2H, H-13), 1.80 (d, *J* = 9.2 Hz, 1H, H-7b), 1.78–1.72 (m, 2H, H-15), 1.61 (dt, *J* = 9.0, 4.2 Hz, 2H, H-9), 1.45 (s, 3H, H-14), 0.94 (s, 3H, H-8); ^13^C-NMR (150 MHz, CDCl_3_): δ = 174.3 (C-11), 165.8 (C-2), 163.3 (C-4), 110.0 (C-3), 49.7 (C-1), 49.4 (C-6), 48.3 (C-5), 42.8 (C-12), 38.4 (C-16), 30.2 (C-13), 30.1 (C-7), 26.1 (C-15), 25.9 (C-14), 25.9 (C-8), 23.9 (C-10), 21.8 (C-9); ESI-MS *m*/*z*: 261.85 [M + H]^+^. Anal. calcd. For C_16_H_23_NO_2_: C, 73.53; H, 8.87; N, 5.36; Found: C, 73.25; H, 8.78; N, 5.33.

*(E)-verbenone O-cyclopentylcarbonyl oxime* ((*E*)-**4c**). Brown liquid. Yield: 90.0%, melting point: 102.11–104.35 °C. UV-Vis (EtOH) λ_max_(log ε): 252.6 (4.25) nm; IR (KBr, cm^−1^): 3066 (= C-H), 2959, 2933, 2873 (C-H), 1741 (C=O), 1619, 1436 (C=N, C=C); ^1^H-NMR (600 MHz, CDCl_3_): δ = 5.99 (q, *J* = 1.6 Hz, 1H, H-3), 3.51 (td, *J* = 5.8, 1.7 Hz, 1H, H-12), 2.82 (p, *J* = 8.1 Hz, 1H, H-1), 2.64 (dt, *J* = 9.1, 5.5 Hz, 1H, H-5), 2.28 (td, *J* = 5.9, 1.4 Hz, 1H, H-7a), 1.92 (d, *J* = 1.6 Hz, 3H, H-10), 1.90–1.85 (m, 2H, H-16), 1.78–1.74 (m, 1H, H-7b), 1.74–1.72 (m, 2H, H-13), 1.60 (dd, *J* = 7.2, 4.0 Hz, 2H, H-15), 1.48 (s, 3H, H-9), 1.46–1.24 (m, 2H, H-14), 0.92 (s, 3H, H-8); ^13^C-NMR (150 MHz, CDCl_3_): δ = 174.5 (C-11), 168.1 (C-2), 159.1 (C-4), 115.2 (C-3), 49.2 (C-1), 49.1 (C-6), 43.7 (C-5), 42.7 (C-12), 37.2 (C-16), 30.2 (C-13), 30.1 (C-7), 26.2 (C-15), 25.8 (C-14), 25.8 (C-8), 23.4 (C-10), 22.3 (C-9); ESI-MS *m*/*z*: 261.86 [M + H]^+^. Anal. calcd. For C_16_H_23_NO_2_: C, 73.53; H, 8.87; N, 5.36; Found: C, 73.29; H, 8.80; N, 5.34.

*(Z)-verbenone O-cyclohexylcarbonyl oxime* ((*Z*)-**4d**). Yellow liquid. Yield: 93.0%, melting point: 95.7–98.2 °C. UV-Vis (EtOH) λ_max_(log ε): 256.5 (4.57) nm; IR (KBr, cm^−1^): 3071 (=C-H), 2980, 2933, 2856 (C-H), 1756 (C=O), 1621, 1600 (C=N, C=C); ^1^H-NMR (600 MHz, CDCl_3_): δ = 6.34 (q, *J* = 1.6 Hz, 1H, H-3), 2.94 (td, *J* = 5.9, 1.7 Hz, 1H, H-12), 2.71 (dt, *J* = 9.2, 5.5 Hz, 1H, H-1), 2.45 (ddt, *J* = 11.5, 7.8, 3.6 Hz, 1H, H-5), 2.32–2.29 (m, 1H, H-7a), 2.01–1.98 (m, 2H, H-17), 1.97 (d, *J* = 1.6 Hz, 3H, H-10), 1.80 (d, *J* = 9.2 Hz, 2H, H-13), 1.67 (d, *J* = 10.9 Hz, 1H, H-7b), 1.60–1.54 (m, 2H, H-15), 1.45 (s, 3H, H-9), 1.34–1.25 (m, 4H, H-14,16), 0.94 (s, 3H, H-8); ^13^C-NMR (150 MHz, CDCl_3_): δ = 173.4 (C-11), 165.9 (C-2), 163.3 (C-4), 110.0 (C-3), 49.7 (C-1), 49.4 (C-6), 48.3 (C-5), 42.5 (C-12), 38.4 (C-17), 29.2 (C-13), 29.1 (C-7), 26.1 (C-15), 25.7 (C-16), 25.5 (C-14), 25.5 (C-8), 23.8 (C-10), 21.8 (C-9); ESI-MS *m*/*z*: 275.86 [M + H]^+^. Anal. calcd. For C_17_H_25_NO_2_: C, 74.14; H, 9.15; N, 5.09; Found: C, 73.85; H, 9.06; N, 5.06.

*(E)-verbenone O-cyclohexylcarbonyl oxime* ((*E*)-**4d**). Yellow liquid. Yield: 95.0%, melting point: 104.3–105.4 °C. UV-Vis (EtOH) λ_max_(log ε): 251.9 (4.33) nm; IR (KBr, cm^−1^): 3048 (=C-H), 2985, 2933, 2856 (C-H), 1759 (C=O), 1630, 1598 (C=N, C=C); ^1^H-NMR (600 MHz, CDCl_3_): δ = 5.99 (q, *J* = 1.6 Hz, 1H, H-3), 3.51 (td, *J* = 5.8, 1.7 Hz, 1H, H-12), 2.64 (dt, *J* = 9.1, 5.5 Hz, 1H, H-1), 2.41 (tt, *J* = 11.4, 3.6 Hz, 1H, H-5), 2.28 (td, *J* = 5.7, 1.5 Hz, 1H, H-7a), 1.97 (d, *J* = 1.6 Hz, 2H, H-17), 1.92 (d, *J* = 1.6 Hz, 3H, H-10), 1.79–1.76 (m, 2H, H-13), 1.67–1.64 (m, 1H, H-7b), 1.56–1.50 (m, 2H, H-15), 1.48 (s, 3H, H-9), 1.32–1.23 (m, 4H, H-14,16), 0.92 (s, 3H, H-8); ^13^C-NMR (150 MHz, CDCl_3_): δ = 173.6 (C-11), 168.2 (C-2), 159.1 (C-4), 115.2 (C-3), 49.2 (C-1), 49.1 (C-6), 43.7 (C-5), 42.4 (C-12), 37.2 (C-17), 29.1 (C-13), 29.0 (C-7), 26.3 (C-15), 25.7 (C-16), 25.5 (C-14), 25.5 (C-8), 23.4 (C-10), 22.3 (C-9); ESI-MS *m*/*z*: 275.94 [M + H]^+^. Anal. calcd. For C_17_H_25_NO_2_: C, 74.14; H, 9.15; N, 5.09; Found: C, 73.89; H, 9.08; N, 5.07.

*(Z)-verbenone O-(2′-methylbenzoyl) oxime* ((*Z*)-**4e**). White solid. Yield: 95.0%, melting point: 86.3–87.4 °C. UV-Vis (EtOH) λ_max_(log ε): 262.9 (4.42), 237.8 (4.28) nm; IR (KBr, cm^−1^): 3027 (Ar-H), 2962, 2943, 2897 (C-H), 1740 (C=O), 1623, 1600, 1575 (C=N, Ar-C=C, C=C); ^1^H-NMR (600 MHz, CDCl_3_): δ = 7.90 (dd, *J* = 8.1, 1.2 Hz, 1H, H-17), 7.42 (td, *J* = 7.5, 1.3 Hz, 1H, H-15), 7.29–7.26 (m, 2H, H-16,14), 6.43 (d, *J* = 1.5 Hz, 1H, H-3), 3.03 (td, *J* = 6.0, 1.5 Hz, 1H, H-5), 2.76 (dt, *J* = 9.3, 5.5 Hz, 1H, H-1), 2.64 (s, 3H, H-18), 2.35–2.32 (m, 1H, H-7a), 1.98 (d, *J* = 1.5 Hz, 3H, H-10), 1.85 (d, *J* = 9.2 Hz, 1H, H-7b), 1.48 (s, 3H, H-9), 0.98 (s, 3H, H-8); ^13^C-NMR (150 MHz, CDCl_3_): δ = 166.3 (C-11), 165.5 (C-2), 163.7 (C-4), 134.0 (C-13), 131.8 (C-15), 131.6 (C-12), 130.1 (C-14), 129.3 (C-17), 125.7 (C-16), 110.3 (C-3), 49.8 (C-1), 49.5 (C-6), 48.4 (C-5), 38.5 (C-7), 26.2 (C-8), 23.9 (C-10), 21.9 (C-9), 21.4 (C-18); ESI-MS *m*/*z*: 283.84 [M + H]^+^. Anal. calcd. For C_18_H_21_NO_2_: C, 76.30; H, 7.47; N, 4.94; Found: C, 75.98; H, 7.40; N, 4.92.

*(E)-verbenone O-(2′-methylbenzoyl) oxime* ((*E*)-**4e**). White solid. Yield: 90.0%, melting point: 87.8–90.4 °C. UV-Vis (EtOH) λ_max_(log ε): 262.7 (4.33), 238.0 (4.19) nm; IR (KBr, cm^−1^): 3027 (Ar-H), 2962, 2943, 2868 (C-H), 1740 (C=O), 1623, 1560, 1574 (C=N, Ar-C=C, C=C); ^1^H-NMR (600 MHz, CDCl_3_): δ = 7.90 (dd, *J* = 8.0, 1.5 Hz, 1H, H-17), 7.42 (td, *J* = 7.5, 1.5 Hz, 1H, H-15), 7.31–7.26 (m, 2H, H-16,14), 6.43 (d, *J* = 1.5 Hz, 1H, H-3), 3.03 (td, *J* = 5.9, 1.7 Hz, 1H, H-5), 2.78–2.74 (m, 1H, H-1), 2.64 (s, 3H, H-18), 2.33 (ddd, *J* = 6.5, 5.3, 1.4 Hz, 1H, H-7a), 1.98 (d, *J* = 1.7 Hz, 3H, H-10), 1.85 (d, *J* = 9.3 Hz, 1H, H-7b), 1.48 (s, 3H, H-9), 0.98 (s, 3H, H-8); ^13^C-NMR (150 MHz, CDCl_3_): δ = 166.3 (C-11), 165.5 (C-2), 163.7 (C-4), 134.0 (C-13), 131.8 (C-15), 131.6 (C-12), 130.1 (C-14), 129.3 (C-17), 125.7 (C-16), 110.3 (C-3), 49.8 (C-1), 49.5 (C-6), 48.4 (C-5), 38.5 (C-7), 26.2 (C-8), 23.9 (C-10), 21.9 (C-9), 21.4 (C-18); ESI-MS *m*/*z*: 283.82 [M + H]^+^. Anal. calcd. For C_18_H_21_NO_2_: C, 76.30; H, 7.47; N, 4.94; Found: C, 76.05; H, 7.41; N, 4.93.

*(Z)-verbenone-based O-(2′-chlorobenzoyl) oxime* ((*Z*)-**4f**). Yellow solid. Yield: 91.2%, melting point: 101.1–104.0 °C. UV-Vis (EtOH) λ_max_(log ε): 263.1 (3.92), 202.5 (4.36) nm; IR (KBr, cm^−1^): 3083, 3060 (Ar-H, =C-H), 2975, 2955, 2867 (C-H), 1736 (C=O), 1620, 1591, 1469 (C=N, Ar-C=C, C=C); ^1^H-NMR (600 MHz, CDCl_3_): δ = 7.85 (dd, *J* = 7.7, 1.6 Hz, 1H, H-17), 7.48 (dd, *J* = 8.0, 1.1 Hz, 1H, H-14), 7.44 (td, *J* = 7.7, 1.7 Hz, 1H, H-15), 7.35 (td, *J* = 7.6, 1.3 Hz, 1H, H-16), 6.48 (d, *J* = 1.7 Hz, 1H, H-3), 3.02 (td, *J* = 6.0, 1.6 Hz, 1H, H-1), 2.76 (dt, *J* = 9.3, 5.5 Hz, 1H, H-5), 2.36 – 2.31 (m, 1H, H-7a), 1.97 (d, *J* = 1.6 Hz, 3H, H-10), 1.86 (d, *J* = 9.2 Hz, 1H, H-7b), 1.48 (s, 3H, H-9), 0.98 (s, 3H, H-8); ^13^C-NMR (150 MHz, CDCl_3_): δ = 167.0 (C-11), 164.2 (C-2), 164.0 (C-4), 133.3 (C-15), 132.5 (C-13), 131.5 (C-12), 130.9 (C-17), 130.2 (C-14), 126.7 (C-16), 110.5 (C-3), 50.1 (C-1), 49.5 (C-6), 48.3 (C-5), 38.6 (C-7), 26.2 (C-8), 23.9 (C-10), 21.8 (C-9); ESI-MS *m*/*z*: 303.72 [M + H]^+^. Anal. calcd. For C_17_H_18_ClNO_2_: C, 66.95; H, 5.97; N, 4.61; Found: C, 67.21; H, 5.91; N, 4.59.

*(E)-verbenone O-(2′-chlorobenzoyl) oxime* ((*E*)-**4f**). Yellow solid. Yield: 91.0%, melting point: 104.4–107.2 °C. UV-Vis (EtOH) λ_max_(log ε): 262.3 (4.01), 203.2 (4.35) nm; IR (KBr, cm^−1^): 3081, 3060 (Ar-H, =C-H), 2979, 2952, 2865 (C-H), 1747 (C=O),1621, 1591, 1466 (C=N, Ar-C=C, C=C); ^1^H-NMR (600 MHz, CDCl_3_): δ = 7.80 (dd, *J* = 7.7, 1.6 Hz, 1H, H-17), 7.47 (d, *J* = 1.5 Hz, 1H, H-14), 7.45–7.41 (m, 1H, H-15), 7.35–7.33 (m, 1H, H-16), 6.07 (d, *J* = 1.6 Hz, 1H, H-3), 3.67 (td, *J* = 5.8, 1.8 Hz, 1H, H-1), 2.68–2.64 (m, 1H, H-5), 2.31 (td, *J* = 5.7, 1.5 Hz, 1H, H-7a), 1.96 (d, *J* = 1.7 Hz, 3H, H-10), 1.78 (d, *J* = 9.2 Hz, 1H, H-7b), 1.48 (s, 3H, H-9), 0.96 (s, 3H, H-8); ^13^C-NMR (150 MHz, CDCl_3_): δ = 169.3 (C-11), 164.1 (C-2), 160.1 (C-4), 133.2 (C-15), 132.5 (C-13), 131.5 (C-12), 130.9 (C-17), 130.1 (C-14), 126.7 (C-16), 114.8 (C-3), 49.7 (C-1), 49.1 (C-6), 44.4 (C-5), 37.4 (C-7), 26.2 (C-8), 23.5 (C-10), 22.3 (C-9); ESI-MS *m*/*z*: 303.72 [M + H]^+^. Anal. calcd. For C_17_H_18_ClNO_2_: C, 67.21; H, 5.97; N, 4.61; Found: C, 66.97; H, 5.93; N, 4.60.

*(Z)-verbenone O-(2′-fluorobenzoyl) oxime* ((*Z*)-**4g**). Yellow solid. Yield: 89.8%, melting point: 68.9–71.9 °C. UV-Vis (EtOH) λ_max_(log ε): 265.1 (4.28), 226.6 (4.26) nm; IR (KBr, cm^−1^): 3064, 3041 (Ar-H, =C-H), 3000, 2964, 2865 (C-H), 1731 (C=O), 1613, 1488, 1456 (C=N, Ar-C=C, C=C); ^1^H-NMR (600 MHz, CDCl_3_): δ = 8.05 (td, *J* = 7.5, 1.8 Hz, 1H, H-17), 7.56–7.53 (m, 1H, H-15), 7.26–7.24 (m, 1H, H-14), 7.17 (dd, *J* = 10.3, 8.7 Hz, 1H, H-16), 6.55 – 6.50 (m, 1H, H-3), 3.03 (td, *J* = 6.0, 1.5 Hz, 1H, H-1), 2.76 (dt, *J* = 9.3, 5.5 Hz, 1H, H-5), 2.35–2.32 (m, 1H, H-7a), 1.99 (d, *J* = 1.5 Hz, 3H, H-10), 1.86 (d, *J* = 9.2 Hz, 1H, H-7b), 1.48 (s, 3H, H-9), 0.98 (s, 3H, H-8); ^13^C-NMR (150 MHz, CDCl_3_): δ = 167.0 (C-11), 164.1 (C-2), 134.6 (C-4), 134.5, 132.6 (C-13), 124.2 (C-15), 124.2 (C-17), 117.0 (C-16), 116.8 (C-12), 110.5 (C-14), 110.5 (C-3), 50.0 (C-1), 49.5 (C-6), 48.2 (C-5), 38.6 (C-7), 26.2 (C-8), 23.9 (C-10), 21.8 (C-9); ESI-MS *m*/*z*: 287.80 [M + H]^+^. Anal. calcd. For C_17_H_18_FNO_2_: C, 71.06; H, 6.31; N, 4.87; Found: C, 70.71; H, 6.25; N, 4.85.

*(E)-verbenone-based O-(2′-fluorobenzoyl) oxime* ((*E*)-**4g**). Faint yellow solid. Yield: 90.5%, melting point: 101.3–103.7 °C. UV-Vis (EtOH) λ_max_(log ε): 264.7 (4.34), 227.3 (4.23) nm; IR (KBr, cm^−1^): 3067, 3043 (Ar-H, =C-H), 2979, 2958, 2870 (C-H), 1741 (C=O), 1626, 1610, 1597 (C=N, Ar-C=C, C=C); ^1^H-NMR (600 MHz, CDCl_3_): δ = 8.01 (td, *J* = 7.5, 1.9 Hz, 1H, H-17), 7.54 (ddd, *J* = 15.4, 4.9, 1.8 Hz, 1H, H-15), 7.24 (td, *J* = 7.6, 1.1 Hz, 1H, H-14), 7.17–7.13 (m, 1H, H-16), 6.08 (d, *J* = 1.7 Hz, 1H, H-3), 3.71 (td, *J* = 5.8, 1.7 Hz, 1H, H-1), 2.69 (dt, *J* = 9.1, 5.5 Hz, 1H, H-5), 2.36–2.27 (m, 1H, H-7a), 1.96 (d, *J* = 1.6 Hz, 3H, H-10), 1.79 (d, *J* = 9.2 Hz, 1H, H-7b), 1.50 (s, 3H, H-9), 0.96 (s, 3H, H-8); ^13^C-NMR (150 MHz, CDCl_3_): δ = 169.2 (C-11), 160.0 (C-2), 134.6 (C-4), 134.6, 132.5 (C-13), 132.5 (C-15), 124.2 (C-17), 124.2 (C-16), 117.0 (C-12), 116.8 (C-14), 114.9 (C-3), 49.6 (C-1), 49.2 (C-6), 44.4 (C-5), 37.4 (C-7), 26.3 (C-8), 23.5 (C-10), 22.3 (C-9); ESI-MS *m*/*z*: 287.80 [M + H]^+^. Anal. calcd. For C_17_H_18_FNO_2_: C, 71.06; H, 6.31; N, 4.87; Found: C, 70.82; H, 6.26; N, 4.86.

*(Z)-verbenone O-(3′-methylbenzoyl) oxime* ((*Z*)-**4h**). brown liquid. Yield: 90.5%, melting point: 85.8–89.4 °C. UV-Vis (EtOH) λ_max_(log ε): 262.8 (4.28), 236.0 (4.29) nm; IR (KBr, cm^−1^): 3066 (Ar-H), 2953, 2933, 2865 (C-H), 1740 (C=O), 1619, 1590, 1490 (C=N, Ar-C=C, C=C); ^1^H-NMR (600 MHz, CDCl_3_): δ = 7.92 (s, 1H, H-17), 7.90 (d, *J* = 7.7 Hz, 1H, H-13), 7.39 (d, *J* = 7.6 Hz, 1H, H-16), 7.36 (d, *J* = 7.5 Hz, 1H, H-15), 6.49 (q, *J* = 1.6 Hz, 1H, H-3), 3.04 (td, *J* = 5.9, 1.7 Hz, 1H, H-1), 2.76 (dt, *J* = 9.2, 5.5 Hz, 1H, H-5), 2.43 (s, 3H, H-17), 2.36–2.32 (m, 1H, H-7a), 2.01 (d, *J* = 1.7 Hz, 3H, H-10), 1.86 (d, *J* = 9.2 Hz, 1H, H-7b), 1.48 (s, 3H, H-9), 0.98 (s, 3H, H-8); ^13^C-NMR (150 MHz, CDCl_3_): δ = 166.6 (C-11), 164.5 (C-2), 163.8 (C-4), 138.3 (C-14), 133.8 (C-15), 130.1 (C-13), 129.5 (C-12), 128.3 (C-17), 126.6 (C-16), 110.1 (C-3), 49.9 (C-1), 49.5 (C-6), 48.3 (C-5), 38.5 (C-7), 26.1 (C-8), 23.9 (C-10), 21.9 (C-9), 21.3 (C-18); ESI-MS *m*/*z*: 283.83 [M + H]^+^. Anal. calcd. For C_18_H_21_NO_2_: C, 76.30; H, 7.47; N, 4.94; Found: C, 75.95; H, 7.41; N, 4.92.

*(E)-verbenone O-(3′-methylbenzoyl) oxime* ((*E*)-**4h**). Faint brown liquid. Yield: 90.5%, melting point: 96.8–97.5 °C. UV-Vis (EtOH) λ_max_(log ε): 263.2 (4.28), 237.1 (4.25) nm; IR (KBr, cm^−1^): 3071 (Ar-H), 2957, 2928, 2870 (C-H), 1741 (C=O), 1619, 1592, 1508 (C=N, Ar-C=C, C=C); ^1^H-NMR (600 MHz, CDCl_3_): δ = 7.92 (s, 1H, H-17), 7.90 (d, *J* = 7.6 Hz, 1H, H-13), 7.39 (d, *J* = 7.6 Hz, 1H, H-16), 7.36 (t, *J* = 7.5 Hz, 1H, H-15), 6.49 (q, *J* = 1.6 Hz, 1H, H-3), 3.04 (td, *J* = 5.9, 1.7 Hz, 1H, H-1), 2.76 (dt, *J* = 9.3, 5.5 Hz, 1H, H-5), 2.43 (s, 3H, H-17), 2.36–2.33 (m, 1H, H-7a), 2.01 (d, *J* = 1.7 Hz, 3H, H-10), 1.86 (d, *J* = 9.2 Hz, 1H, H-7b), 1.48 (s, 3H, H-9), 0.98 (s, 3H, H-8); ^13^C-NMR (150 MHz, CDCl_3_): δ = 166.6 (C-11), 164.5 (C-2), 163.8 (C-4), 138.3 (C-14), 133.8 (C-15), 130.2 (C-13), 129.5 (C-12), 128.3 (C-17), 126.6 (C-16), 110.1 (C-3), 49.9 (C-1), 49.5 (C-6), 48.3 (C-5), 38.5 (C-7), 26.1 (C-8), 23.9 (C-10), 21.9 (C-9), 21.3 (C-18); ESI-MS *m*/*z*: 283.83 [M + H]^+^. Anal. calcd. For C_18_H_21_NO_2_: C, 76.30; H, 7.47; N, 4.94; Found: C, 76.05; H, 7.42; N, 4.93.

*(Z)-verbenone O-(3′-chlorobenzoyl) oxime* ((*Z*)-**4i**). Pink solid. Yield: 90.5%, melting point: 84.2–87.5 °C. UV-Vis (EtOH) λ_max_(log ε): 265.9 (4.17), 231.4 (4.10) nm; IR (KBr, cm^−1^): 3075 (Ar-H), 2999, 2937, 2869 (C-H), 1734 (C=O), 1619, 1571, 1469 (C=N, Ar-C=C, C=C); ^1^H-NMR (600 MHz, CDCl_3_): δ = 8.07 (t, *J* = 1.8 Hz, 1H, H-13), 8.00 (dt, *J* = 7.8, 1.3 Hz, 1H, H-17), 7.56 (ddd, *J* = 8.0, 2.1, 1.1 Hz, 1H, H-15), 7.43 (t, *J* = 7.9 Hz, 1H, H-16), 6.46 (q, *J* = 1.5 Hz, 1H, H-3), 3.03 (td, *J* = 6.0, 1.6 Hz, 1H, H-1), 2.77 (dt, *J* = 9.3, 5.5 Hz, 1H, H-5), 2.37–2.34 (m, 1H, H-7a), 2.02 (d, *J* = 1.6 Hz, 3H, H-10), 1.87 (d, *J* = 9.3 Hz, 1H, H-7b), 1.49 (s, 3H, H-9), 0.99 (s, 3H, H-8); ^13^C-NMR (150 MHz, CDCl_3_): δ = 167.1 (C-11), 164.5 (C-2), 163.2 (C-4), 134.6 (C-14), 133.1 (C-15), 131.4 (C-12), 129.8 (C-13), 129.6 (C-16), 127.7 (C-17), 109.9 (C-3), 50.1 (C-1), 49.6 (C-6), 48.3 (C-5), 38.6 (C-7), 26.1 (C-8), 24.0 (C-10), 21.9 (C-9); ESI-MS *m*/*z*: 303.76 [M + H]^+^. Anal. calcd. For C_17_H_18_ClNO_2_: C, 67.21; H, 5.97; N, 4.61; Found: C, 66.92; H, 5.91; N, 4.59.

*(E)-verbenone O-(3′-chlorobenzoyl) oxime* ((*E*)-**4i**). Faint pink solid. Yield: 90.5%, melting point: 89.4–94.8 °C. UV-Vis (EtOH) λ_max_(log ε): 265.1 (4.08), 232.5 (3.99) nm; IR (KBr, cm^−1^): 3077, 3048 (Ar-H, =C-H), 2983, 2959, 2871 (C-H), 1743 (C=O), 1632, 1601, 1569 (C=N, Ar-C=C, C=C); ^1^H-NMR (600 MHz, CDCl_3_): δ = 8.01 (s, 1H, H-13), 7.93 (d, *J* = 7.8 Hz, 1H, H-17), 7.57 – 7.53 (m, 1H, H-15), 7.41 (t, *J* = 7.9 Hz, 1H, H-16), 6.14–6.03 (m, 1H, H-3), 3.64 (td, *J* = 5.8, 1.5 Hz, 1H, H-1), 2.72 (dt, *J* = 9.2, 5.5 Hz, 1H, H-5), 2.36–2.31 (m, 1H, H-7a), 1.97 (d, *J* = 1.3 Hz, 3H, H-10), 1.82 (d, *J* = 9.2 Hz, 1H, H-7b), 1.53 (s, 3H, H-9), 0.98 (s, 3H, H-8); ^13^C-NMR (150 MHz, CDCl_3_): δ = 169.3 (C-11), 163.2 (C-2), 156.0 (C-4), 134.6 (C-14), 133.1 (C-15), 131.3 (C-12), 129.8 (C-13), 129.6 (C-16), 127.7 (C-17), 115.0 (C-3), 49.6 (C-1), 49.1 (C-6), 44.1 (C-5), 37.5 (C-7), 26.3 (C-8), 23.5 (C-10), 22.3 (C-9); ESI-MS *m*/*z*: 303.73 [M + H]^+^. Anal. calcd. For C_17_H_18_ClNO_2_: C, 67.21; H, 5.97; N, 4.61; Found: C, 67.10; H, 5.94; N, 4.60.

*(Z)-verbenone O-(4′-bromobenzoyl) oxime* ((*Z*)-**4j**). White solid. Yield: 90.5%, melting point: 124.6–128.0°C. UV-Vis (EtOH) λ_max_(log ε): 262.6 (4.29), 249.2 (4.30) nm; IR (KBr, cm^−1^): 3093, 3074 (Ar-H, =C-H), 2963, 2943, 2869 (C-H), 1737 (C=O), 1619, 1586, 1482 (C=N, Ar-C=C, C=C); ^1^H-NMR (600 MHz, CDCl_3_): δ = 8.00–7.94 (m, 2H, H-13,17), 7.67–7.59 (m, 2H, H-14,16), 6.45 (q, *J* = 1.4 Hz, 1H, H-3), 3.03 (td, *J* = 5.8, 1.7 Hz, 1H, H-1), 2.77 (dt, *J* = 9.3, 5.4 Hz, 1H, H-5), 2.40–2.30 (m, 1H, H-7a), 2.01 (d, *J* = 1.6 Hz, 3H, H-10), 1.86 (d, *J* = 9.2 Hz, 1H, H-7b), 1.48 (s, 3H, H-9), 0.98 (s, 3H, H-8); ^13^C-NMR (150 MHz, CDCl_3_): δ = 166.9 (C-11), 164.3 (C-2), 163.6 (C-4), 131.8 (C-13,17), 131.1 (C-14,16), 128.5 (C-12), 128.1 (C-15), 109.9 (C-3), 50.0 (C-1), 49.5 (C-6), 48.3 (C-5), 38.6 (C-7), 26.1 (C-8), 24.0 (C-10), 21.8 (C-9); ESI-MS *m*/*z*: 347.65 [M + H]^+^. Anal. calcd. For C_17_H_18_BrNO_2_: C, 58.63; H, 5.21; N, 4.02; Found: C, 58.39; H, 5.17; N, 4.00.

*(Z)-verbenone O-(2′,3′-dichlorobenzoyl) oxime* ((*Z*)-**4k**). Yellow solid. Yield: 90.5%, melting point: 50.2–52.3 °C. UV-Vis (EtOH) λ_max_(log ε): 262.4 (4.26), 204.9 (4.61) nm; IR (KBr, cm^−1^): 3074 (Ar-H), 2953, 2933, 2868 (C-H), 1754 (C=O), 1650, 1622, 1593 (C=N, Ar-C=C, C=C); ^1^H-NMR (600 MHz, CDCl_3_): δ = 7.71–7.68 (m, 1H, H-15), 7.64–7.61 (m, 1H, H-17), 7.31 (s, 1H, H-16), 6.44 (d, *J* = 1.4 Hz, 1H, H-3), 3.02 (td, *J* = 6.0, 1.5 Hz, 1H, H-1), 2.78 (dt, *J* = 9.4, 5.5 Hz, 1H, H-5), 2.35 (t, *J* = 5.3 Hz, 1H, H-7a), 1.99 (d, *J* = 1.3 Hz, 3H, H-10), 1.88 (d, *J* = 9.2 Hz, 1H, H-7b), 1.50 (s, 3H, H-9), 0.99 (s, 3H, H-8); ^13^C-NMR (150 MHz, CDCl_3_): δ = 167.2 (C-11), 164.6 (C-2), 163.7 (C-4), 134.4 (C-14), 133.0 (C-15), 132.8 (C-12), 131.3 (C-13), 129.1 (C-17), 127.3 (C-16), 110.2 (C-3), 50.2 (C-1), 49.5 (C-6), 48.0 (C-5), 38.6 (C-7), 26.1 (C-8), 23.9 (C-10), 21.8 (C-9); ESI-MS *m*/*z*: 337.66 [M + H]^+^. Anal. calcd. For C_17_H_17_Cl_2_NO_2_: C, 60.37; H, 5.07; N, 4.14; Found: C, 60.07; H, 5.02; N, 4.12.

*(E)*-*verbenone O-(2′,3′-dichlorobenzoyl) oxime* ((*E*)-**4k**). Faint yellow solid. Yield: 90.5%, melting point: 62.5–63.8 °C. UV-Vis (EtOH) λ_max_(log ε): 261.4 (4.22), 204.8 (4.58) nm; IR (KBr, cm^−1^): 3078, 3045 (Ar-H, =C-H), 2954, 2932, 2868 (C-H), 1754 (C=O), 1625, 1595, 1558 (C=N, Ar-C=C, C=C); ^1^H-NMR (600 MHz, CDCl_3_): δ = 7.64 (d, *J* = 7.7 Hz, 1H, H-15), 7.60 (d, *J* = 8.0 Hz, 1H, H-17), 7.29 (d, *J* = 7.9 Hz, 1H, H-16), 6.05 (s, 1H, H-3), 3.62 (td, *J* = 5.8, 1.7 Hz, 1H, H-1), 2.66 (dt, *J* = 9.0, 5.5 Hz, 1H, H-5), 2.32 (t, *J* = 5.6 Hz, 1H, H-7a), 1.96 (s, 3H, H-10), 1.79 (s, 1H, H-7b), 1.48 (s, 3H, H-9), 0.96 (s, 3H, H-8); ^13^C-NMR (150 MHz, CDCl_3_): δ = 169.6 (C-11), 163.8 (C-2), 160.5 (C-4), 134.4 (C-14), 133.1 (C-15), 132.7 (C-12), 131.2 (C-13), 129.1 (C-17), 127.4 (C-16), 114.6 (C-3), 49.8 (C-1), 49.2 (C-6), 44.3 (C-5), 37.4 (C-7), 26.2 (C-8), 23.5 (C-10), 22.3 (C-9); ESI-MS *m*/*z*: 337.65 [M + H]^+^. Anal. calcd. For C_17_H_17_Cl_2_NO_2_: C, 60.37; H, 5.07; N, 4.14; Found: C, 60.12; H, 5.04; N, 4.13.

*(Z)-verbenone O-(2′,4′-dichlorobenzoyl) oxime* ((*Z*)-**4l**). White solid. Yield: 90.5%, melting point: 78.9–80.3 °C. UV-Vis (EtOH) λ_max_(log ε): 262.8 (4.16), 206.7 (4.45) nm; IR (KBr, cm^−1^): 3060, 3022 (Ar-H, =C-H), 2934, 2902, 2870 (C-H), 1755 (C=O), 1622, 1581, 1553 (C=N, Ar-C=C, C=C); ^1^H-NMR (600 MHz, CDCl_3_): δ = 7.83 (d, *J* = 8.4 Hz, 1H, H-17), 7.50 (d, *J* = 2.0 Hz, 1H, H-14), 7.34 (dd, *J* = 8.4, 2.0 Hz, 1H, H-16), 6.50–6.39 (m, 1H, H-3), 3.01 (td, *J* = 6.0, 1.4 Hz, 1H, H-1), 2.76 (dt, *J* = 9.3, 5.5 Hz, 1H, H-5), 2.34 (t, *J* = 5.7 Hz, 1H, H-7a), 1.98 (d, *J* = 1.5 Hz, 3H, H-10), 1.86 (d, *J* = 9.3 Hz, 1H, H-7b), 1.48 (s, 3H, H-9), 0.97 (s, 3H, H-8); ^13^C-NMR (150 MHz, CDCl_3_): δ = 167.1 (C-11), 164.5 (C-2), 163.2 (C-4), 138.2 (C-15), 134.4 (C-13), 132.6 (C-17), 130.8 (C-12), 128.4 (C-14), 127.1 (C-16), 110.4 (C-3), 50.1 (C-1), 49.5 (C-6), 48.2 (C-5), 38.6 (C-7), 26.1 (C-8), 24.0 (C-10), 21.8 (C-9); ESI-MS *m*/*z*: 337.74 [M + H]^+^. Anal. calcd. For C_17_H_17_Cl_2_NO_2_: C, 60.37; H, 5.07; N, 4.14; Found: C, 60.09; H, 5.03; N, 4.12.

*(E)-verbenone O-(2′,4′-dichlorobenzoyl) oxime* ((*E*)-**4l**). Faint yellow liquid. Yield: 90.5%, melting point: 112.7–121.9 °C. UV-Vis (EtOH) λ_max_(log ε): 262.1 (4.26), 207.1 (4.57) nm; IR (KBr, cm^−1^): 3084 (Ar-H), 2956, 2927, 2870 (C-H), 1752 (C=O), 1629, 1586, 1557 (C=N, Ar-C=C, C=C); ^1^H-NMR (600 MHz, CDCl_3_): δ = 7.78 (d, *J* = 8.4 Hz, 1H, H-17), 7.48 (d, *J* = 2.0 Hz, 1H, H-14), 7.33 (dd, *J* = 8.4, 2.0 Hz, 1H, H-16), 6.06 (q, *J* = 1.6 Hz, 1H, H-3), 3.64 (td, *J* = 5.8, 1.7 Hz, 1H, H-1), 2.67 (dt, *J* = 9.2, 5.5 Hz, 1H, H-5), 2.32 (td, *J* = 5.9, 1.4 Hz, 1H, H-7a), 1.96 (d, *J* = 1.6 Hz, 3H, H-10), 1.78 (d, *J* = 9.2 Hz, 1H, H-7b), 1.48 (s, 3H, H-9), 0.96 (s, 3H, H-8); ^13^C-NMR (150 MHz, CDCl_3_): δ = 169.5 (C-11), 163.3 (C-2), 160.3 (C-4), 138.3 (C-15), 134.3 (C-13), 132.6 (C-17), 130.8 (C-12), 128.4 (C-14), 127.2 (C-16), 114.7 (C-3), 49.7 (C-1), 49.1 (C-6), 44.5 (C-5), 37.4 (C-7), 26.3 (C-8), 23.5 (C-10), 22.3 (C-9); ESI-MS *m*/*z*: 337.67 [M + H]^+^. Anal. calcd. For C_17_H_17_Cl_2_NO_2_: C, 60.37; H, 5.07; N, 4.14; Found: C, 60.12; H, 5.04; N, 4.13.

*(Z)*-*verbenone O-2-chloropyridylcarbonyl oxime* ((*Z*)-**4m**). White solid. Yield: 90.5%, melting point: 84.1–87.1 °C. UV-Vis (EtOH) λ_max_(log ε): 261.5 (4.39), 219.7 (4.34) nm; IR (KBr, cm^−1^): 3077 (=C-H), 2955, 2871 (C-H), 1758 (C=O), 1621, 1600 (C=N, C=C); ^1^H-NMR (600 MHz, CDCl_3_): δ = 8.55 (dd, *J* = 4.8, 2.0 Hz, 1H, H-14), 8.20 (dd, *J* = 7.6, 2.0 Hz, 1H, H-16), 7.38 (dd, *J* = 7.7, 4.8 Hz, 1H, H-15), 6.48 (q, *J* = 1.5 Hz, 1H, H-3), 3.01 (td, *J* = 6.0, 1.6 Hz, 1H, H-1), 2.78 (dt, *J* = 9.3, 5.5 Hz, 1H, H-5), 2.37–2.33 (m, 1H, H-7a), 1.99 (d, *J* = 1.6 Hz, 3H, H-10), 1.87 (d, *J* = 9.3 Hz, 1H, H-7b), 1.49 (s, 3H, H-9), 0.98 (s, 3H, H-8); ^13^C-NMR (150 MHz, CDCl_3_): δ = 167.4 (C-11), 164.9 (C-2), 163.0 (C-4), 151.8 (C-14), 149.5 (C-13), 140.5 (C-15), 127.1 (C-12), 122.2 (C-16), 110.3 (C-3), 50.3 (C-1), 49.5 (C-6), 48.2 (C-5), 38.7 (C-7), 26.1 (C-8), 24.0 (C-10), 21.8 (C-9); ESI-MS *m*/*z*: 313.24 [M + H]^+^. Anal. calcd. For C_16_H_17_ClN_2_O_2_: C, 63.06; H, 5.62; N, 9.19; Found: C, 62.81; H, 5.57; N, 9.14.

*(E)-verbenone O-2-chloropyridylcarbonyl oxime* ((*E*)-**4m**). Faint yellow solid. Yield: 90.5%, melting point: 123.7–126.5 °C. UV-Vis (EtOH) λ_max_(log ε): 266.1(4.30), 218.8 (4.13) nm; IR (KBr, cm^−1^): 3074 (=C-H), 2964, 2943, 2866 (C-H), 1756 (C=O), 1622, 1578 (C=N, C=C); ^1^H-NMR (600 MHz, CDCl_3_): δ = 8.55 (dd, *J* = 4.8, 2.0 Hz, 1H, H-14), 8.20 (dd, *J* = 7.6, 2.0 Hz, 1H, H-16), 7.38 (dd, *J* = 7.7, 4.8 Hz, 1H, H-15), 6.48 (q, *J* = 1.5 Hz, 1H, H-3), 3.01 (td, *J* = 5.9, 1.7 Hz, 1H, H-1), 2.78 (dt, *J* = 9.3, 5.5 Hz, 1H, H-5), 2.38–2.33 (m, 1H, H-7a), 1.99 (d, *J* = 1.6 Hz, 3H, H-10), 1.87 (d, *J* = 9.3 Hz, 1H, H-7b), 1.49 (s, 3H, H-9), 0.98 (s, 3H, H-8); ^13^C-NMR (150 MHz, CDCl_3_): δ = 167.4 (C-11), 164.9 (C-2), 163.0 (C-4), 151.8 (C-14), 149.5 (C-13), 140.5 (C-15), 127.1 (C-12), 122.2 (C-16), 110.3 (C-3), 50.3 (C-1), 49.5 (C-6), 48.2 (C-5), 38.7 (C-7), 26.1 (C-8), 24.0 (C-10), 21.8 (C-9); ESI-MS *m*/*z*: 312.88 [M + H]^+^. Anal. calcd. For C_16_H_17_ClN_2_O_2_: C, 63.06; H, 5.62; N, 9.19; Found: C, 62.85; H, 5.58; N, 9.16.

*(Z)-verbenone O-β-pyridylcarbonyl oxime* ((*Z*)-**4n**). Brown solid. Yield: 90.5%, melting point: 75.2–76.9 °C. UV-Vis (EtOH) λ_max_(log ε): 266.1 (4.45), 220.2 (4.34) nm; IR (KBr, cm^−1^): 3053 (=C-H), 2980, 2952, 2876 (C-H), 1745 (C=O), 1619, 1589, 1482 (C=N, C=C); ^1^H-NMR (600 MHz, CDCl_3_): δ = 9.31 (d, *J* = 1.7 Hz, 1H, H-13), 8.83 (dd, *J* = 4.9, 1.6 Hz, 1H, H-14), 8.44 (dt, *J* = 7.9, 1.9 Hz, 1H, H-16), 7.50 (dd, *J* = 7.9, 4.9 Hz, 1H, H-14), 6.61–6.36 (m, 1H, H-3), 3.03 (td, *J* = 6.0, 1.5 Hz, 1H, H-1), 2.78 (dt, *J* = 9.3, 5.5 Hz, 1H, H-5), 2.38–2.36 (m, 1H, H-7a), 2.03 (d, *J* = 1.5 Hz, 3H, H-10), 1.88 (d, *J* = 9.3 Hz, 1H, H-7b), 1.50 (s, 3H, H-9), 0.99 (s, 3H, H-8); ^13^C-NMR (150 MHz, CDCl_3_): δ = 167.3 (C-11), 164.9 (C-2), 162.7 (C-4), 152.8 (C-14), 150.0 (C-13), 137.8 (C-16), 126.0 (C-12), 123.8 (C-15), 109.8 (C-3), 50.2 (C-1), 49.6 (C-6), 48.3 (C-5), 38.7 (C-7), 26.1 (C-8), 24.0 (C-10), 21.8 (C-9); ESI-MS *m*/*z*: 270.83 [M + H]^+^. Anal. calcd. For C_16_H_18_N_2_O_2_: C, 71.09; H, 6.71; N, 10.36; Found: C, 70.80; H, 6.64; N, 10.31.

*(E)-verbenone O-β-pyridylcarbonyl oxime* ((*E*)-**4n**). Faint yellow solid. Yield: 90.5%, melting point: 77.5–82.4 °C. UV-Vis (EtOH) λ_max_(log ε): 261.5(4.39), 219.7 (4.34) nm; IR (KBr, cm^−1^): 3060 (=C-H), 2991, 2965, 2871 (C-H), 1748 (C=O), 1621, 1586 (C=N, C=C); ^1^H-NMR (600 MHz, CDCl_3_): δ = 9.23 (d, *J* = 1.5 Hz, 1H, H-13), 8.80 (dd, *J* = 4.8, 1.6 Hz, 1H, H-14), 8.34 (dt, *J* = 7.9, 1.9 Hz, 1H, H-16), 7.44 (ddd, *J* = 7.9, 4.9, 0.8 Hz, 1H, H-14), 6.13–6.04 (m, 1H, H-3), 3.66 (td, *J* = 5.8, 1.6 Hz, 1H, H-1), 2.72 (dt, *J* = 9.2, 5.5 Hz, 1H, H-5), 2.34 (td, *J* = 5.7, 1.6 Hz, 1H, H-7a), 1.97 (d, *J* = 1.5 Hz, 3H, H-10), 1.82 (d, *J* = 9.2 Hz, 1H, H-7b), 1.52 (s, 3H, H-9), 0.98 (s, 3H, H-8); ^13^C-NMR (150 MHz, CDCl_3_): δ = 169.5 (C-11), 163.0 (C-2), 160.3 (C-4), 153.5 (C-14), 150.6 (C-13), 137.2 (C-16), 125.6 (C-12), 123.5 (C-15), 114.8 (C-3), 49.7 (C-1), 49.1 (C-6), 44.1 (C-5), 37.6 (C-7), 26.3 (C-8), 23.5 (C-10), 22.3 (C-9); ESI-MS *m*/*z*: 270.75 [M + H]^+^. Anal. calcd. For C_16_H_18_N_2_O_2_: C, 71.09; H, 6.71; N, 10.36; Found: C, 70.83; H, 6.66; N, 10.33.

### 3.5. Antifungal Activity Test

This test was performed according to the literature [30]. The tested compound was dissolved in acetone. Sorporl-144 (200 μg/mL) emulsifier was added to dilute the solution to 500 μg/mL. Then, 1 mL solution of the tested compound was poured into a culture plate, and then 9 mL Potato-Sugar-Agar (PSA) culture medium was added to obtain flats containing 50 μg/mL of test compound. A bacterium tray of 5-mm diameter cut along the external edge of the mycelium was transferred to the flat containing the tested compound and put in equilateral triangular style in triplicate. Later, the culture plate was cultured at 24 ± 1 °C and the expanded diameter of the bacterium tray was measured after 48 h and compared with that treated with aseptic distilled water to calculate the relative inhibition percentage. The current commercial fungicide chlorothalonil was used as a positive control.

### 3.6. Herbicidal Activity Test

#### 3.6.1. Inhibition of the Root-growth of Rape (*B. campestris*)

This test was carried out according to the literature [30]. The compounds to be tested were made into emulsions by using Tween-80 as emulsifying agent to aid dissolution at concentrations of 10 μg/mL and 100 μg/mL. Groups of 15 seeds of rape (*B. campestris*) were placed on a 5.6-cm filter paper that was in 6-cm Petri dishes containing 2 mL of compound solutions. Equal volume of distilled water was used as control. Petri dishes were placed in darkness at 28 ± 1 °C for 72 h. The radicle lengths of seedlings were measured. All experiments had three replicates. The inhibition percent of average length to control was used to describe the activity of compounds. And the current commercial herbicide flumioxazin was used as a positive control.

#### 3.6.2. Inhibition of the Seedling Growth of Barnyard Grass (*E.* *crusgalli*)

This test was performed according to the literature [30]. The compounds to be evaluated were made into emulsions by using TW-80 as emulsifying agent to aid dissolution, at concentrations of 10 μg/mL and 100 μg/mL. Groups of 10 germinated seeds of barnyard grass *E. crusgalli* were placed on a filter paper that was in a 50 mL beaker containing 6 mL of compound solutions. Equal volume of distilled water was used as control. Beakers were placed at 28 ± 1 °C (3000 lux) for 72 h. The heights of seedlings were measured. All experiments had three replicates. The inhibition percent of average height to control was used to describe the activity of compounds, and the current commercial herbicide flumioxazin was used as a positive control.

## 4. Conclusions

Twenty-seven novel (*Z*)- and (*E*)-verbenone oxime esters were designed, synthesized, characterized, and evaluated for their antifungal and herbicidal activities. Compound (*E*)-**4n** exhibited excellent antifungal activity, with growth inhibitions of 92.2%, 80.0% and 76.3% against *A. solani*, *P. piricola* and *C. arachidicola*, respectively, displaying better or comparable antifungal activity than that of the positive control with inhibition rates of 96.1%, 75.0%, and 73.3%, respectively. Meanwhile, this compound (*E*)-**4n** also showed 5.5-, 4.1-, 2.8-, and 1.7-fold higher antifungal activity against *F. oxysporum* f. sp. *cucumerinum*, *R. solani*, *P. piricola*, and *C. arachidicola*, respectively, than its stereoisomer (*Z*)-**4n**. Seventeen target compounds displayed better herbicidal activity with 63.6–99.3% inhibition rates against the root-growth of rape (*B. campestris*) than that of the commercial herbicidal flumioxazin (positive control) with inhibition rate of 63.0%. And compound (*E*)-**4n** showed 1.7-fold herbicidal activity to its stereoisomer (*Z*)-**4n**. Thus, the target compound (*E*)-**4n** with excellent antifungal and herbicidal activities, as well as obvious bioactive difference between (*Z*)- and (*E*)-isomers, can serve as lead compounds worthy of further study.

## Figures and Tables

**Table 1:** Antifungal activity of the target compounds (*Z*)- and (*E*)-**4a**–**4n** at 50 µg/mL.

Compounds	Fungal Growth Inhibition (%)								
	*F. oxysporum f. sp. cucumerinum*	*C. arachidicola*	*P. piricola*	*A. solani*	*G. zeae*	*R. solani*	*B. maydis*	*C. orbiculare*	Average(I)
*(Z)*-**4a** (R = *n*-butyl)	28.6	32.8	45.7	37	28.6	48.7	24	21.1	33.3
*(E)*-**4a** (R = *n*-butyl)	17.2	10.9	40	37	28.6	22.6	18	7.1	22.7
*(Z)*-**4b** (R = *n*-amyl)	34.3	10.9	17.2	9.2	11.4	48.7	24	7.1	20.4
*(E)*-**4b** (R = *n*-amyl)	17.2	21.8	45.7	27.7	28.6	31.3	24	7.1	25.4
*(Z)*-**4c** (R = cyclopentyl)	57.1	43.7	45.7	27.7	22.8	76.6	24	28.2	40.7
*(E)*-**4c** (R = cyclopentyl)	28.6	32.8	17.2	9.2	11.4	48.7	24	7.1	22.4
*(Z)*-**4d** (R = cyclohexyl)	21.5	12	36.4	43.2	21.8	0	32	26.6	24.2
*(E)*-**4d** (R = cyclohexyl)	17.2	24	0	43.2	16.3	0	20	26.6	18.4
*(Z)*-**4e** (R = 2′-CH_3_ Ph)	21.5	24	50.9	52.8	35.4	0	20	57.7	32.8
*(E)*-**4e** (R = 2′-CH_3_ Ph)	17.2	24	50.9	52.8	16.3	0	12	66.7	30
*(Z)*-**4f** (R = 2′-Cl Ph)	12.8	24	43.7	24	8.2	0	16	40	21.1
*(E)*-**4f** (R = 2′-Cl Ph)	8.5	18	3.6	33.6	19.1	0	16	35.5	16.8
*(Z)*-**4g** (R = 2′-F Ph)	12.8	12	25.4	24	5.4	0	16	22.2	14.7
*(E)*-**4g** (R = 2′-F Ph)	17.2	24	3.6	43.2	21.8	0	16	44.4	21.3
*(Z)*-**4h** (R = 3′-CH_3_ Ph)	40	54.6	45.7	9.2	22.8	57.4	24	56.5	38.8
*(E)*-**4h** (R = 3′-CH_3_ Ph)	45.7	65.4	34.3	18.5	28.6	62.6	30	70.6	44.5
*(Z)*-**4i** (R = 3′-Cl Ph)	64.3	24	32.8	67.2	46.3	14.4	32	53.3	41.8
*(E)*-**4i** (R = 3′-Cl Ph)	25.7	12	25.4	33.6	30	0	32	44.4	25.4
*(Z)*-**4j** (R = 4′-Br Ph)	42.8	24	65.4	38.4	32.8	2.9	28	44.4	34.8
*(Z)*-**4k** (R = 2′,3′-Cl Ph)	40	32.8	11.4	37	28.6	36.5	12	7.1	25.7
*(E)*-**4k** (R = 2′,3′-Cl Ph)	28.6	10.9	11.4	18.5	28.6	31.3	18	7.1	19.3
*(Z)*-**4l** (R = 2′,4′-Cl Ph)	30	12	25.4	28.8	8.2	0	16	40	20.1
*(E)*-**4l** (R = 2′,4′-Cl Ph)	8.5	30	32.8	52.8	10.9	0	12	22.2	21.2
*(Z)*-**4m** (R = *α*-Cl-*β*-pyridyl)	17.2	32.8	34.3	9.2	28.6	62.6	12	7.1	25.5
*(E)*-**4m** (R = *α*-Cl-*β*-pyridyl)	17.2	32.8	51.5	18.5	11.4	57.4	12	7.1	26
*(Z)*-**4n** (R = *β*-pyridyl)	11.4	43.7	28.6	9.2	11.4	22.6	12	21.1	20
*(E)*-**4n** (R = *β*-pyridyl)	62.9	76.3	80	46.2	28.6	92.2	42	77.6	63.2
*Average(II)*	27.6	28.4	33.5	31.5	21.9	26.5	21	31.7	-
(*Z*)-oxime **3**	13.3	10.9	38.8	52.6	35.3	42.8	22.8	13.3	-
(*E*)-oxime **3**	16	10.9	38.8	30	21.1	42.8	28.6	13.3	-
verbenone **2**	16.4	26.7	58.1	16.3	58.7	33.5	20.8	27	-
Chlorothalonil	100	73.3	75	73.9	73.1	96.1	90.4	91.3	-

Chlorothalonil, a current commercial fungicide, was used as a positive control. Values are the average of three replicates.

**Table 2:** Herbicidal activity of the target compounds (*Z*)- and (*E*)-**4a**–**4n** at 10 µg/mL and 100 µg/mL.

Compounds	Growth Inhibition (％)			
	*B. campestris*		*E. crusgalli*	
	10 μg/mL	100 μg/mL	10 μg/mL	100 μg/mL
*(Z)*-**4a** (R = *n*-butyl)	0	76.7	0	0
*(E)*-**4a** (R = *n*-butyl)	0	59.3	11.0	16.5
*(Z)*-**4b** (R = *n*-amyl)	0	48.5	0	0
*(E)*-**4b** (R = *n*-amyl)	0	27.0	0	5.5
*(Z)*-**4c** (R = cyclopentyl)	8.1	76.3	0	0
*(E)*-**4c** (R = cyclopentyl)	0	77.4	0	0
*(Z)*-**4d** (R = cyclohexyl)	0	74.3	0	0
*(E)*-**4d** (R = cyclohexyl)	0	68.1	0	5.5
*(Z)*-**4e** (R = 2′-CH_3_ Ph)	14.1	60.5	0	0
*(E)*-**4e** (R = 2′-CH_3_ Ph)	31.2	63.6	0	0
*(Z)*-**4f** (R = 2′-Cl Ph)	19.1	66.6	0	0
*(E)*-**4f** (R = 2′-Cl Ph)	38.6	54.5	0	0
*(Z)*-**4g** (R = 2′-F Ph)	42.1	95.9	0	0
*(E)*-**4g** (R = 2′-F Ph)	8.6	76.0	0	3.3
*(Z)*-**4h** (R = 3′-CH_3_ Ph)	31.2	90.1	0	5.5
*(E)*-**4h** (R = 3′-CH_3_ Ph)	63.6	92.6	0	0
*(Z)*-**4i** (R = 3′-Cl Ph)	71.7	96.9	0	0
*(E)*-**4i** (R = 3′-Cl Ph)	62.0	88.0	0	0
*(Z)*-**4j** (R = 4′-Br Ph)	41.7	79.9	0	0
*(Z)*-**4k** (R = 2′,3′-Cl Ph)	83.8	96.6	0	0
*(E)*-**4k** (R = 2′,3′-Cl Ph)	82.7	99.3	0	0
*(Z)*-**4l** (R = 2′,4′-Cl Ph)	49.5	52.5	0	0
*(E)*-**4l** (R = 2′,4′-Cl Ph)	34.8	57.8	0	0
*(Z)*-**4m** (R = *α*-Cl-*β*-pyridyl)	16.7	60.3	0	29.4
*(E)*-**4m** (R = *α*-Cl-*β*-pyridyl)	5.1	57.9	0	0
*(Z)*-**4n** (R = *β*-pyridyl)	23.0	54.0	0	0
*(E)*-**4n** (R = *β*-pyridyl)	58.1	92.1	0	0
(*Z*)-oxime **3**	27.7	62.2	0	11.0
(*E*)-oxime **3**	46.4	72.8	11.0	22.0
verbenone **2**	0	16.3	0	12.7
Flumioxazin	57.8	63.0	95.1	97.5

Flumioxazin, a current commercial herbicide was used as a positive control Values are the average of three replicates.

## Supplementary Materials

The following are available online. Figure S1: UV-vis spectrum of verbenone **2** in EtOH.
