# Supplementary material for: Synthesis and Biological Activity of Novel (Z)- and (E)-Verbenone Oxime Esters
Source: Molecules. 2017 Oct 12;22(10):1678. doi: 10.3390/molecules22101678 (PMC6151715; doi:10.3390/molecules22101678)
Supplement: Supplementary file 1 [file molecules-22-01678-s001.pdf]

Synthesis and Biological Activity of Novel (*Z*)- and (*E*)-Verbenone Oxime Esters

Qiong Hu, Wen-Gui Duan *, Gui-Shan Lin *, Min Huang and Fu-Hou Lei

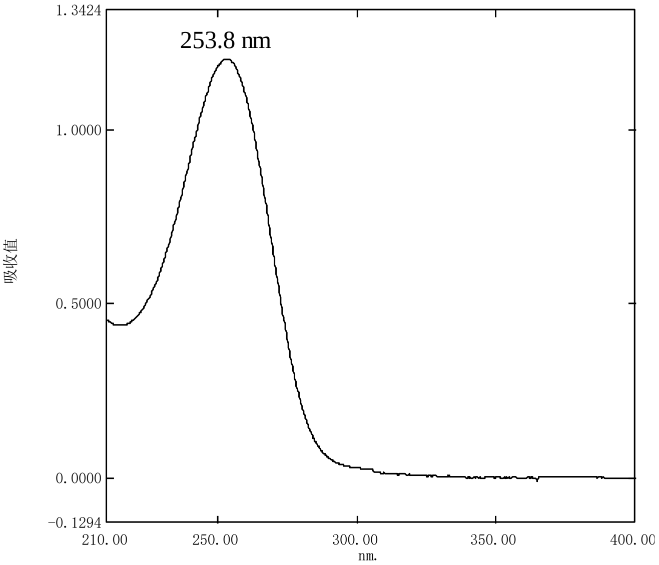

Figure S1. UV-vis spectrum of verbenone **2** in EtOH.

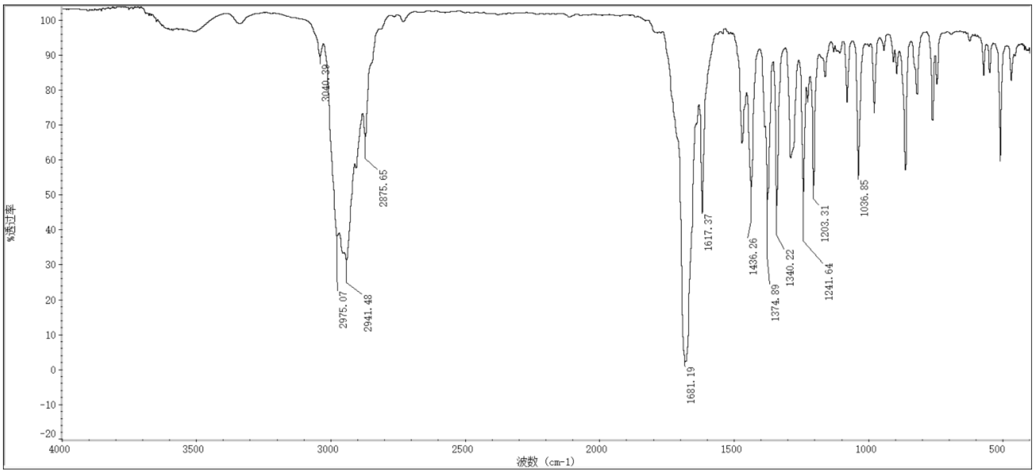

Figure S2. FTIR spectrum of verbenone **2**.

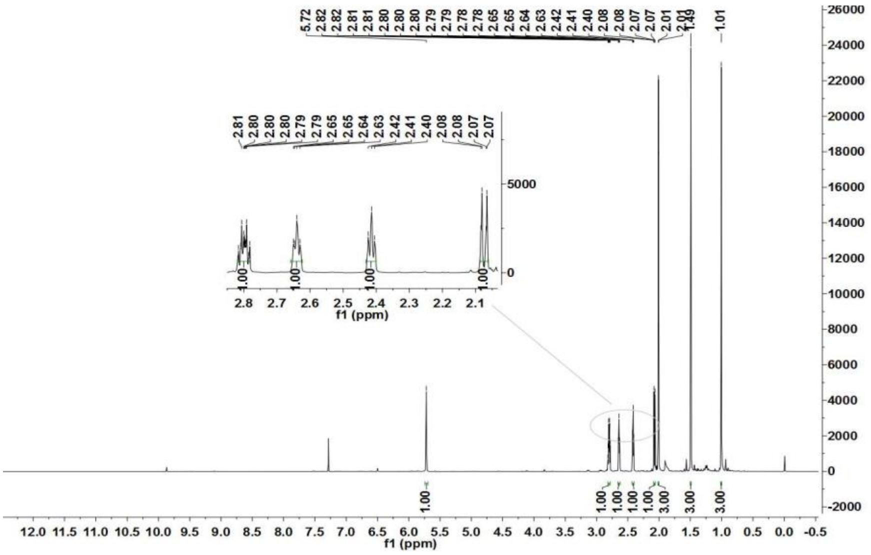

Figure S3. ^{1}H-NMR spectrum of verbenone **2** in $CDCl_3$.

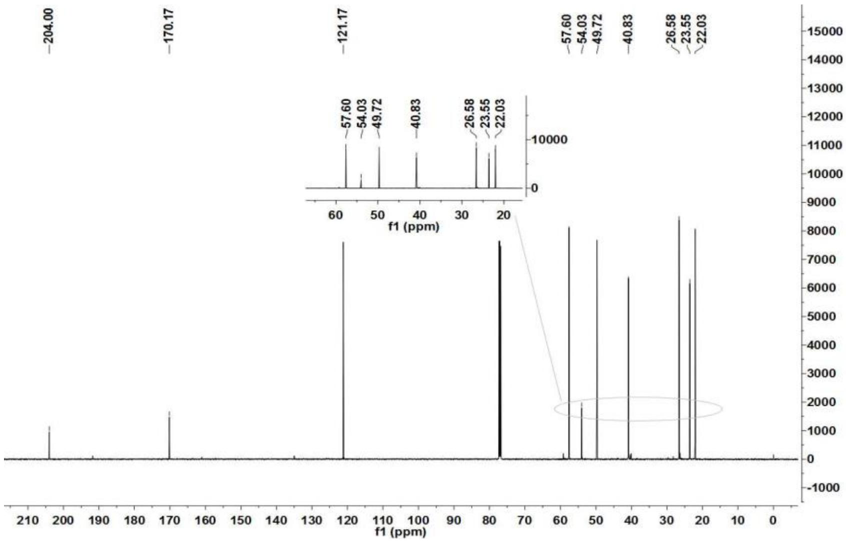

Figure S4. ^{13}C-NMR spectrum of verbenone **2** in $CDCl_3$.

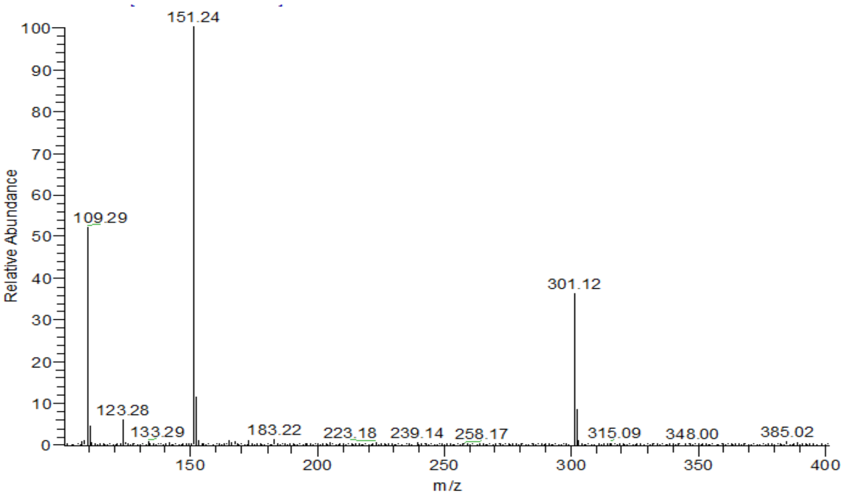

Figure S5. ESI-MS spectrum of verbenone **2**.

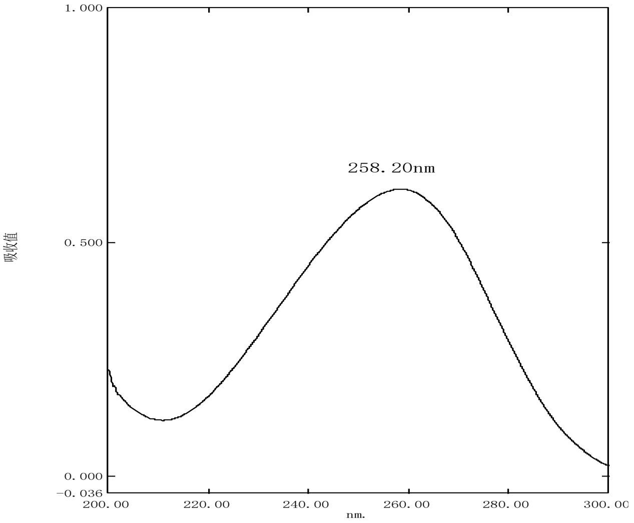

Figure S11. UV-vis spectrum of (*Z*)-verbenone oxime **3** in EtOH.

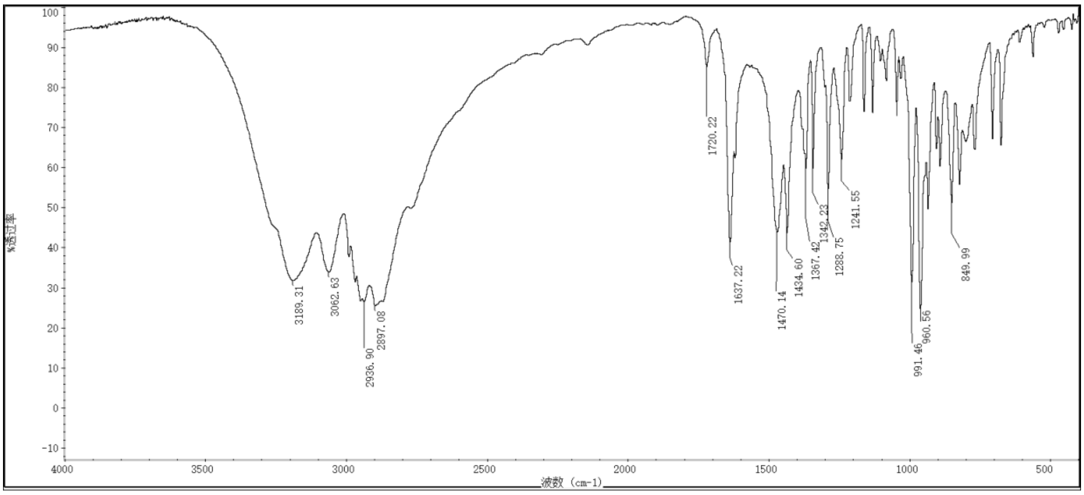

Figure S12. FTIR spectrum of (*Z*)-verbenone oxime **3**.

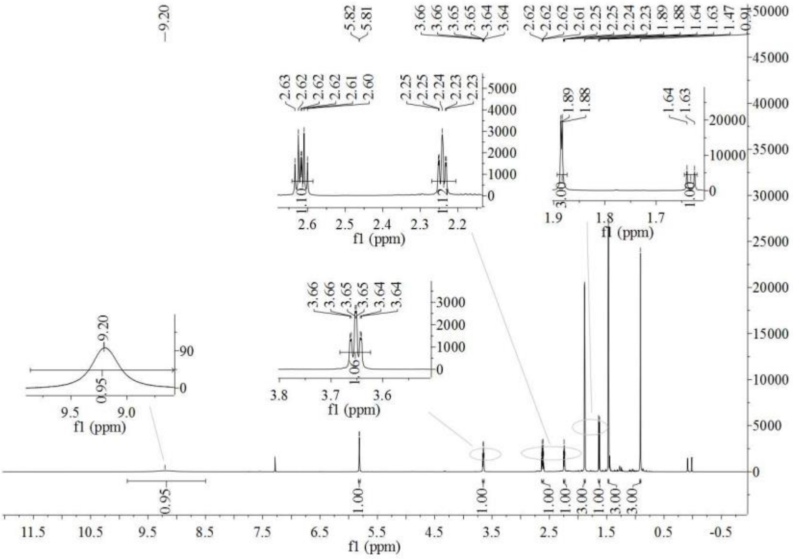

Figure S13. ^{1}H-NMR spectrum of (*Z*)-verbenone oxime **3** in $CDCl_3$.

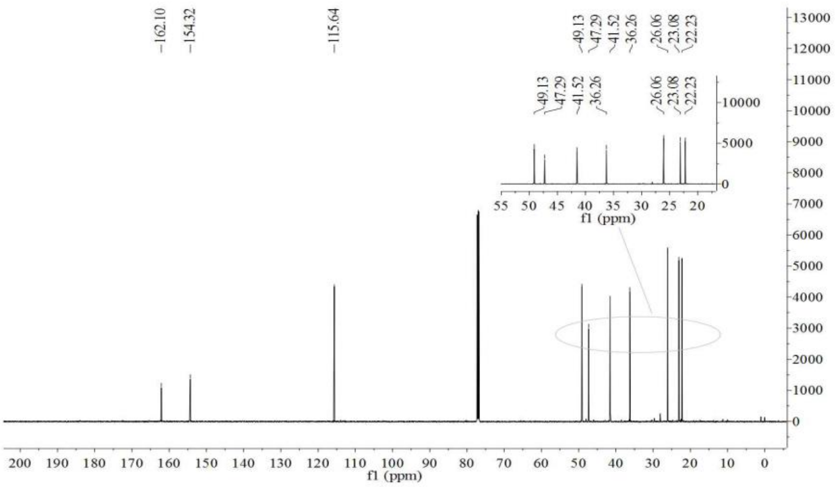

Figure S14. ^{13}C-NMR spectrum of (*Z*)-verbenone oxime **3** in $CDCl_3$.

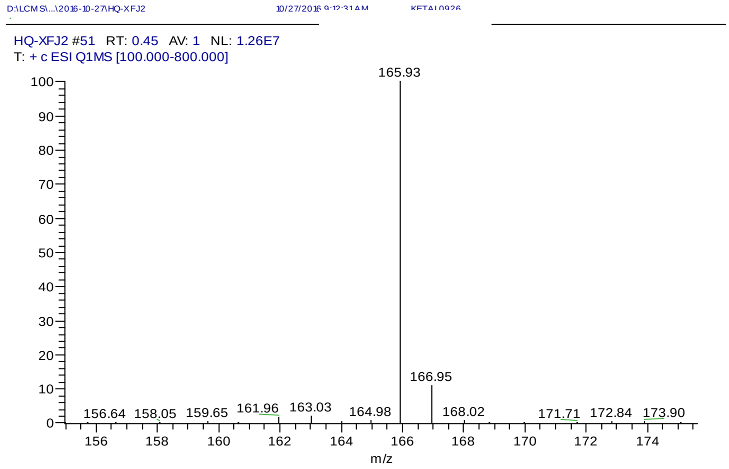

Figure S15. ESI-MS spectrum of (*Z*)-verbenone oxime **3**.

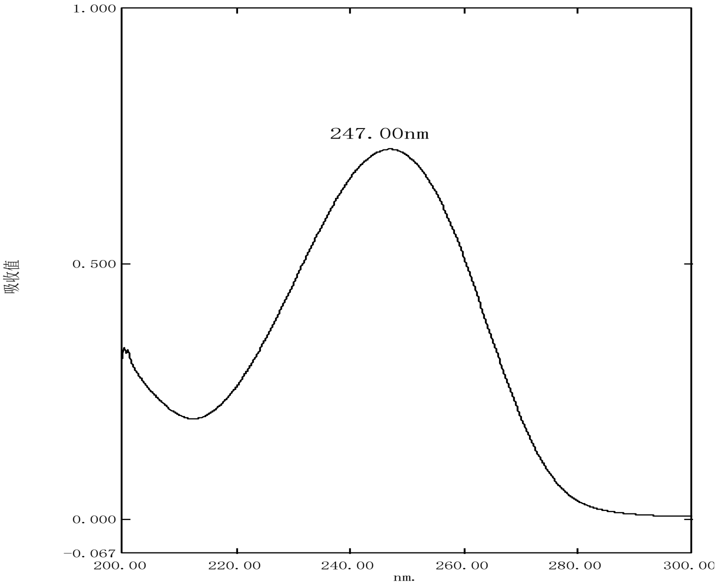

Figure S6. UV-vis spectrum of (*E*)-verbenone oxime **3** in EtOH.

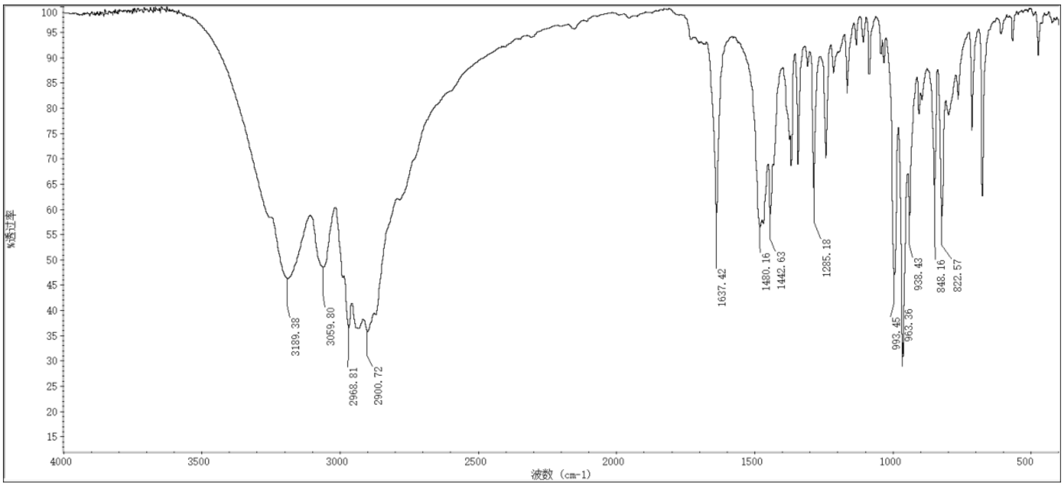

Figure S7. FTIR spectrum of (*E*)-verbenone oxime **3**.

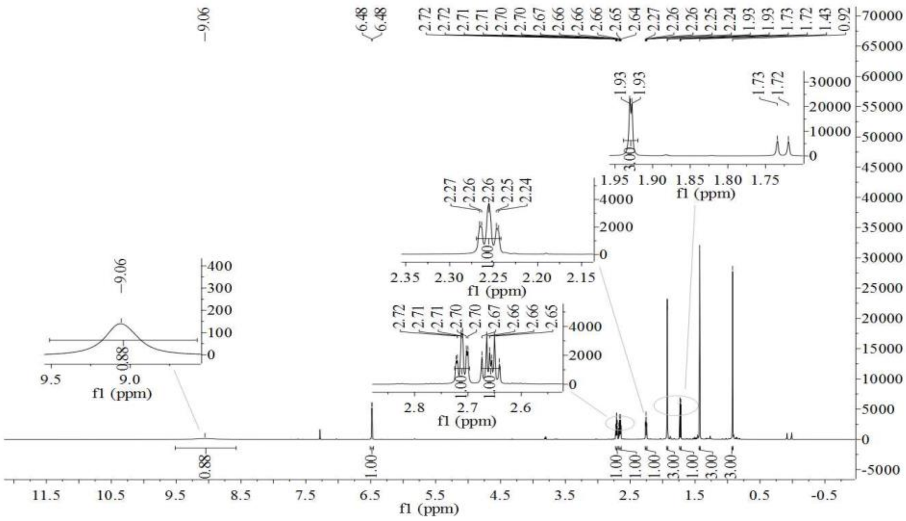

Figure S8. ^{1}H-NMR spectrum of (*E*)-verbenone oxime **3** in $CDCl_3$.

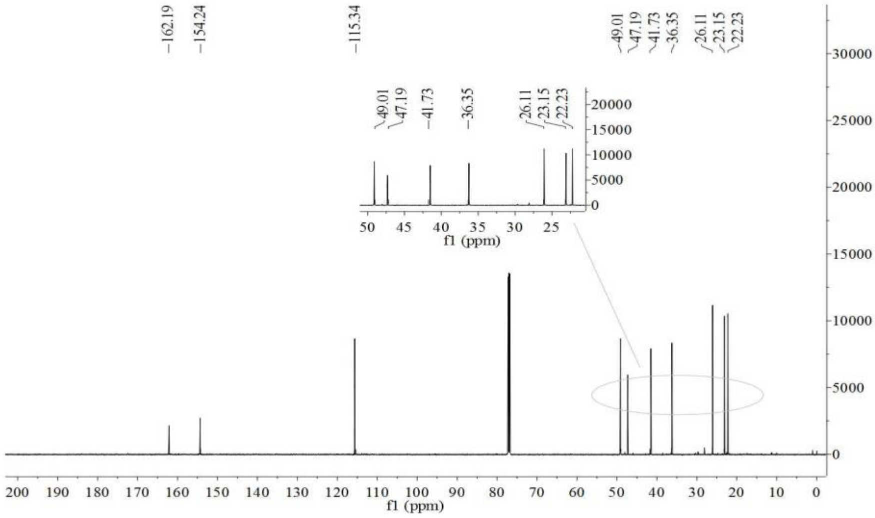

Figure S9. ^{13}C-NMR spectrum of (*E*)-verbenone oxime **3** in $CDCl_3$.

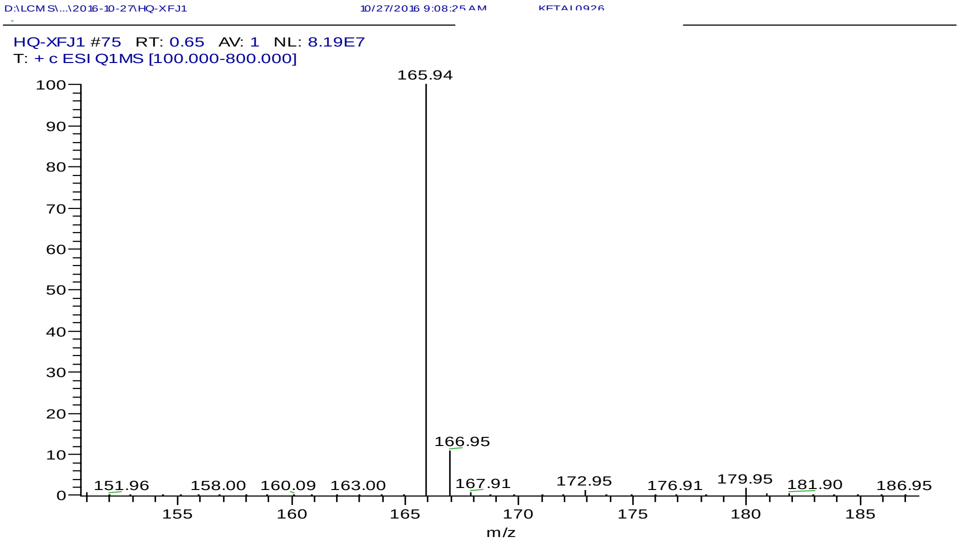

Figure S10. ESI-MS spectrum of (*E*)-verbenone oxime **3**.

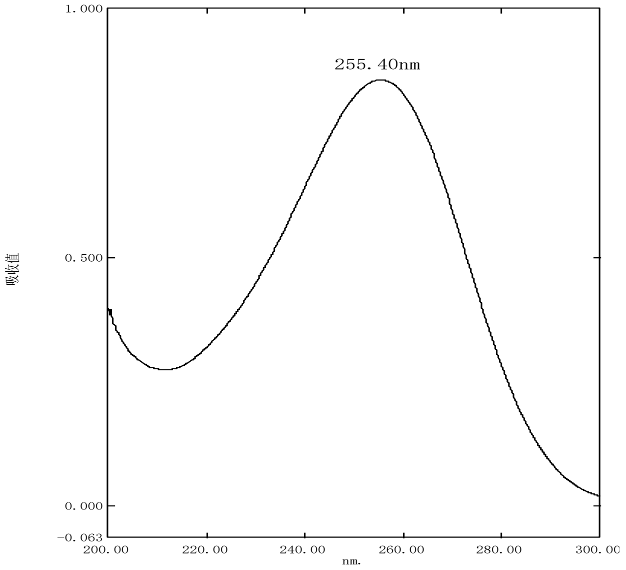

Figure S23. UV-vis spectrum of (*Z*)-verbenone O-*n*-pentanoyl oxime **4a** in EtOH.

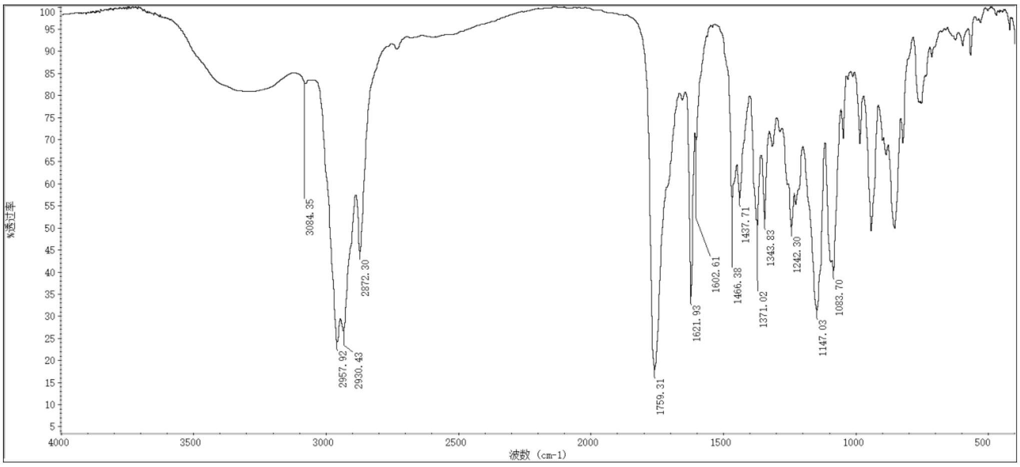

Figure S24. FTIR spectrum of (*Z*)-verbenone O-*n*-pentanoyl oxime **4a**.

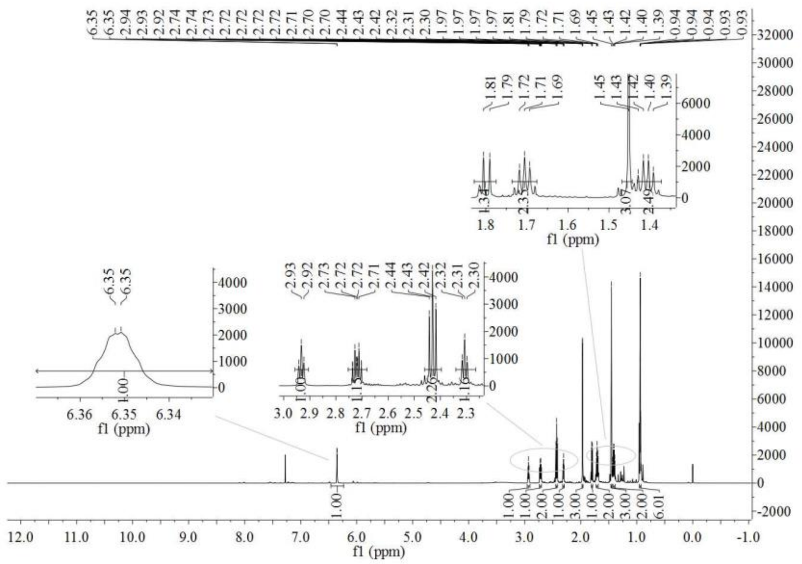

Figure S25. ^{1}H-NMR spectrum of (*Z*)-verbenone O-*n*-pentanoyl oxime **4a** in $CDCl_3$.

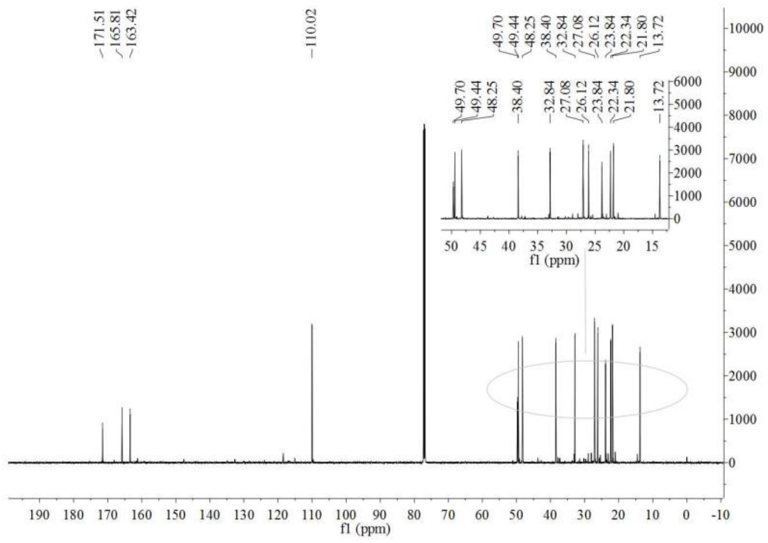

Figure S26. ^{13}C-NMR spectrum of (*Z*)-verbenone O-*n*-pentanoyl oxime in $CDCl_3$.

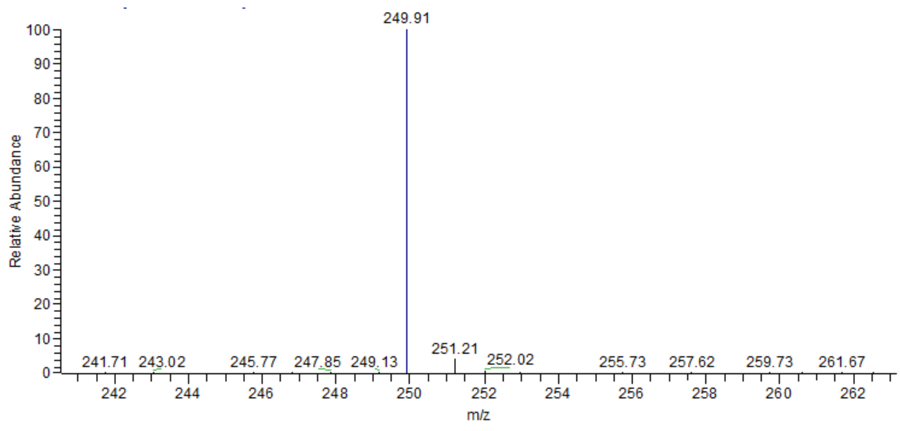

Figure S27. ESI-MS spectrum of (*Z*)-verbenone O-*n*-pentanoyl oxime **4a**.

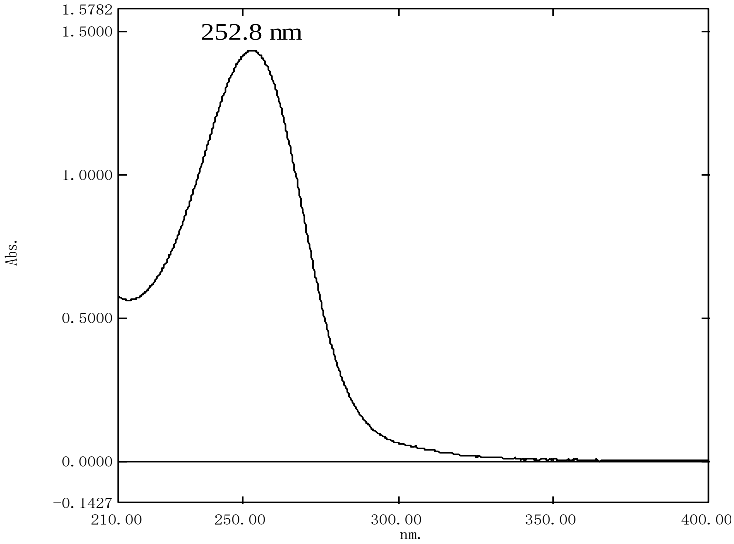

Figure S18. UV-vis spectrum of (*E*)-verbenone O-*n*-pentanoyl oxime **4a** in EtOH.

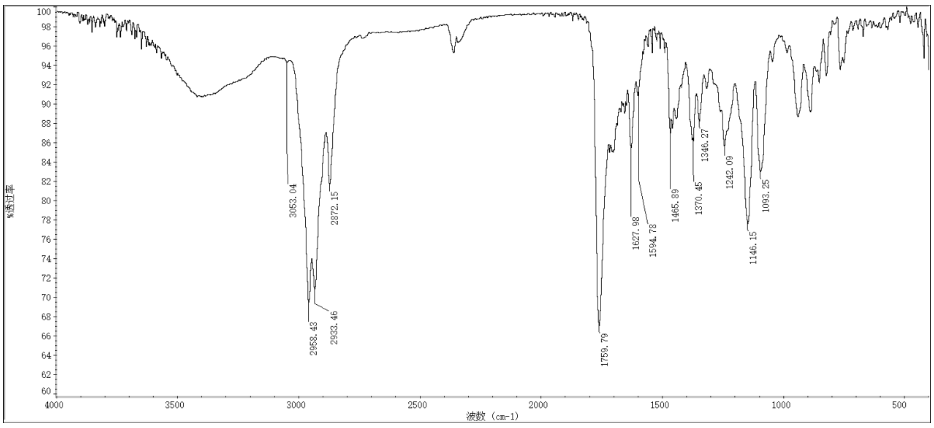

Figure S19. FTIR spectrum of (*E*)-verbenone O-*n*-pentanoyl oxime **4a**.

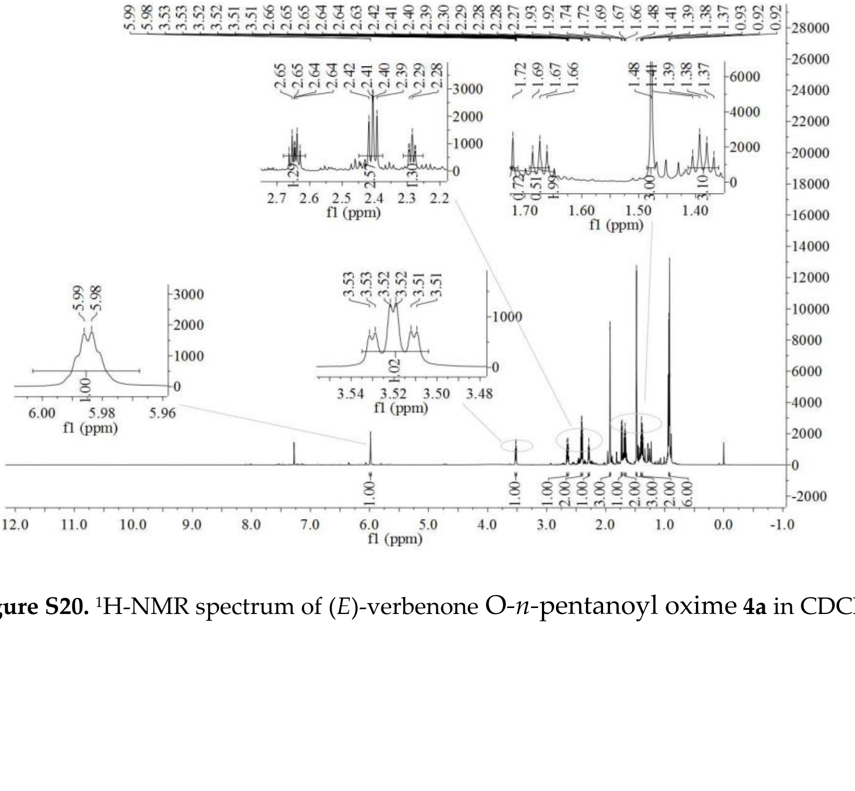

Figure S20. ^{1}H-NMR spectrum of (*E*)-verbenone O-*n*-pentanoyl oxime **4a** in $CDCl_3$.

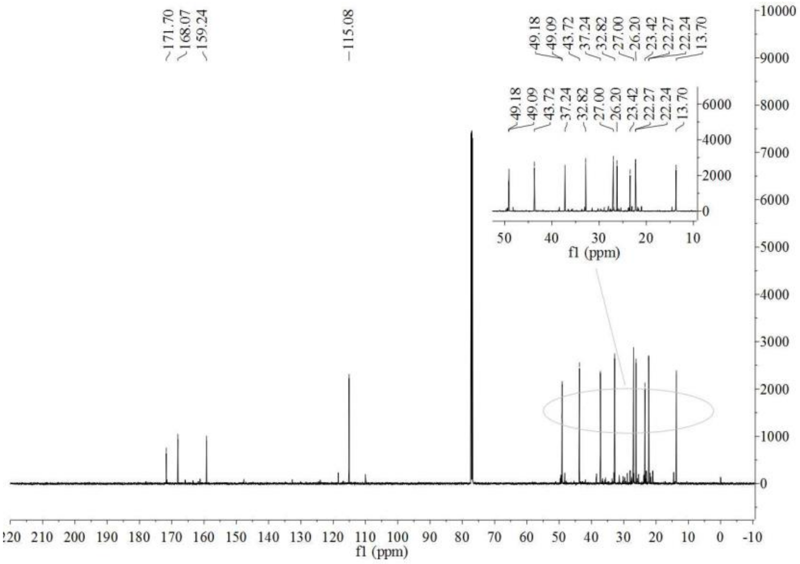

Figure S21. ^{13}C-NMR spectrum of (*E*)-verbenone O-*n*-pentanoyl oxime **4a** in $CDCl_3$.

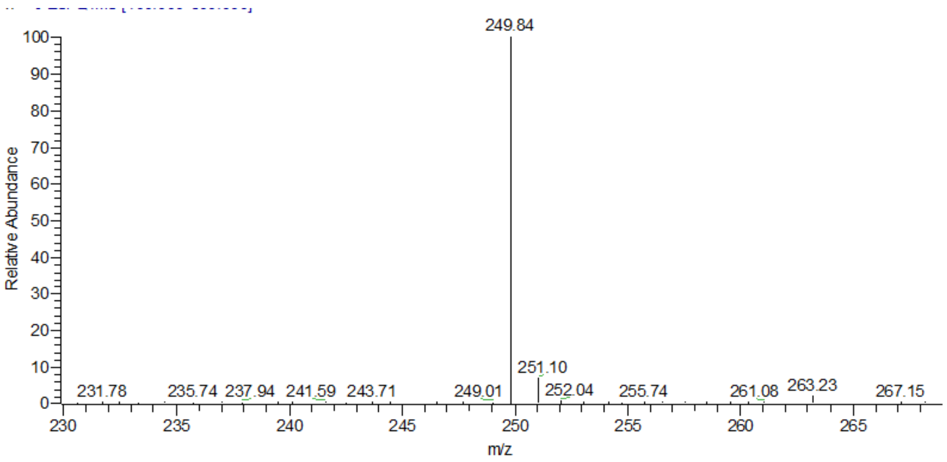

Figure S22. ESI-MS spectrum of (*E*)-verbenone O-*n*-pentanoyl oxime **4a**.

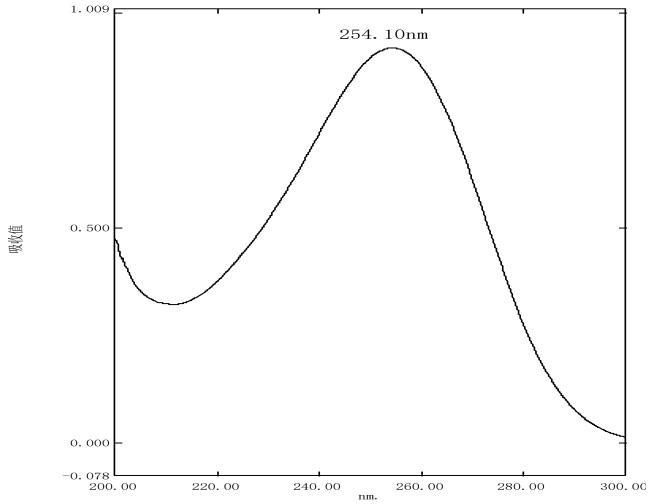

Figure S33. UV-vis spectrum of (*Z*)-verbenone O-*n*-hexanoyl oxime **4b** in EtOH.

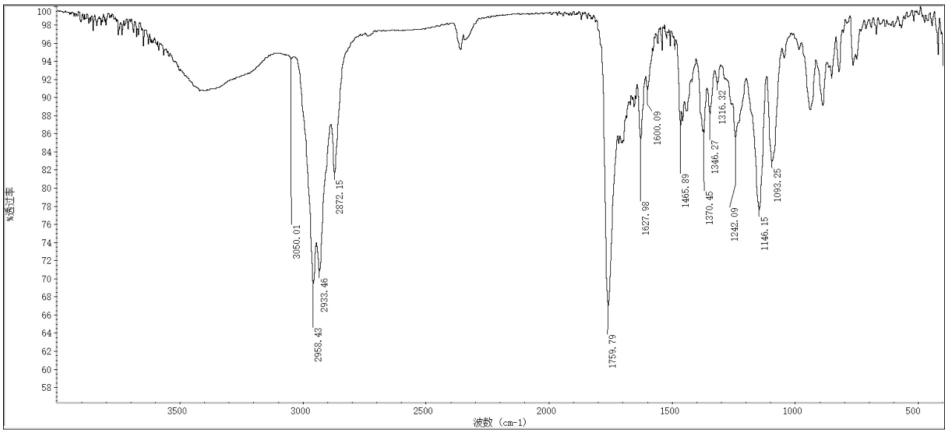

Figure S34. FTIR spectrum of (*Z*)-verbenone O-*n*-hexanoyl oxime **4b**.

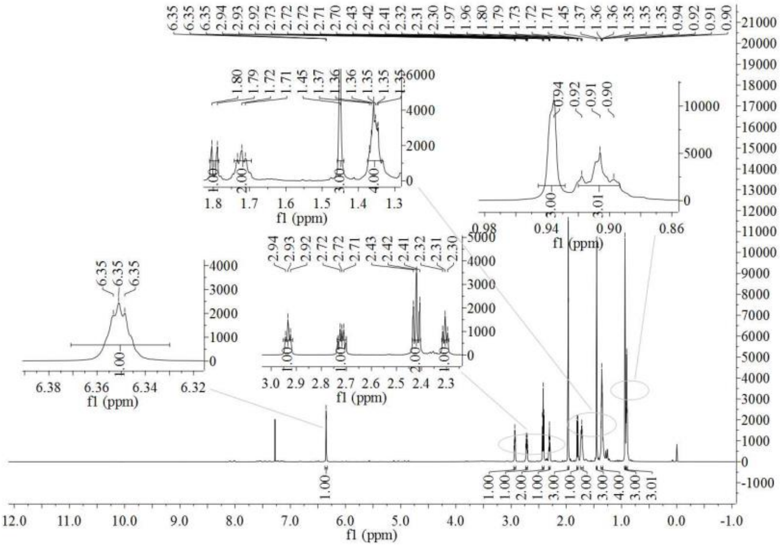

Figure S35. ^{1}H-NMR spectrum of (*Z*)-verbenone O-*n*-hexanoyl oxime **4b** in $CDCl_3$.

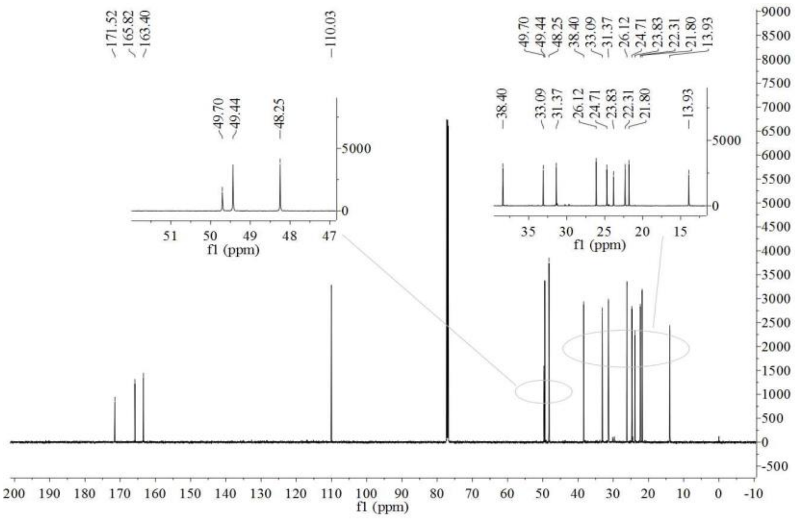

Figure S36. ^{13}C-NMR spectrum of (*Z*)-verbenone O-*n*-hexanoyl oxime **4b** in $CDCl_3$.

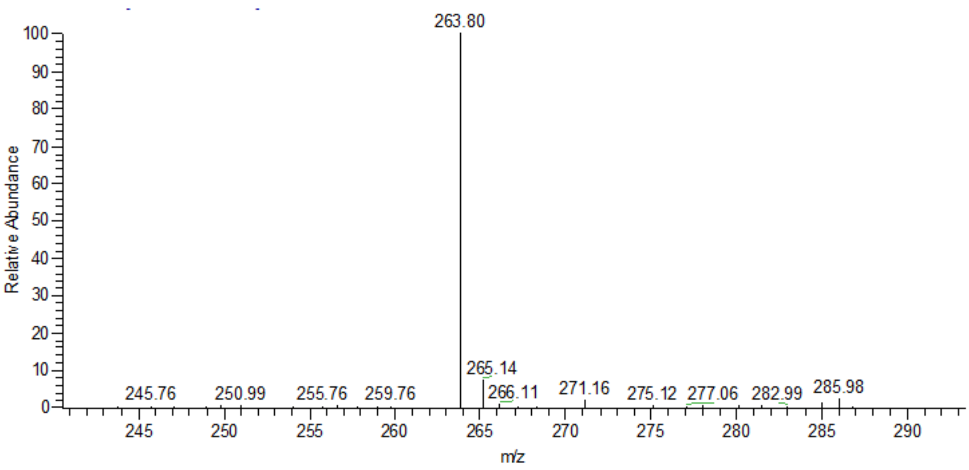

Figure S37. ESI-MS spectrum of (*Z*)-verbenone O-*n*-hexanoyl oxime **4b**.

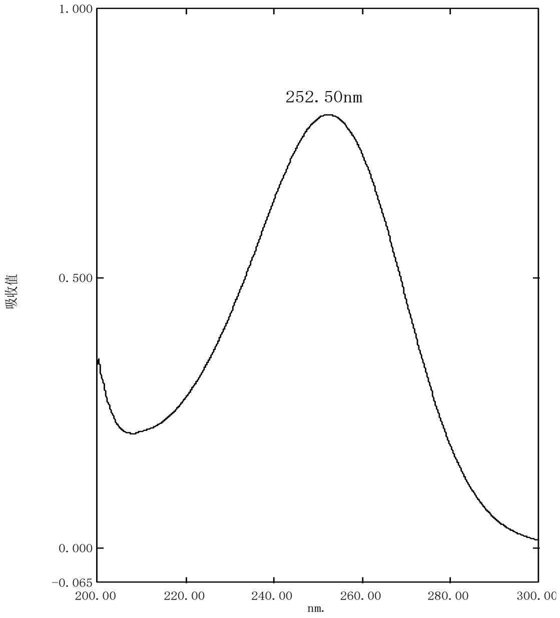

Figure S28. UV-vis spectrum of (*E*)-verbenone O-*n*-hexanoyl oxime **4b** in EtOH.

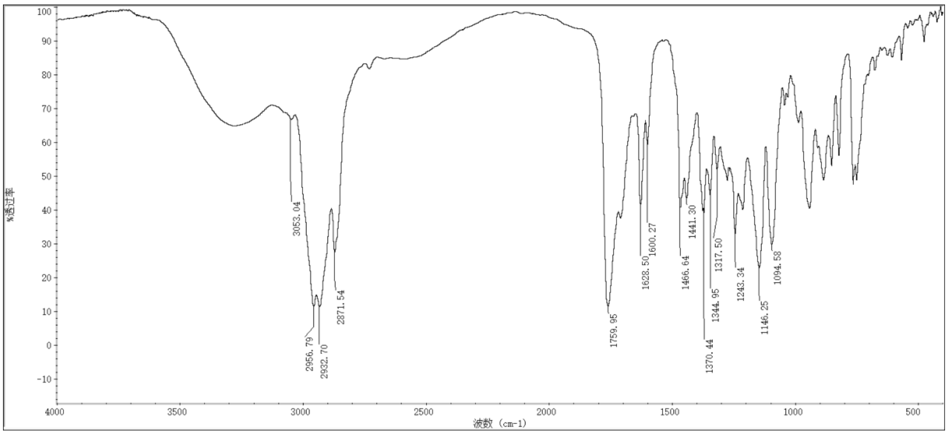

Figure S29. FTIR spectrum of (*E*)-verbenone O-*n*-hexanoyl oxime **4b**.

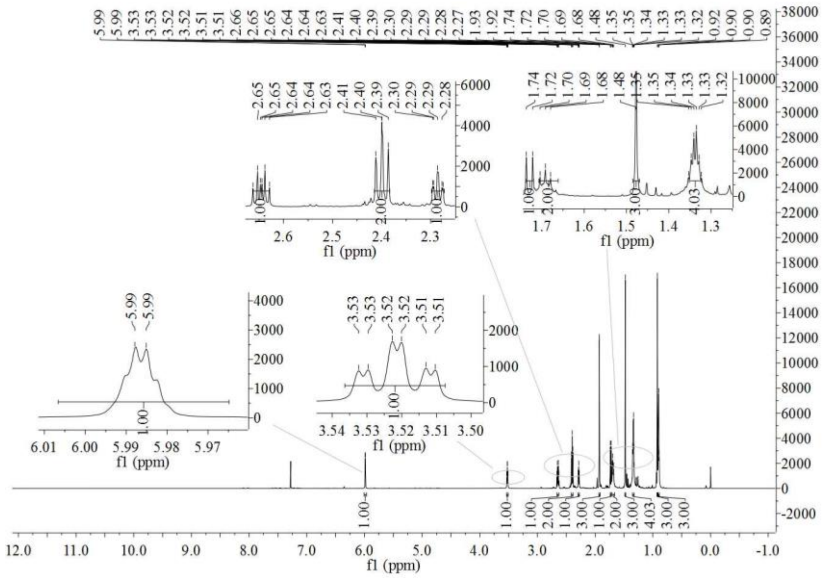

Figure S30. ^{1}H-NMR spectrum of (*E*)-verbenone O-*n*-hexanoyl oxime **4b** in $CDCl_3$.

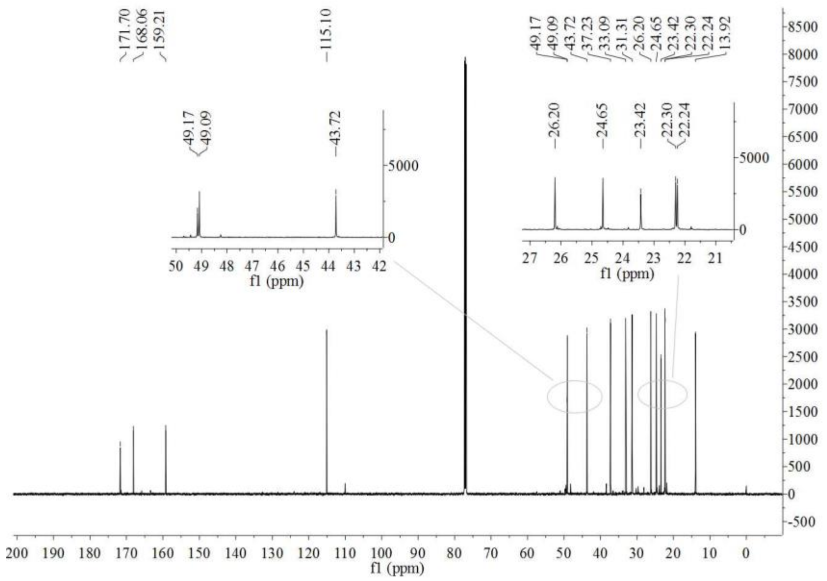

Figure S31. ^{13}C-NMR spectrum of (*E*)-verbenone O-*n*-hexanoyl oxime **4b** in $CDCl_3$.

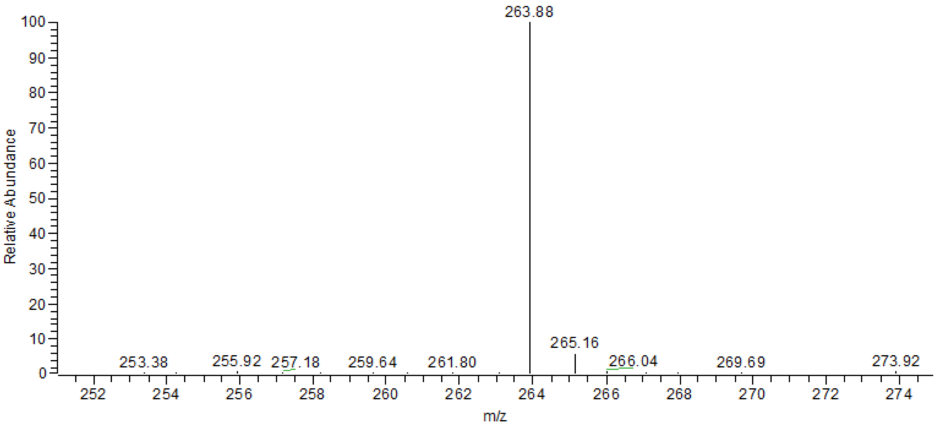

Figure S32. ESI-MS spectrum of (*E*)-verbenone O-*n*-hexanoyl oxime **4b**.

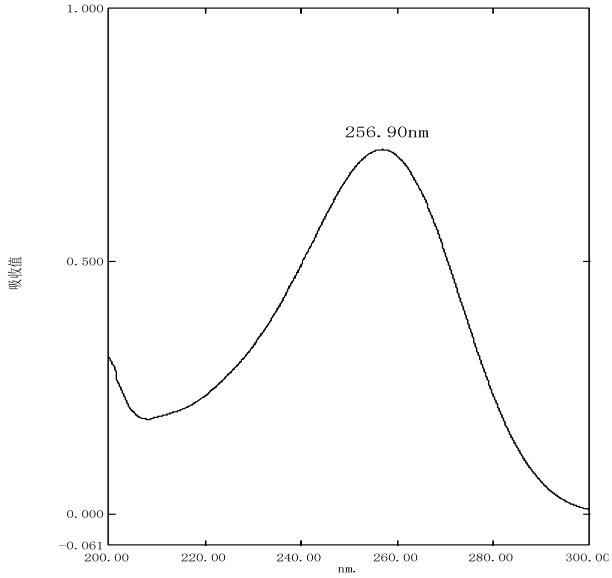

Figure S43. UV-vis spectrum of (*Z*)-verbenone O-cyclopentylcarbonyl oxime **4c** in EtOH.

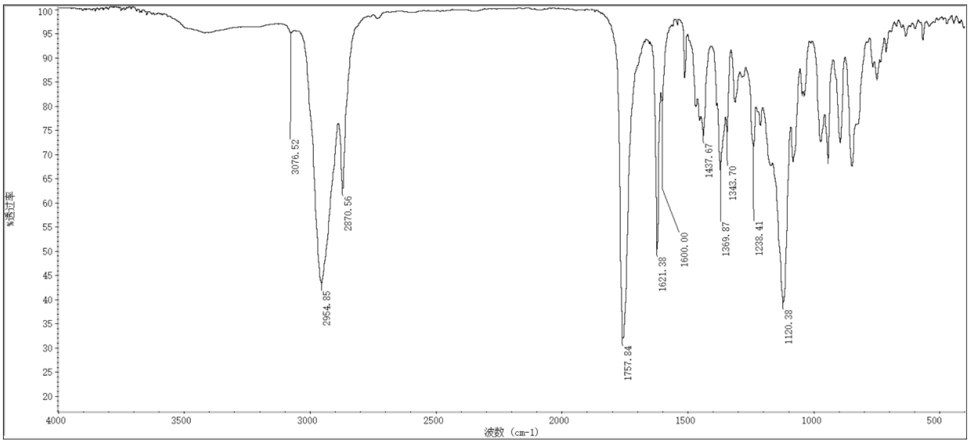

Figure S44. FTIR spectrum of (*Z*)-verbenone O-cyclopentylcarbonyl oxime **4c**.

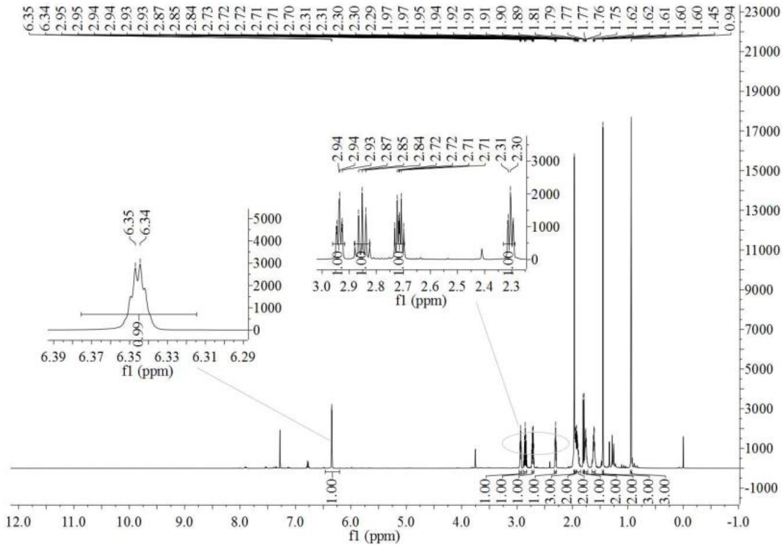

Figure S45. ^{1}H-NMR spectrum of (*Z*)-verbenone O-cyclopentylcarbonyl oxime **4c** in $CDCl_3$.

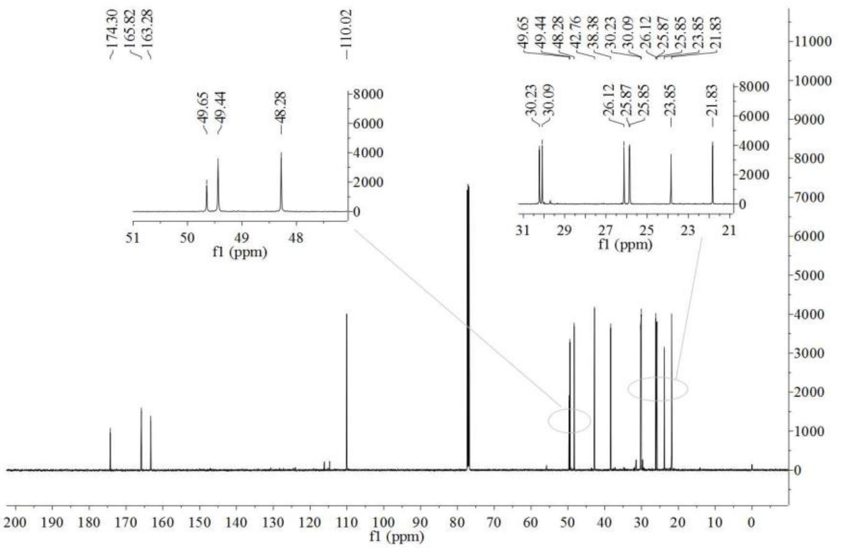

Figure S46. ^{13}C-NMR spectrum of (*Z*)-verbenone O-cyclopentylcarbonyl oxime **4c** in $CDCl_3$.

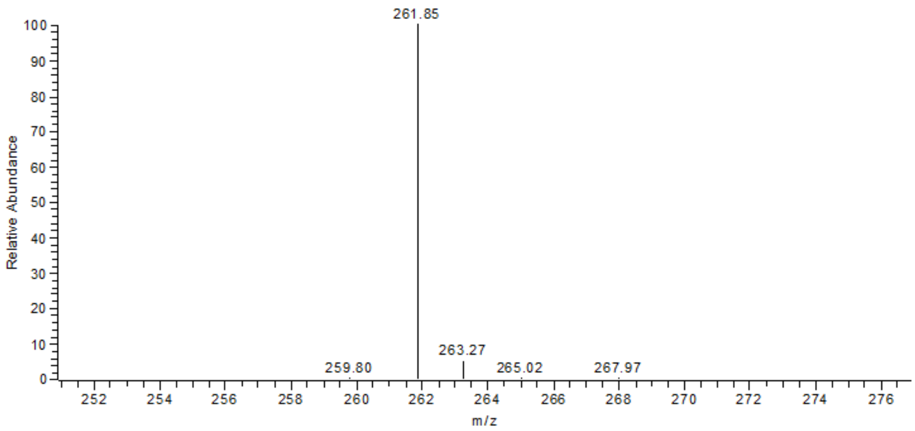

Figure S47. ESI-MS spectrum of (*Z*)-verbenone O-cyclopentylcarbonyl oxime **4c**.

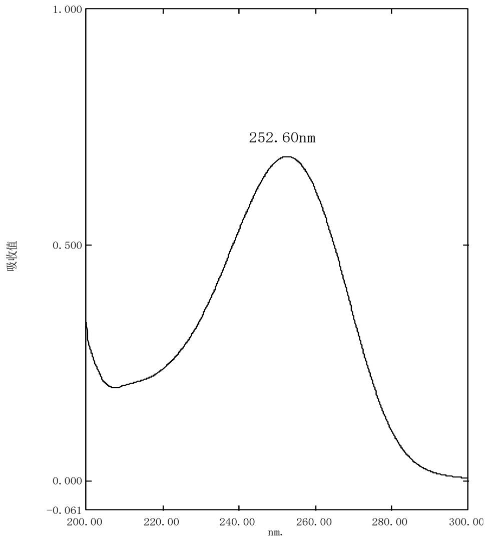

Figure S38. UV-vis spectrum of (*E*)-verbenone O-cyclopentylcarbonyl oxime **4c** in EtOH.

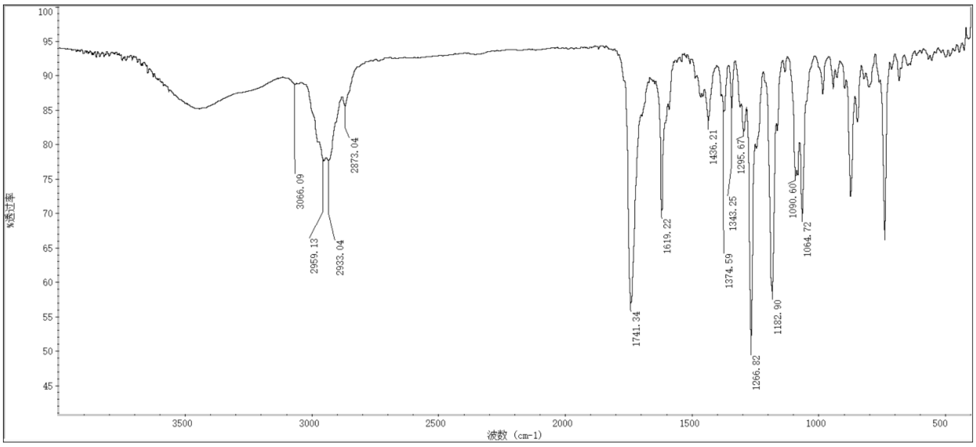

Figure S39. FTIR spectrum of (*E*)-verbenone O-cyclopentylcarbonyl oxime **4c**.

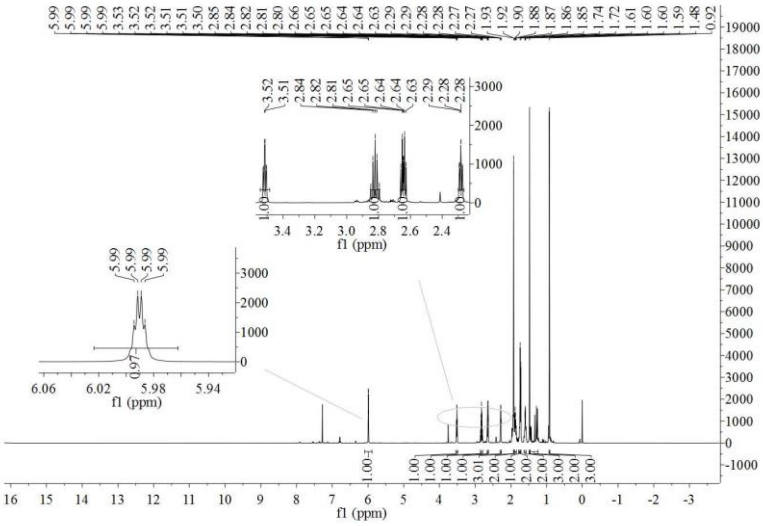

Figure S40. ^{1}H-NMR spectrum of (*E*)-verbenone O-cyclopentylcarbonyl oxime **4c** in $CDCl_3$.

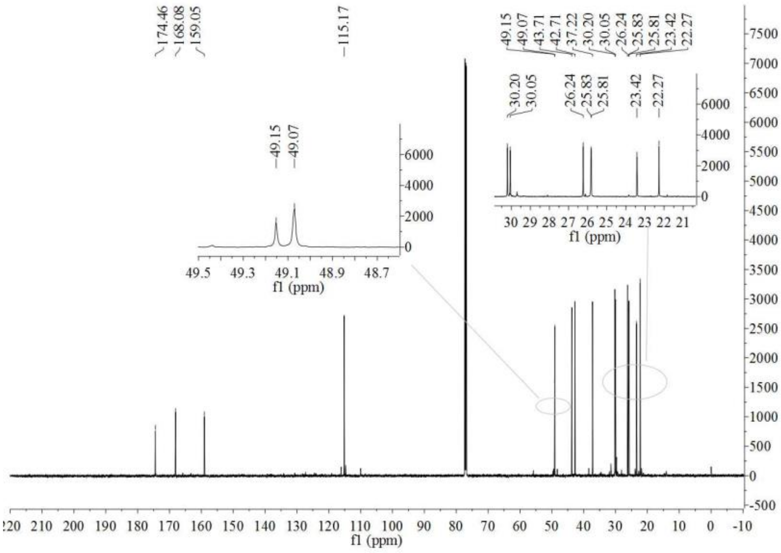

Figure S41. ^{13}C-NMR spectrum of (*E*)-verbenone O-cyclopentylcarbonyl oxime **4c** in $CDCl_3$.

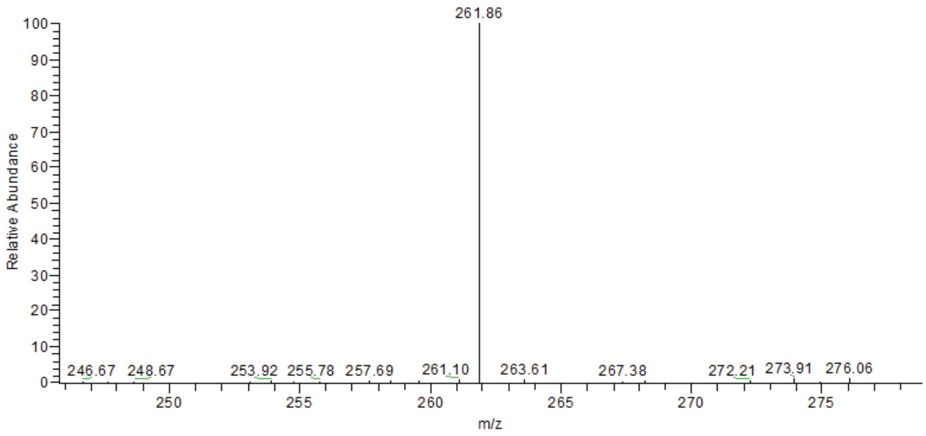

Figure S42. ESI-MS spectrum of (*E*)-verbenone O-cyclopentylcarbonyl oxime **4c**.

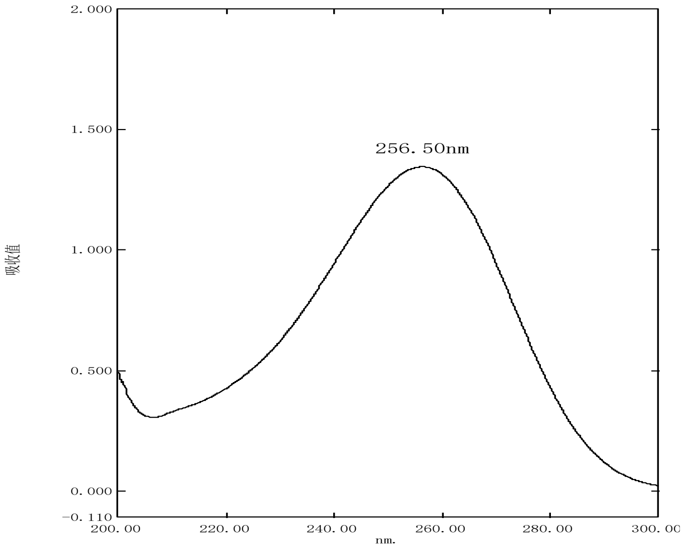

Figure S53. UV-vis spectrum of (*Z)*-verbenone O-cyclohexylcarbonyl oxime **4d** in EtOH.

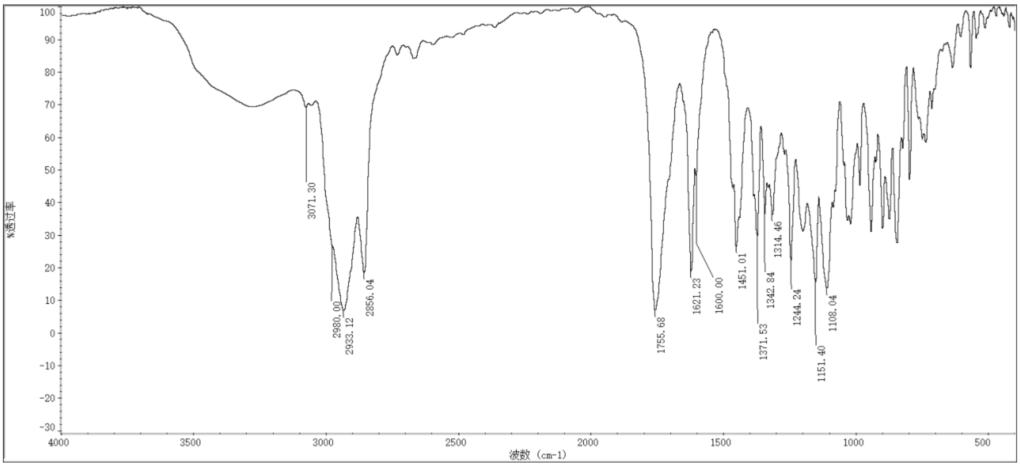

Figure S54. FTIR spectrum of (*Z)*-verbenone O-cyclohexylcarbonyl oxime **4d**.

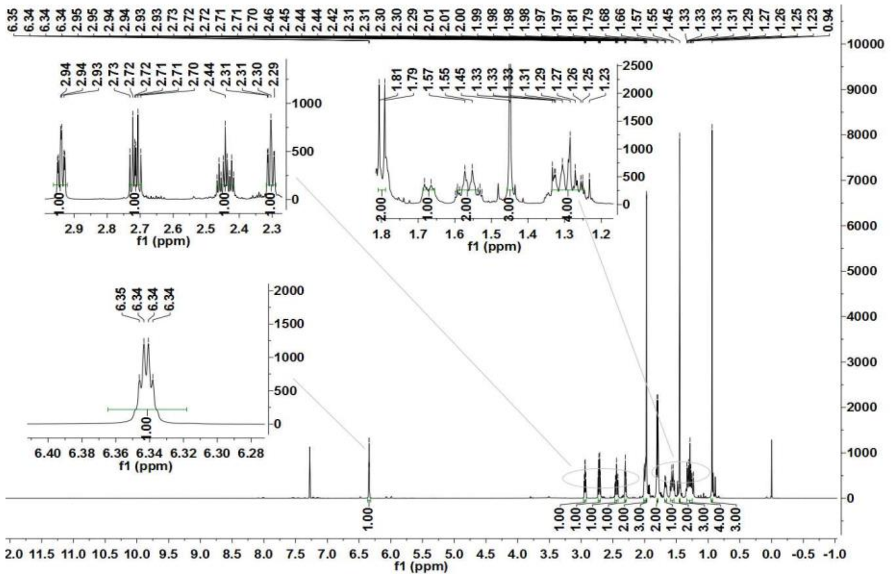

Figure S55. ^{1}H-NMR spectrum of (*Z*)-verbenone O-cyclohexylcarbonyl oxime **4d** in $CDCl_3$.

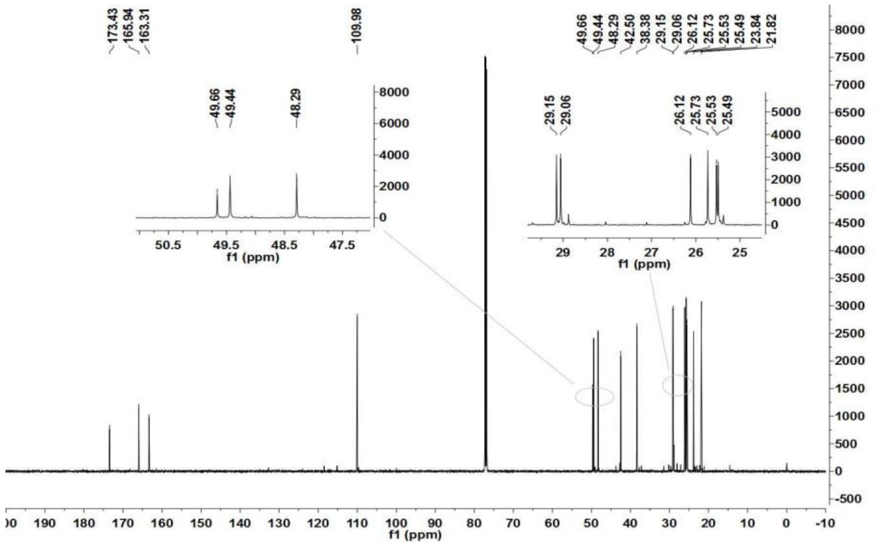

Figure S56. ^{13}C-NMR spectrum of (*Z*)-verbenone O-cyclohexylcarbonyl oxime **4d** in $CDCl_3$.

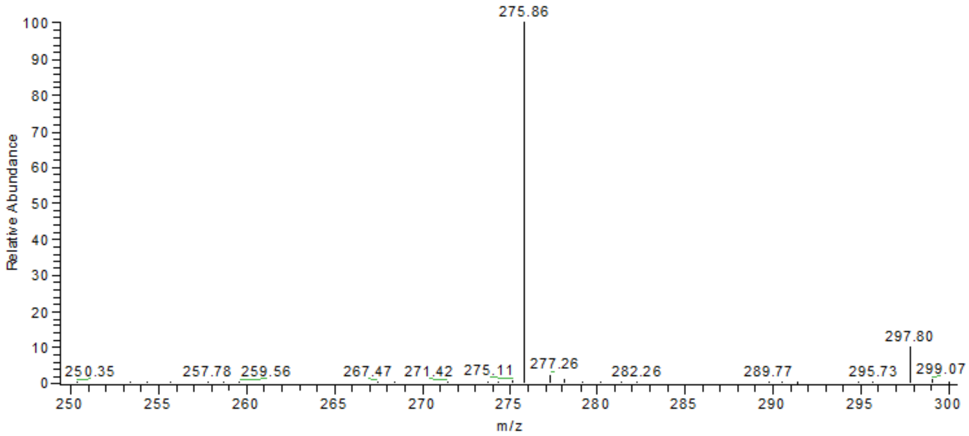

Figure S57. ESI-MS spectrum of (*Z*)-verbenone O-cyclohexylcarbonyl oxime **4d**.

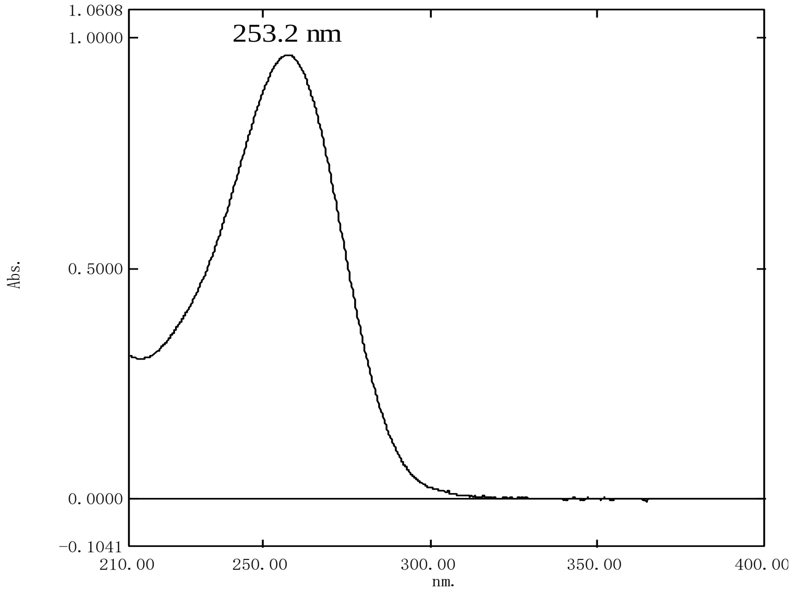

Figure S48. UV-vis spectrum of (*E)*-verbenone O-cyclohexylcarbonyl oxime **4d** in EtOH.

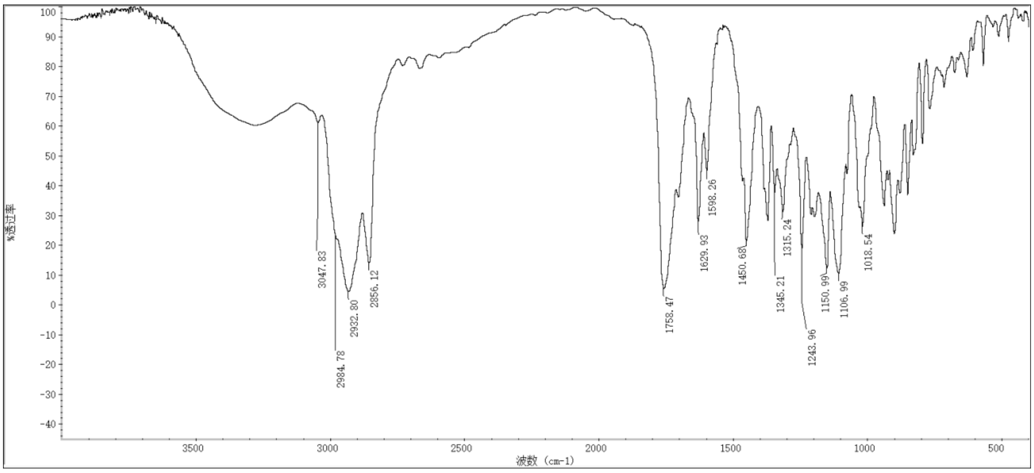

Figure S49. FTIR spectrum of (*E)*-verbenone O-cyclohexylcarbonyl oxime **4d**.

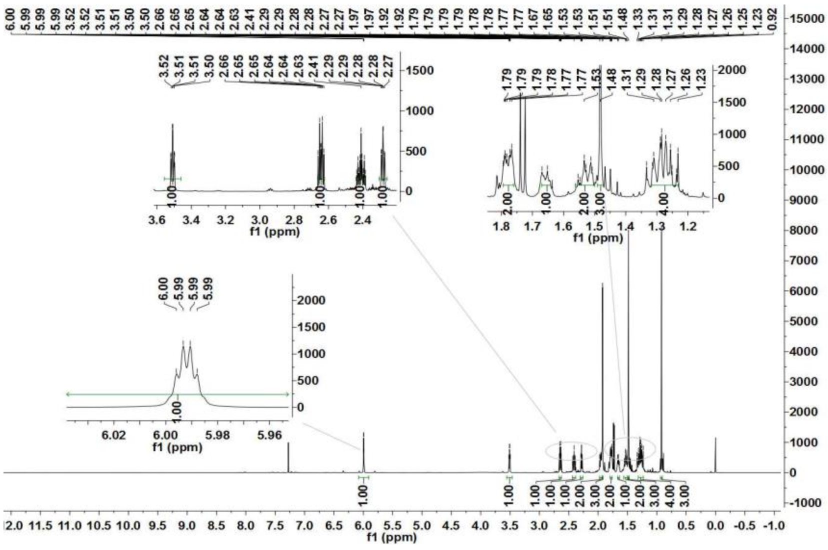

Figure S50. ¹H-NMR spectrum of (*E)*-verbenone O-cyclohexylcarbonyl oxime **4d** in $CDCl_3$.

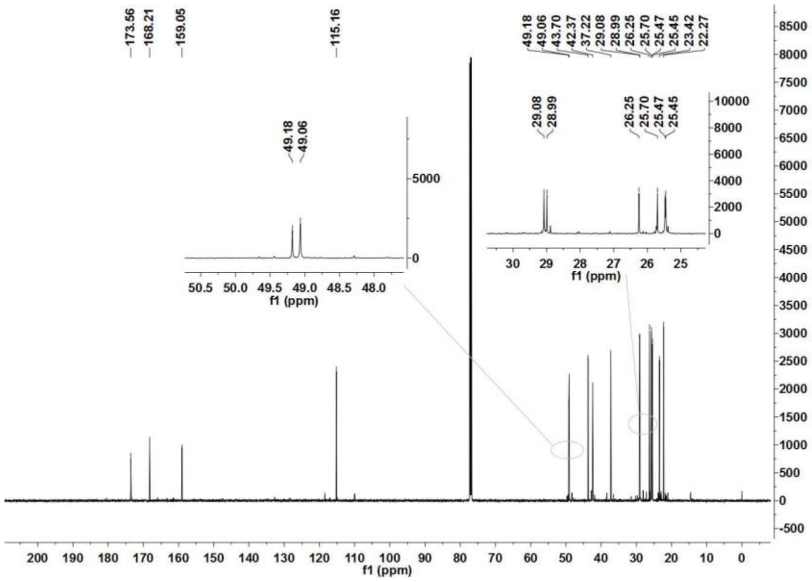

Figure S51. ^{13}C-NMR spectrum of (*E*)-verbenone O-cyclohexylcarbonyl oxime **4d** in $CDCl_3$.

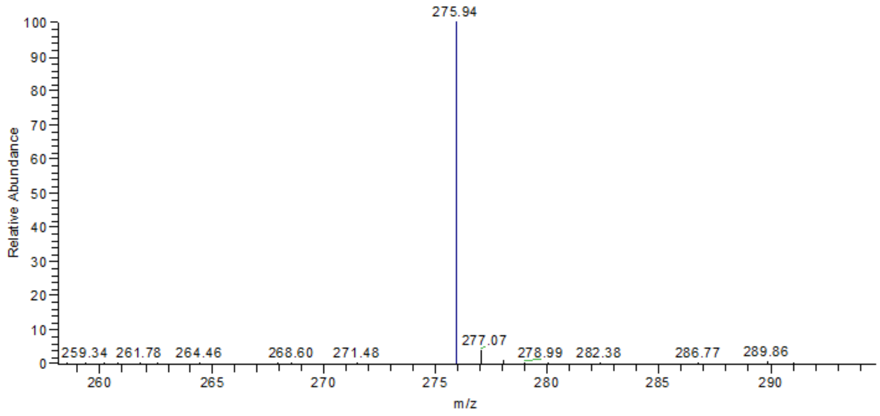

Figure S52. ESI-MS spectrum of (*E*)-verbenone O-cyclohexylcarbonyl oxime **4d**.

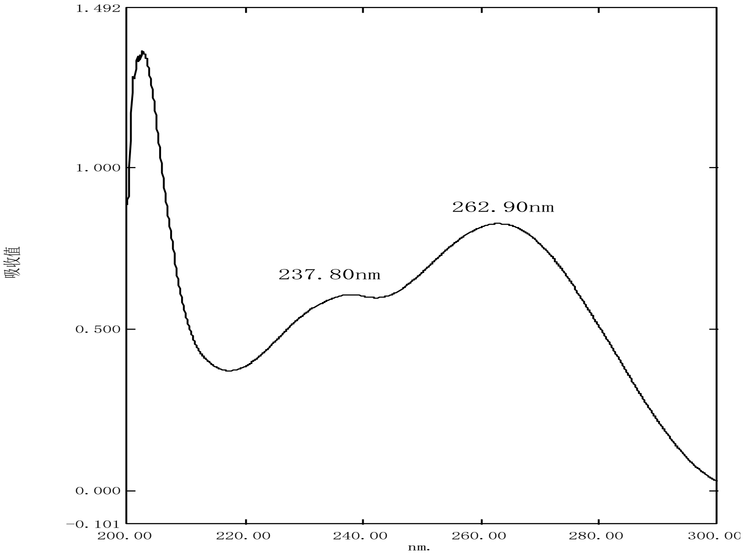

Figure S63. UV-vis spectrum of (*Z*)-verbenone O-(2′-methylbenzoyl) oxime **4e** in EtOH.

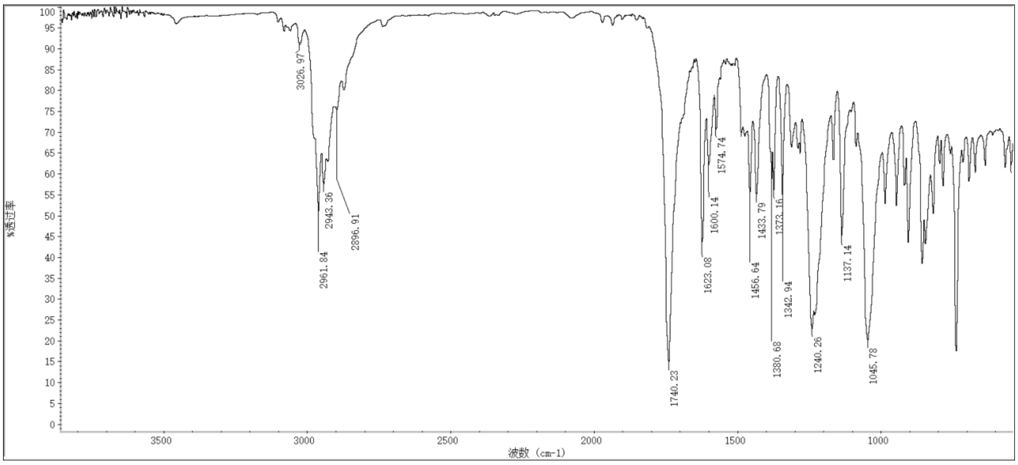

Figure S64. FTIR spectrum of (*Z)*-verbenone O-(2′-methylbenzoyl) oxime **4e**.

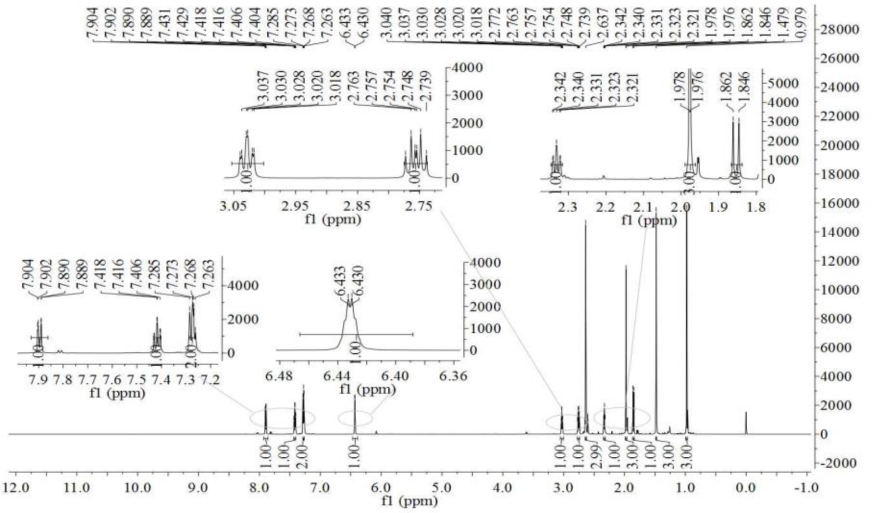

Figure S65. ¹H-NMR spectrum of (*Z)*-verbenone O-(2′-methylbenzoyl) oxime **4e** in $CDCl_3$.

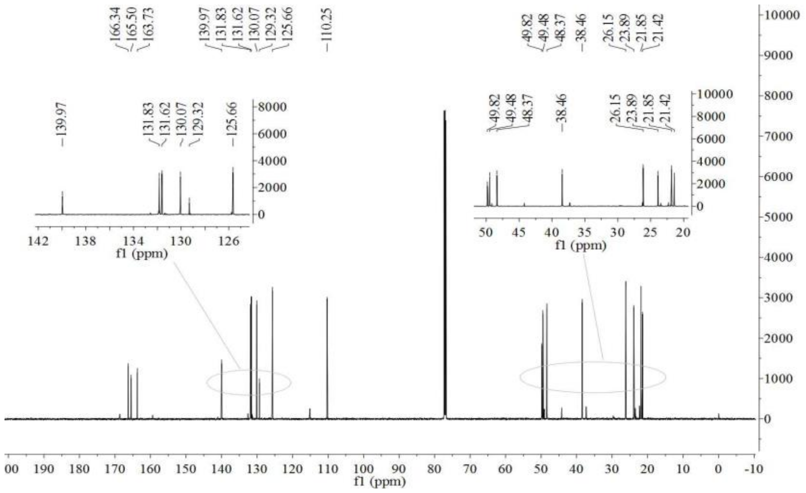

Figure S66. ¹³C-NMR spectrum of (*Z)*-verbenone O-(2′-methylbenzoyl) oxime **4e** in $CDCl_3$.

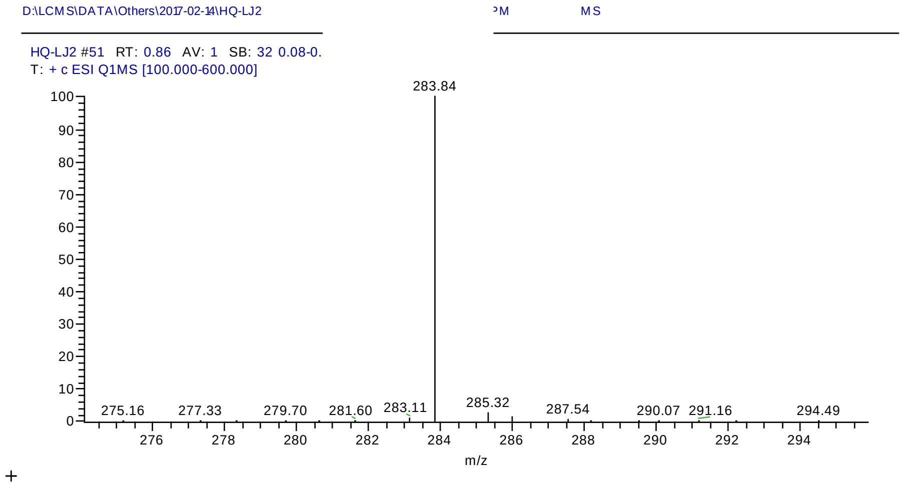

Figure S67. ESI-MS spectrum of (*Z*)-verbenone O-(2′-methylbenzoyl) oxime **4e**.

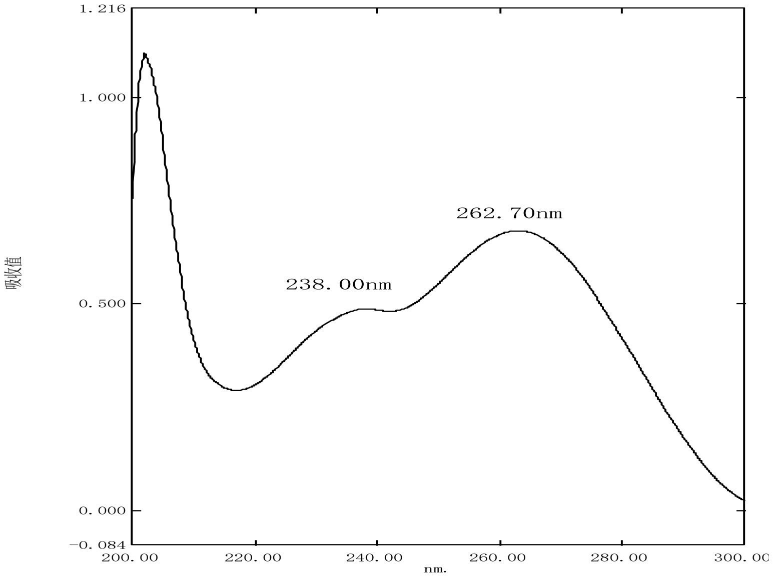

Figure S58. UV-vis spectrum of (*E*)-verbenone O-(2′-methylbenzoyl) oxime **4e** in EtOH.

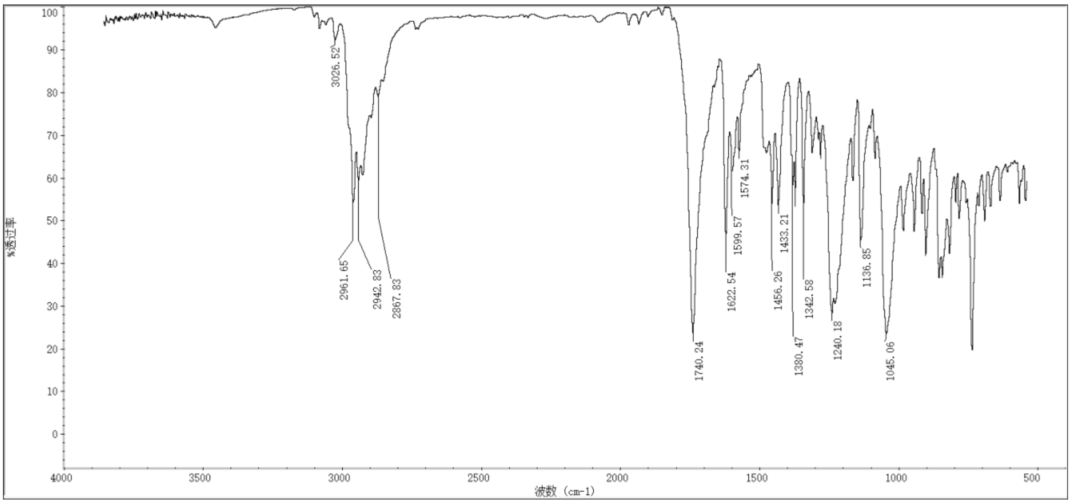

Figure S59. FTIR spectrum of (*E*)-verbenone O-(2′-methylbenzoyl) oxime **4e**.

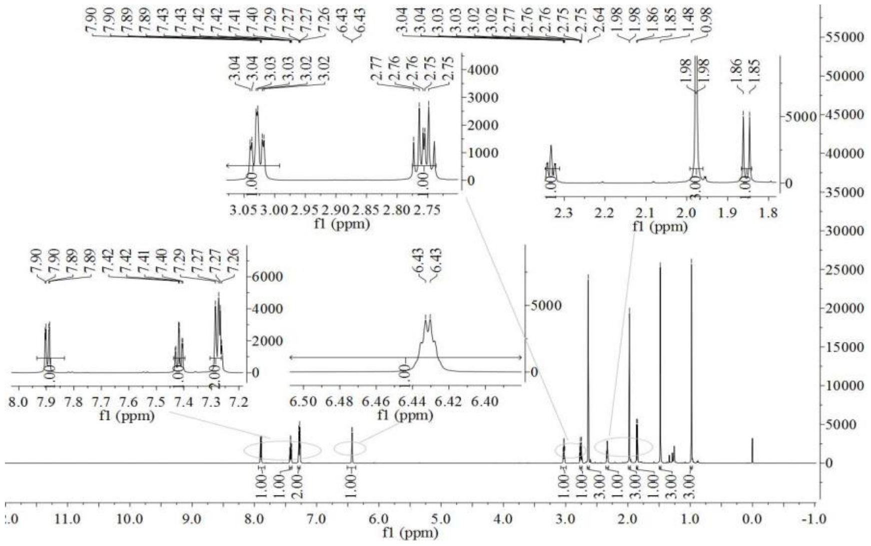

Figure S60. ^{1}H-NMR spectrum of (*E*)-verbenone O-(2′-methylbenzoyl) oxime **4e** in $CDCl_3$.

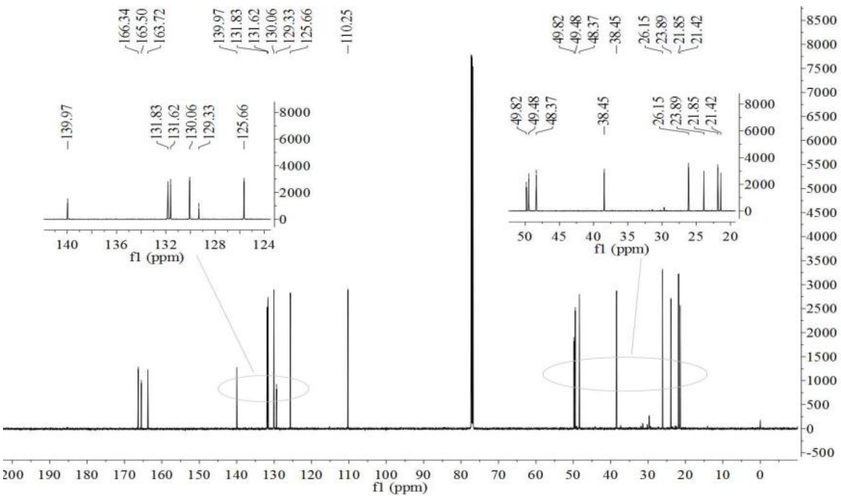

Figure S61. ^{13}C-NMR spectrum of (*E*)-verbenone O-(2′-methylbenzoyl) oxime **4e** in $CDCl_3$.

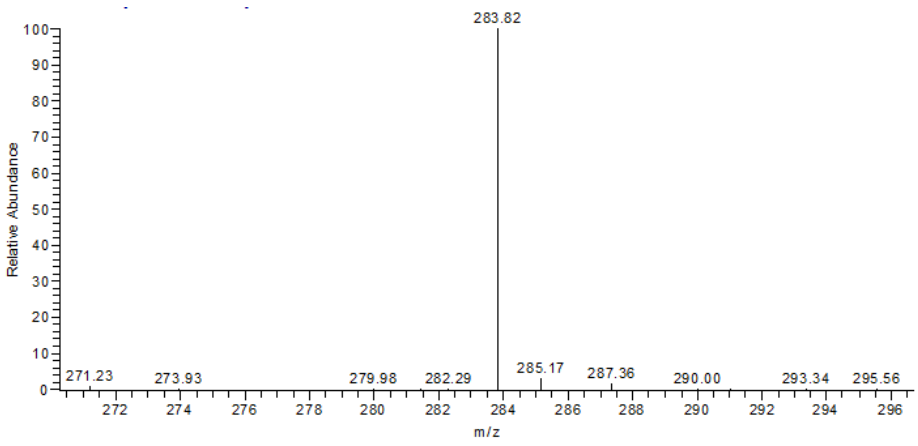

Figure S62. ESI-MS spectrum of (*E*)-verbenone O-(2′-methylbenzoyl) oxime **4e**.

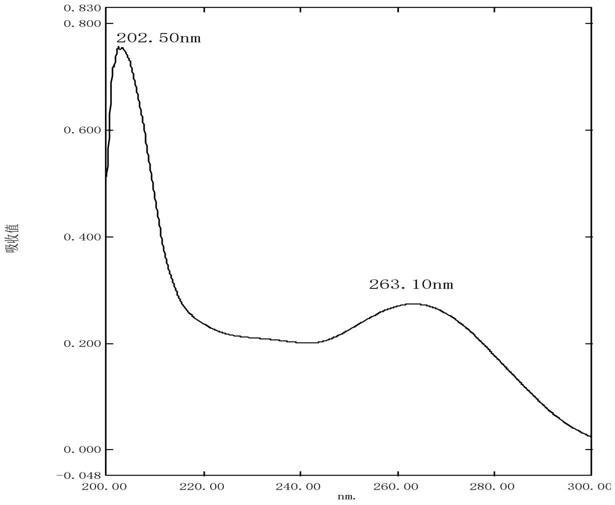

Figure S73. UV-vis spectrum of (*Z*)-verbenone O-(2′-chlorobenzoyl) oxime **4f** in EtOH.

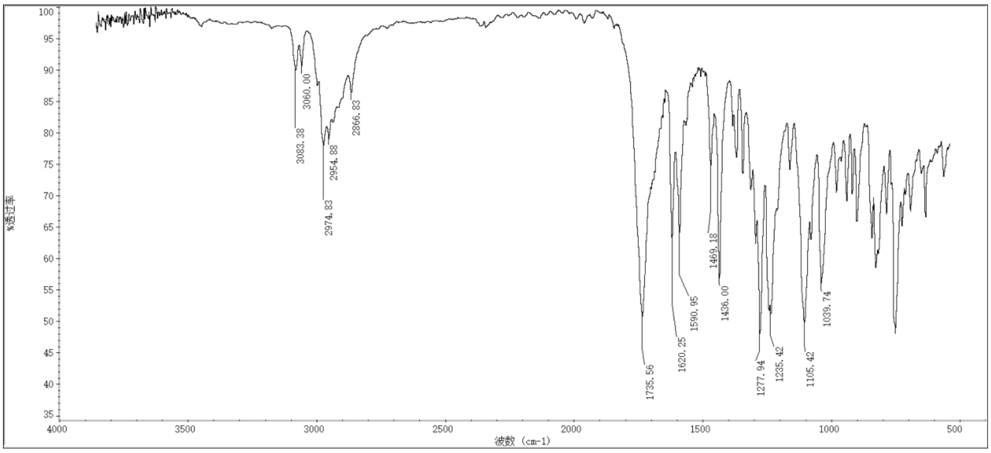

Figure S74. FTIR spectrum of (*Z*)-verbenone O-(2′-chlorobenzoyl) oxime **4f**.

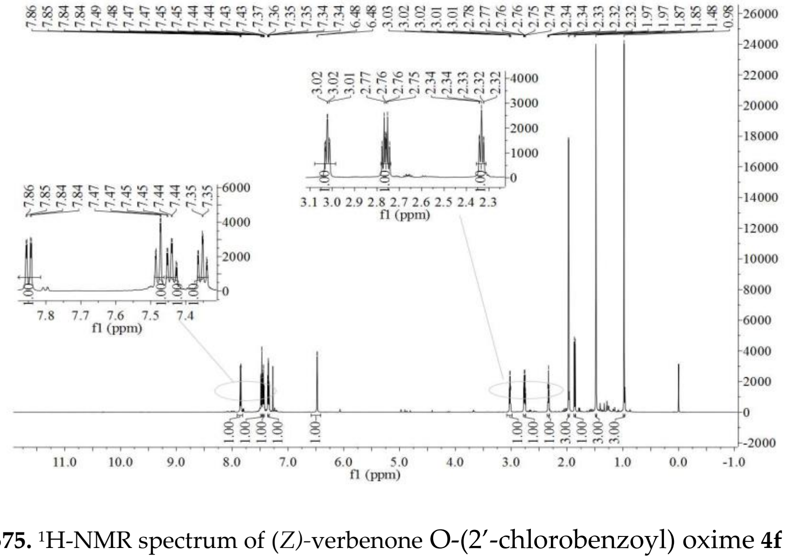

Figure S75. ^{1}H-NMR spectrum of (*Z*)-verbenone O-(2′-chlorobenzoyl) oxime **4f** in $CDCl_3$.

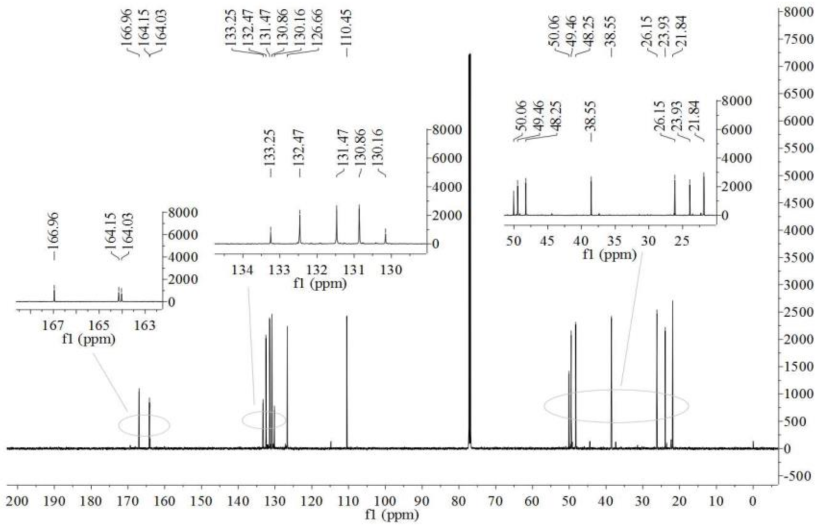

Figure S76. ^{13}C-NMR spectrum of (*Z*)-verbenone O-(2′-chlorobenzoyl) oxime in $CDCl_3$.

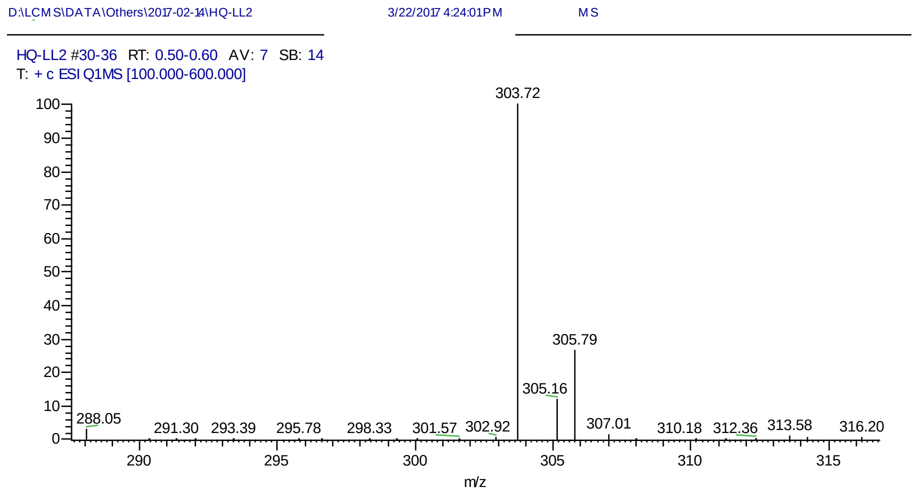

Figure S77. ESI-MS spectrum of (*Z*)-verbenone O-(2′-chlorobenzoyl) oxime **4f**.

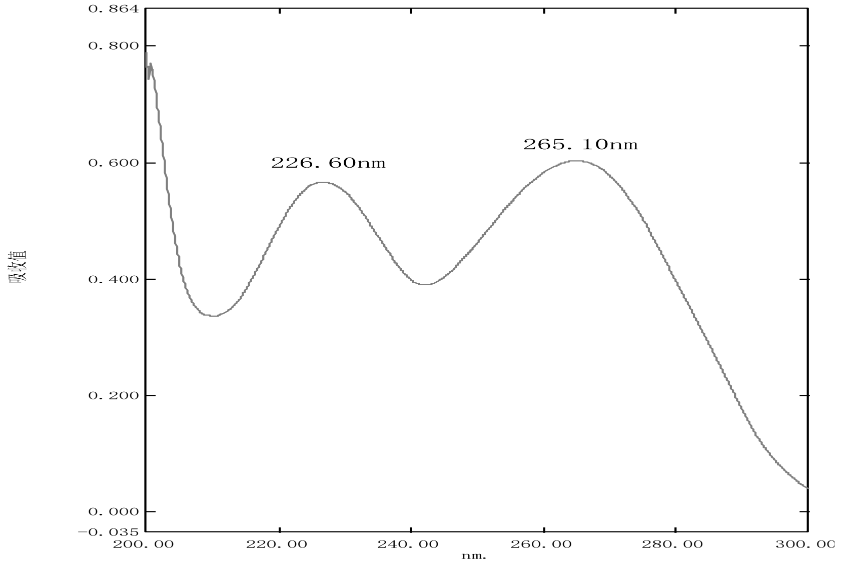

Figure S83. UV-vis spectrum of (*Z*)-verbenone O-(2′-fluorobenzoyl) oxime **4g** in EtOH.

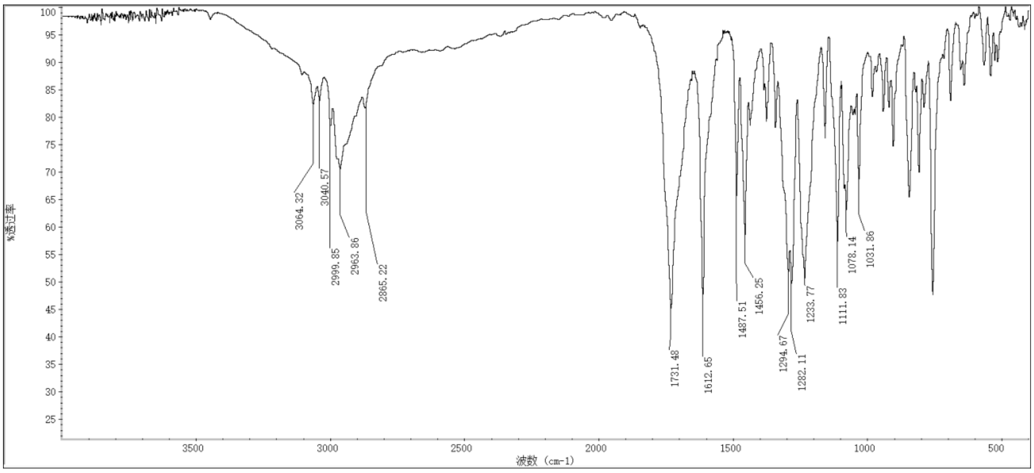

Figure S84. FTIR spectrum of (*Z*)-verbenone O-(2′-fluorobenzoyl) oxime **4g**.

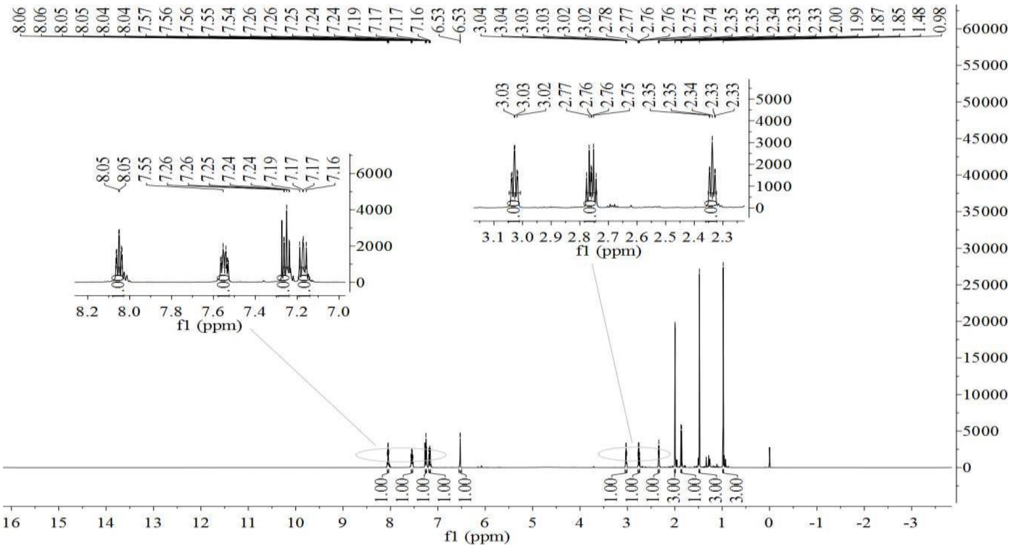

Figure S85. ^{1}H-NMR spectrum of (*Z*)-verbenone O-(2′-fluorobenzoyl) oxime **4g** in $CDCl_3$.

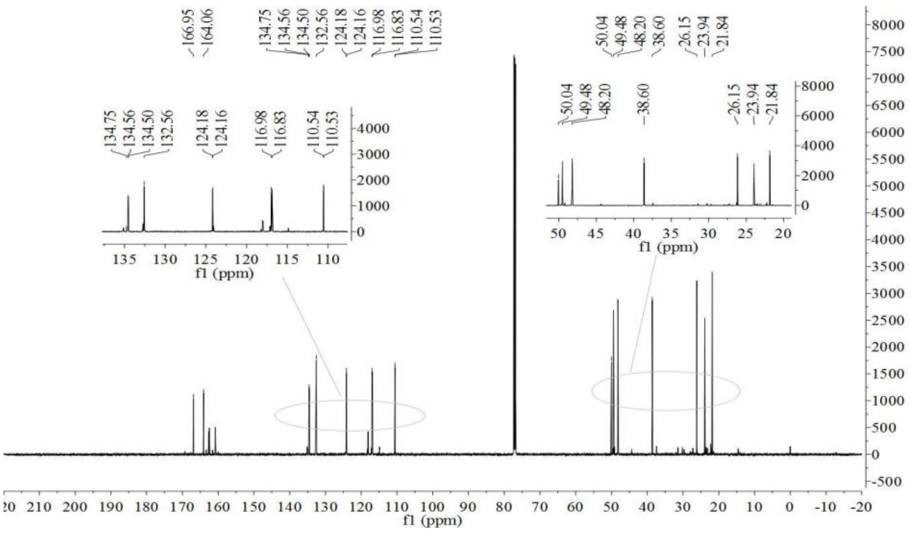

Figure S86. ^{13}C-NMR spectrum of (*Z*)-verbenone O-(2′-fluorobenzoyl) oxime **4g** in $CDCl_3$.

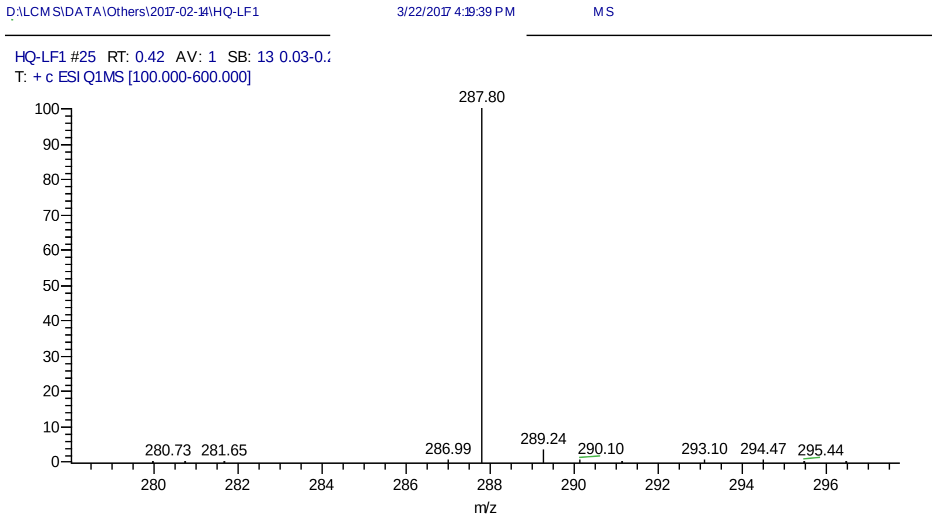

Figure S87. ESI-MS spectrum of (*Z*)-verbenone O-(2′-fluorobenzoyl) oxime **4g**.

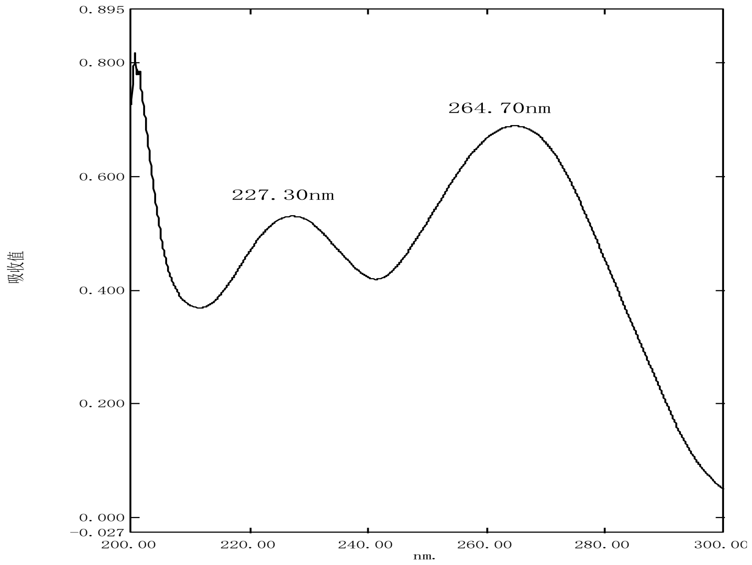

Figure S78. UV-vis spectrum of (*E*)-verbenone O-(2′-fluorobenzoyl) oxime **4g** in EtOH.

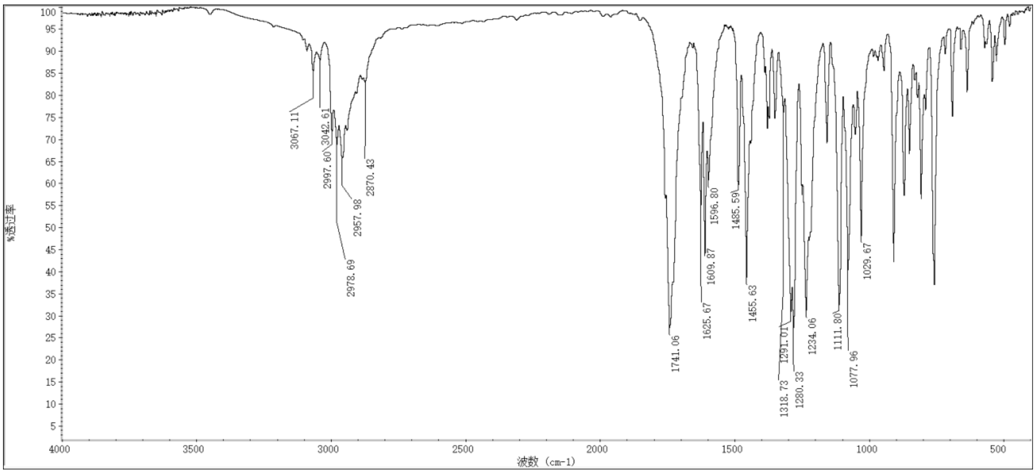

Figure S79. FTIR spectrum of (*E*)-verbenone O-(2′-fluorobenzoyl) oxime **4g**.

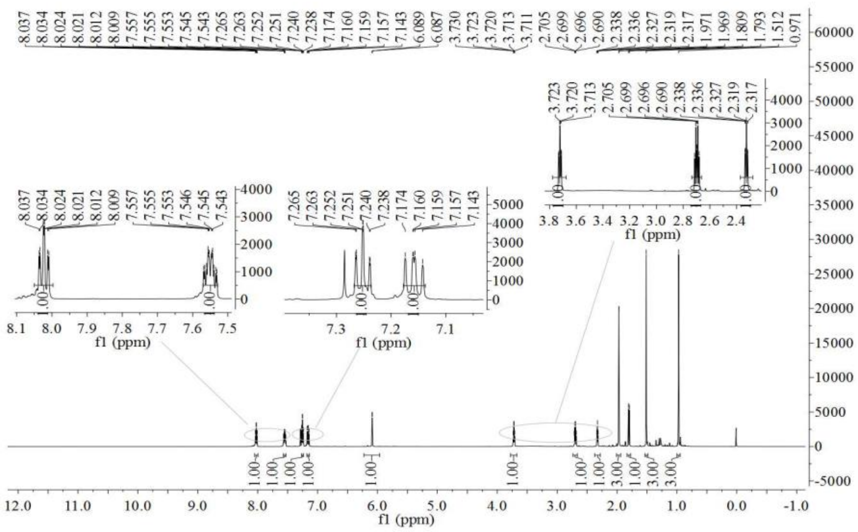

Figure S80. ^{1}H-NMR spectrum of (*E*)-verbenone O-(2′-fluorobenzoyl) oxime **4g** in $CDCl_3$.

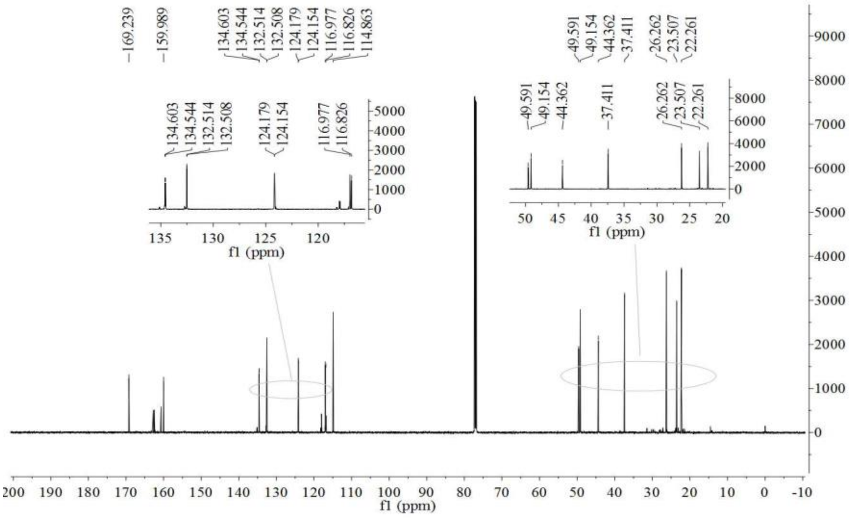

Figure S81. **[13]**C-NMR spectrum of (*E*)-verbenone O-(2′-fluorobenzoyl) oxime **4g** in $CDCl_3$.

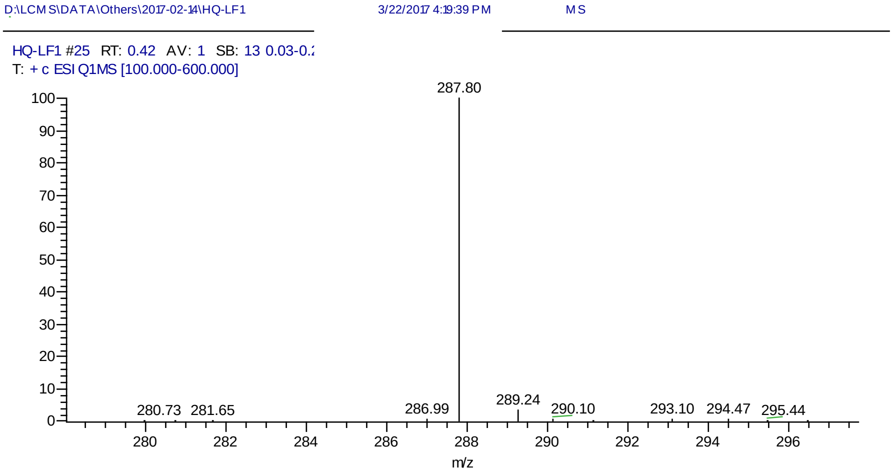

Figure S82. ESI-MS spectrum of (*E*)-verbenone O-(2′-fluorobenzoyl) oxime **4g**.

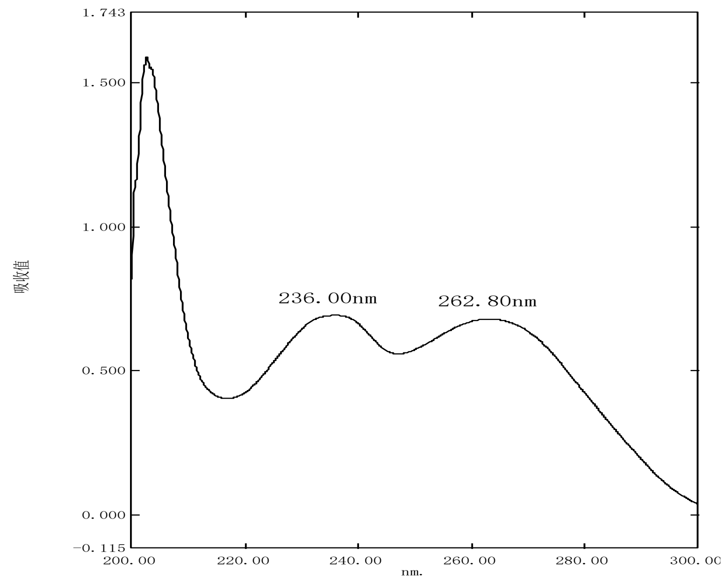

Figure S93. UV-vis spectrum of (*Z*)-verbenone O-(2′-methylbenzoyl) oxime **4h** in EtOH.

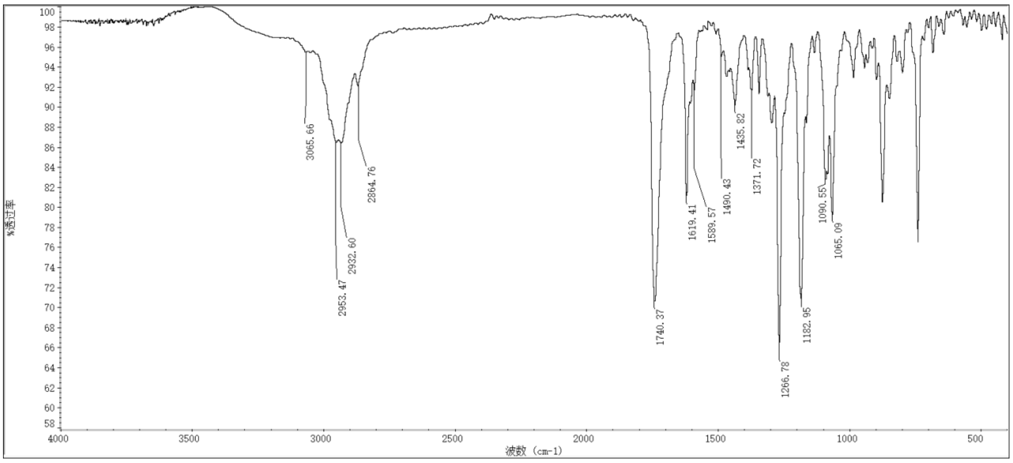

Figure S94. FTIR spectrum of (*Z*)-verbenone O-(3′-methylbenzoyl) oxime **4h**.

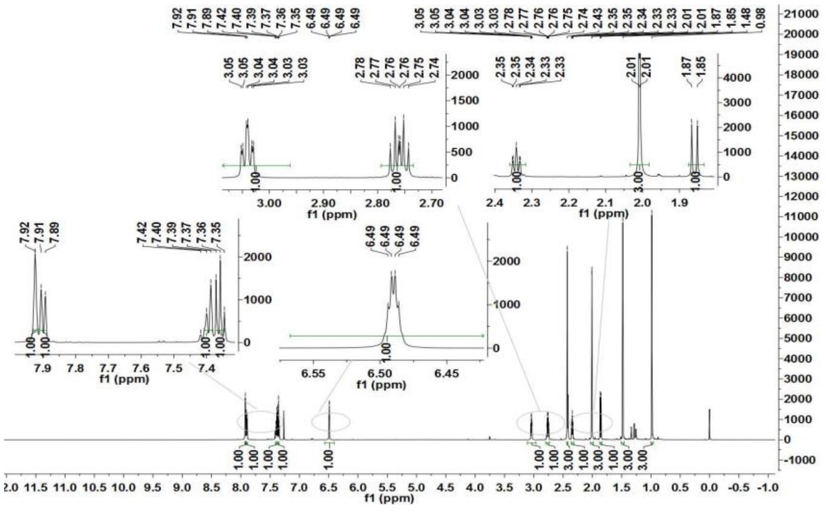

Figure S95. ^{1}H-NMR spectrum of (*Z*)-verbenone O-(3′-methylbenzoyl) oxime **4h** in $CDCl_3$.

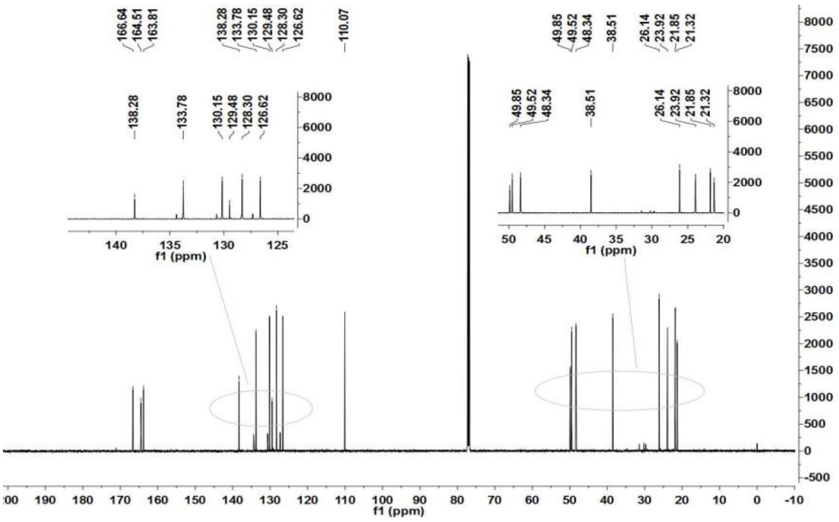

Figure S96. ^{13}C-NMR spectrum of (*Z*)-verbenone O-(3′-methylbenzoyl) oxime **4h** in $CDCl_3$.

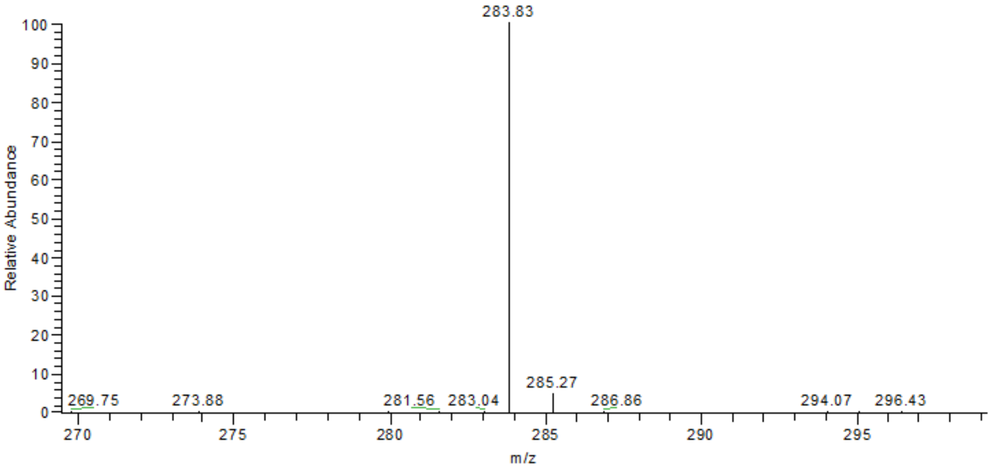

Figure S97. ESI-MS spectrum of (*Z*)-verbenone O-(3′-methylbenzoyl) oxime **4h**.

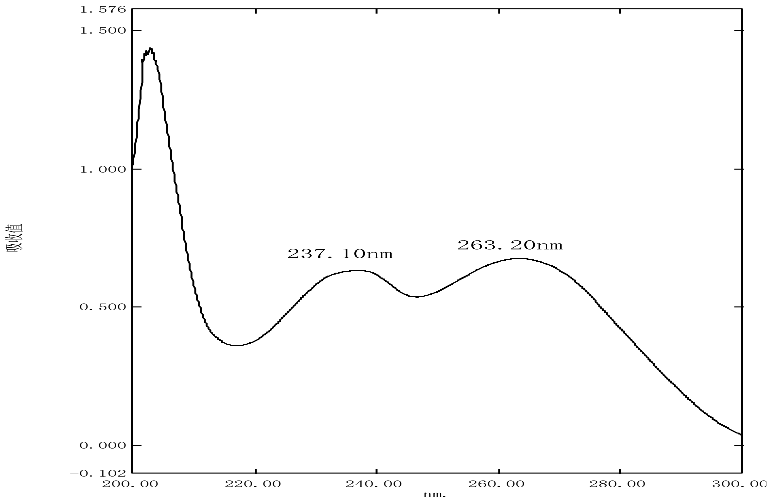

Figure S88. UV-vis spectrum of (*E*)-verbenone O-(3′-methylbenzoyl) oxime **4h** in EtOH.

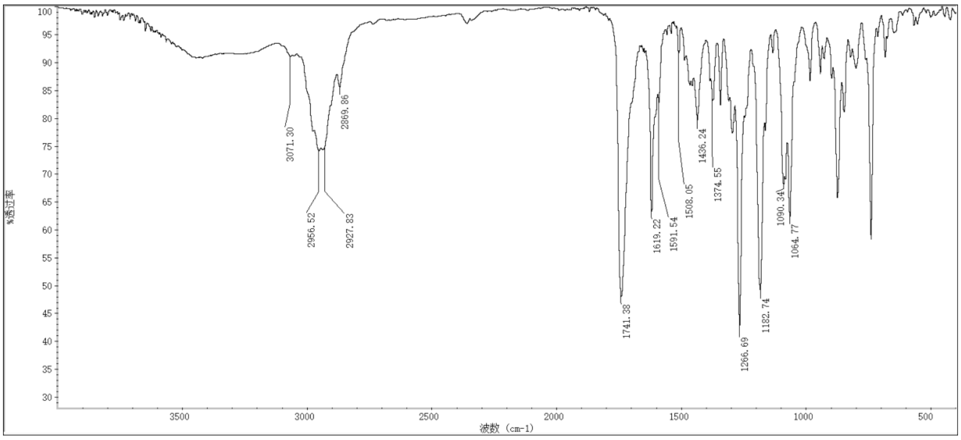

Figure S89. FTIR spectrum of (*E*)-verbenone O-(3′-methylbenzoyl) oxime **4h**.

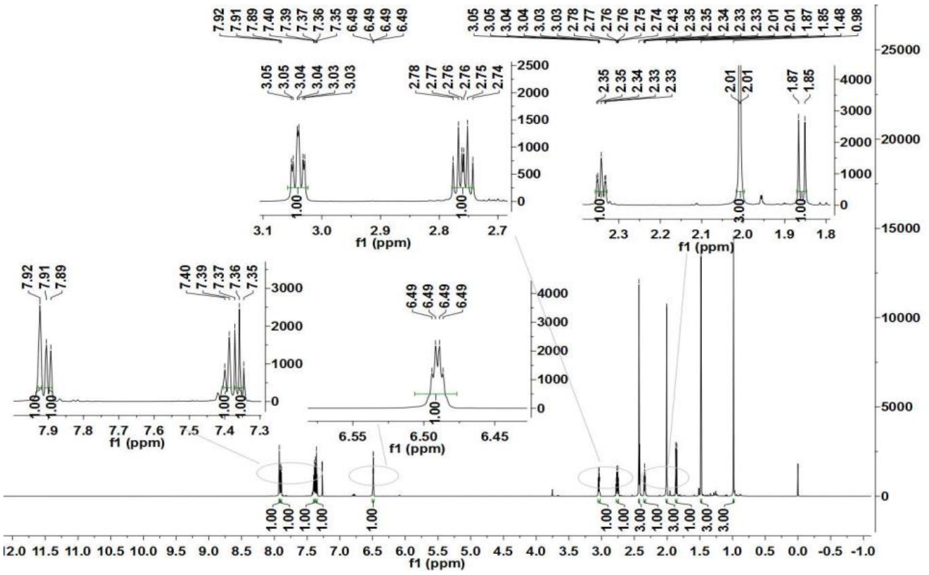

Figure S90. ^{1}H-NMR spectrum of (*E*)-verbenone O-(3′-methylbenzoyl) oxime **4h** in $CDCl_3$.

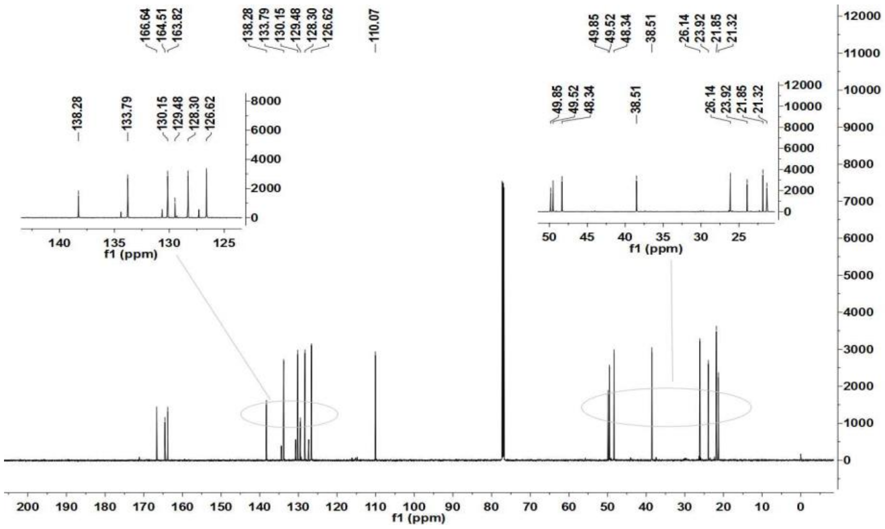

Figure S91. ^{13}C-NMR spectrum of (*E*)-verbenone O-(3′-methylbenzoyl) oxime **4h** in $CDCl_3$.

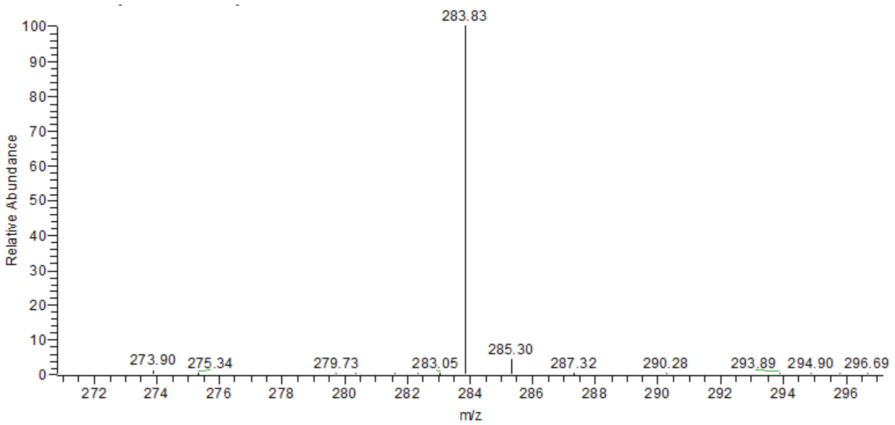

Figure S92. ESI-MS spectrum of (*E*)-verbenone O-(3′-methylbenzoyl) oxime **4h**.

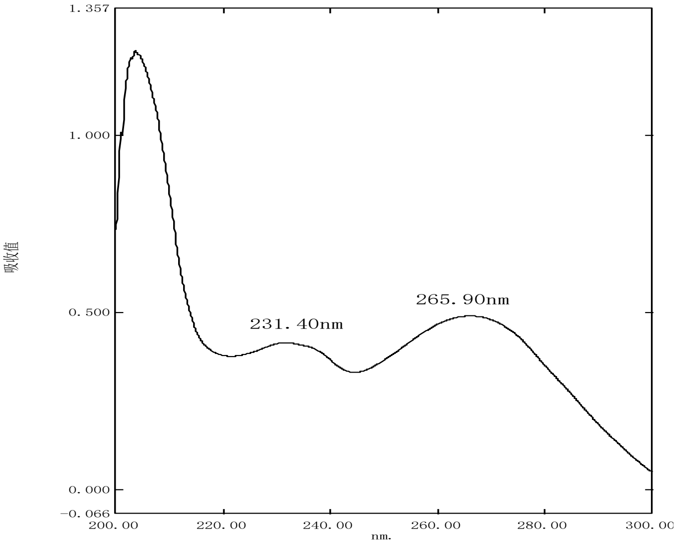

Figure S103. UV-vis spectrum of (*Z*)-verbenone O-(3′-chlorobenzoyl) oxime **4i** in EtOH.

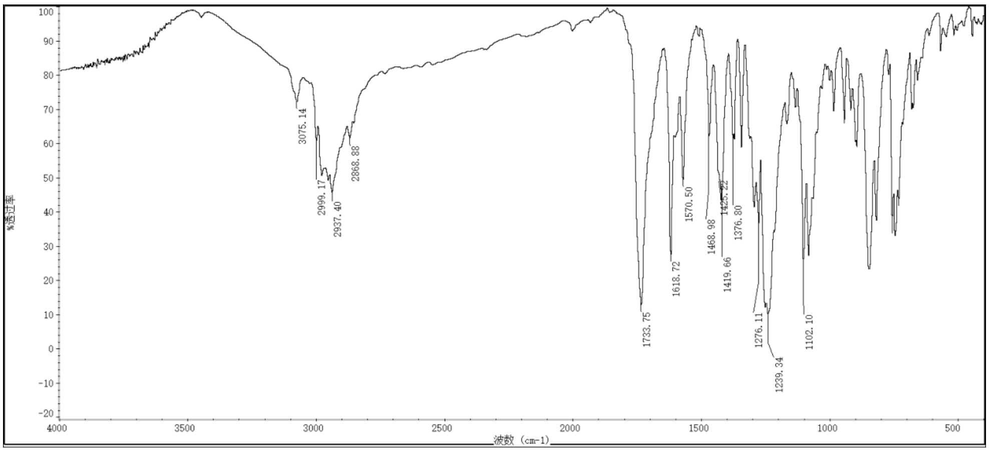

Figure S104. FTIR spectrum of (*Z*)-verbenone O-(3′- chlorobenzoyl) oxime **4i**.

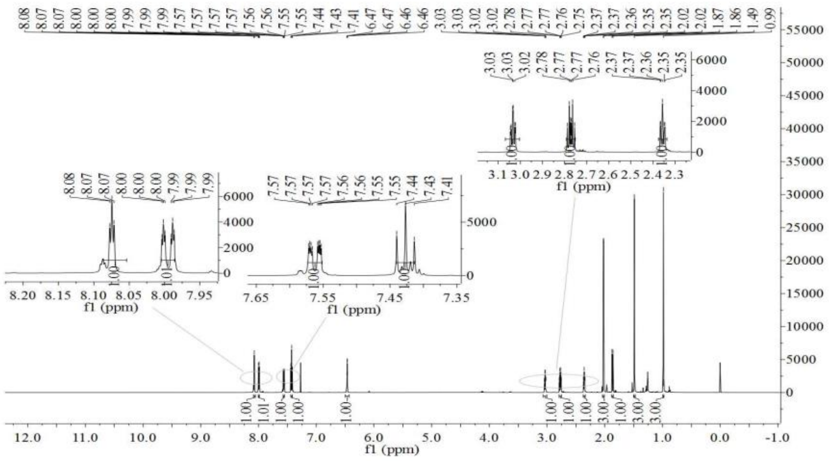

Figure S105. ^{1}H-NMR spectrum of (*Z*)-verbenone O-(3′- chlorobenzoyl) oxime **4i** in $CDCl_3$.

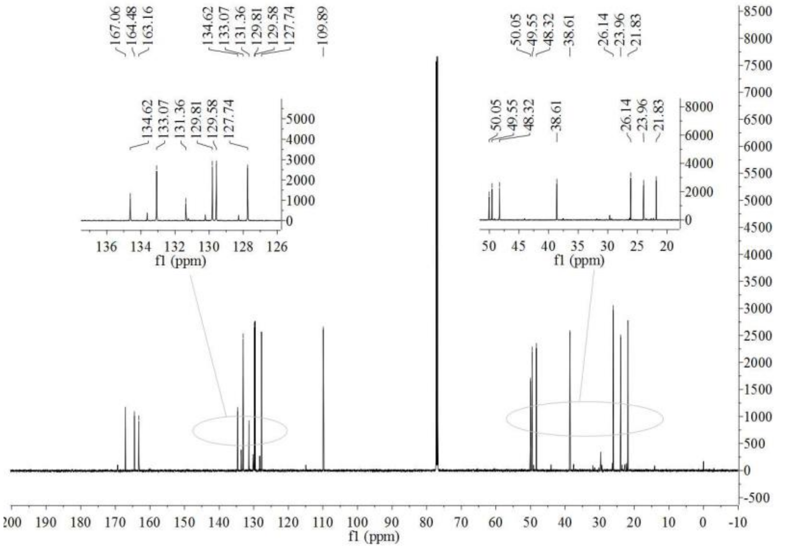

Figure S106. ^{13}C-NMR spectrum of (*Z*)-verbenone O-(3′- chlorobenzoyl) oxime **4i** in $CDCl_3$.

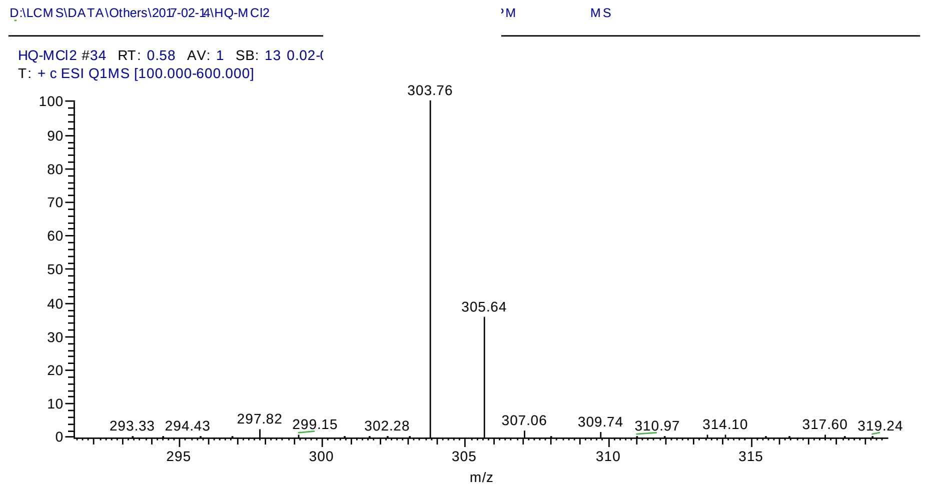

Figure S107. ESI-MS spectrum of (*Z*)-verbenone O-(3′- chlorobenzoyl) oxime **4i**.

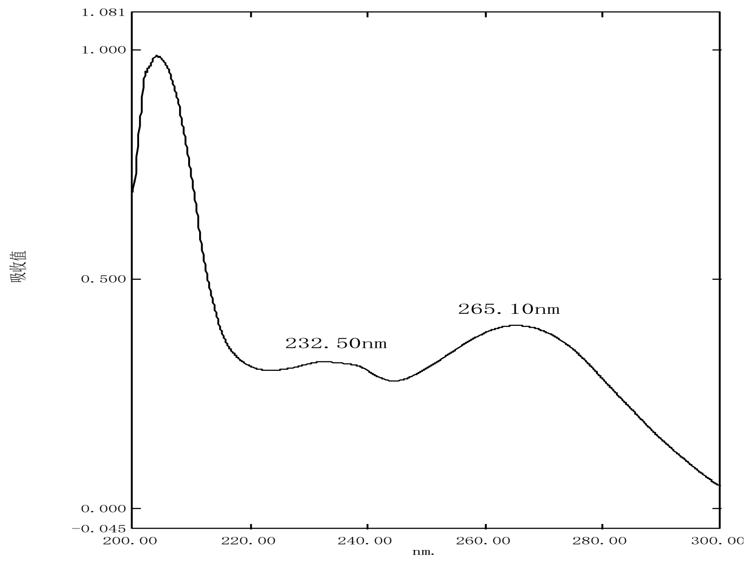

Figure S98. UV-vis spectrum of (*E*)-verbenone O-(3′- chlorobenzoyl) oxime **4i** in EtOH.

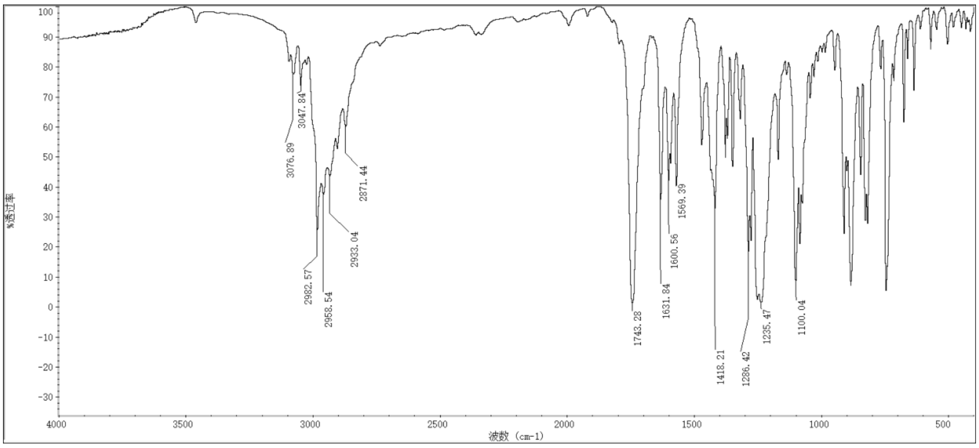

Figure S99. FTIR spectrum of (*E*)-verbenone O-(3′- chlorobenzoyl) oxime **4i**.

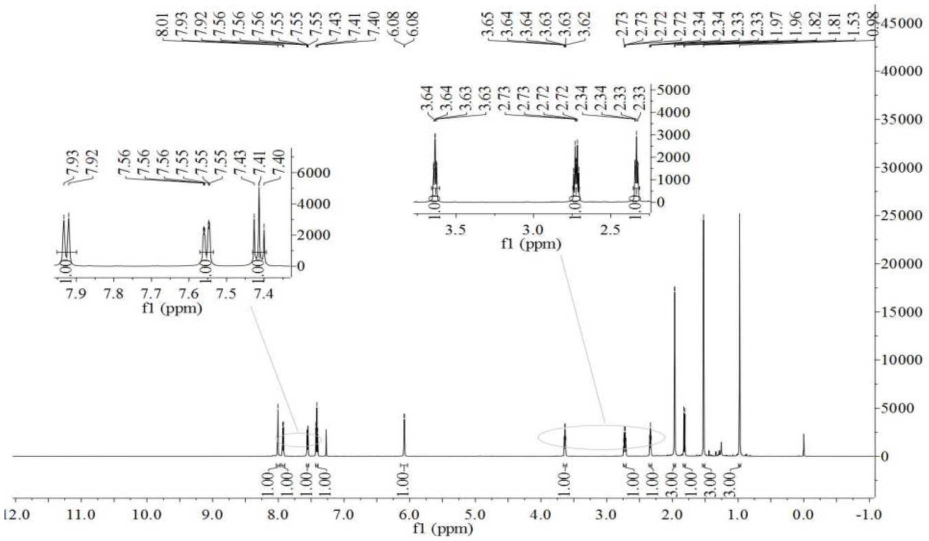

Figure S100. ^{1}H-NMR spectrum of (*E*)-verbenone O-(3′- chlorobenzoyl) oxime **4i** in $CDCl_3$.

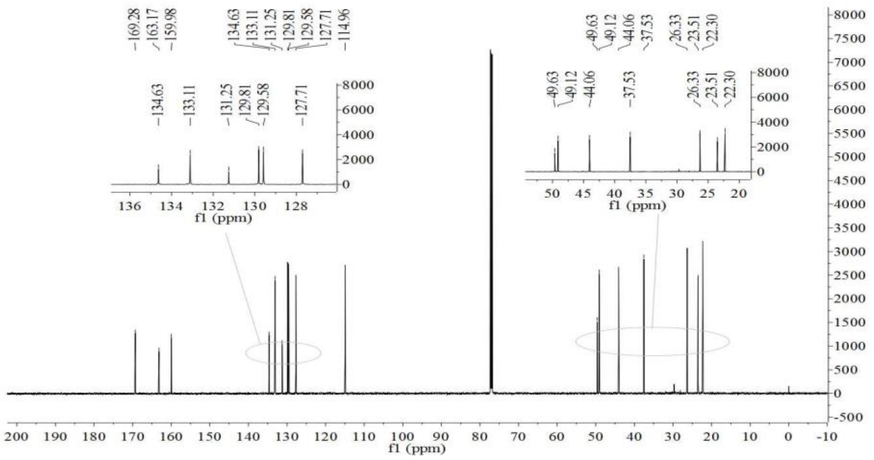

Figure S101. ^{13}C-NMR spectrum of (*E*)-verbenone O-(3′- chlorobenzoyl) oxime **4i** in $CDCl_3$.

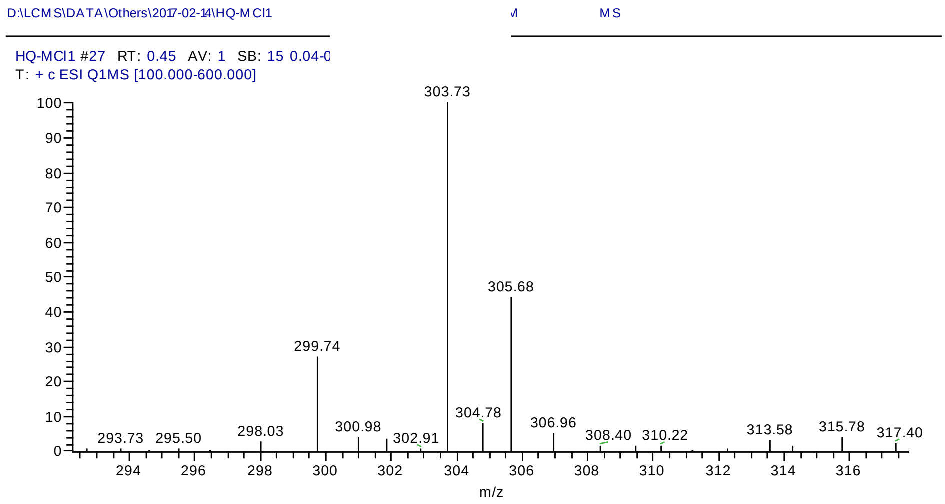

Figure S102. ESI-MS spectrum of (*E*)-verbenone O-(3′- chlorobenzoyl) oxime **4i**.

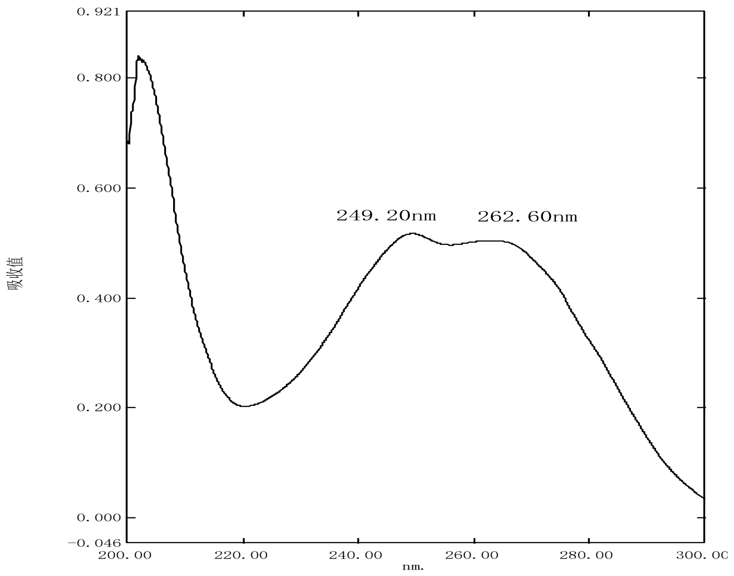

Figure S108. UV-vis spectrum of (*Z*)-verbenone O-(4′-bromobenzoyl) oxime **4j** in EtOH.

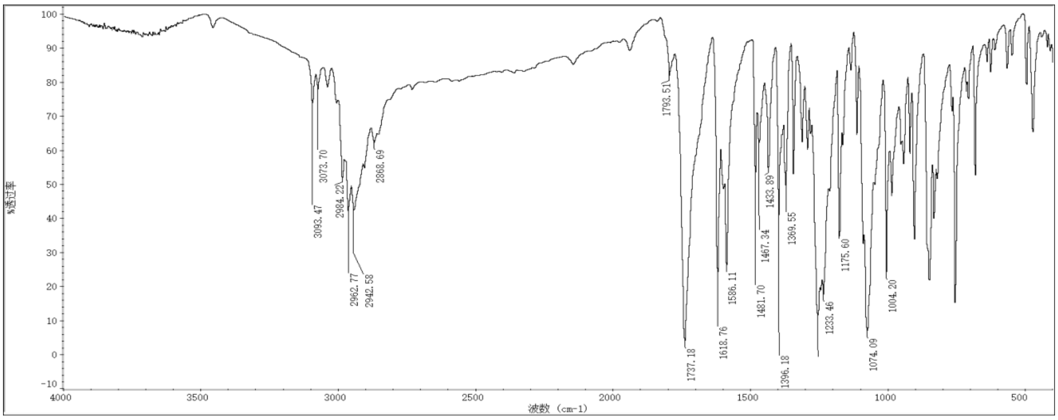

Figure S109. FTIR spectrum of (*Z*)-verbenone O-(4′-bromobenzoyl) oxime **4j**.

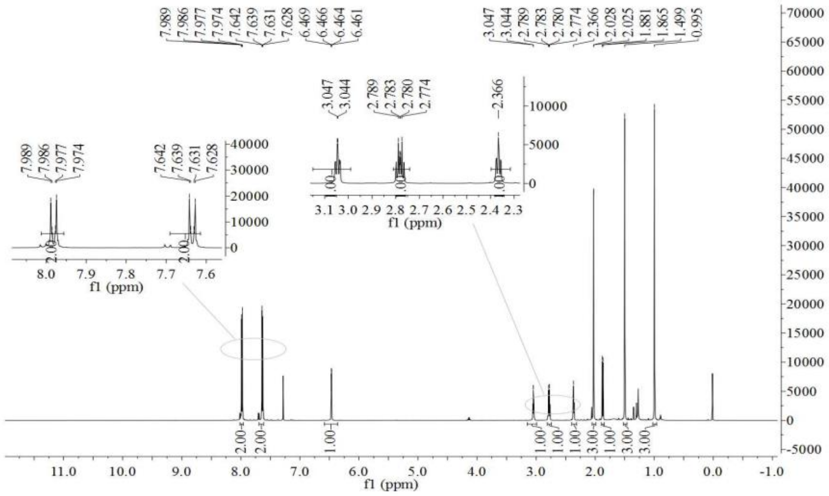

Figure S110. 1H-NMR spectrum of (*Z*)-verbenone O-(4′-bromobenzoyl) oxime **4j** in $CDCl_3$.

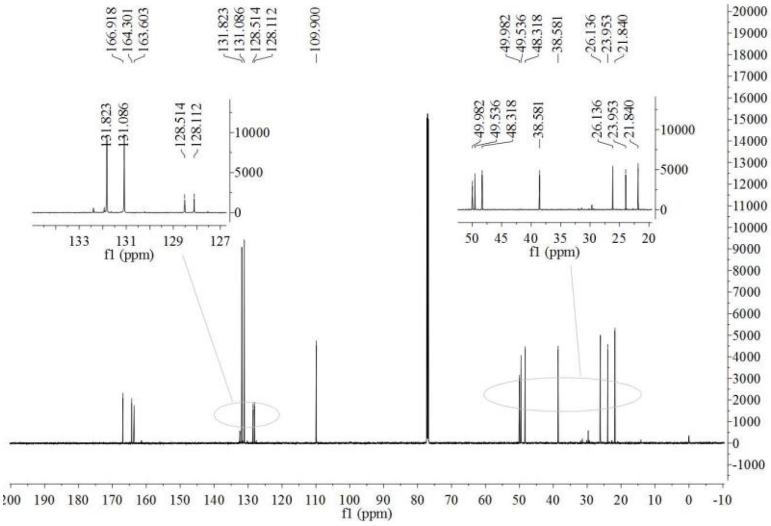

Figure S111. 13C-NMR spectrum of (*Z*)-verbenone O-(4′-bromobenzoyl) oxime **4j** in $CDCl_3$.

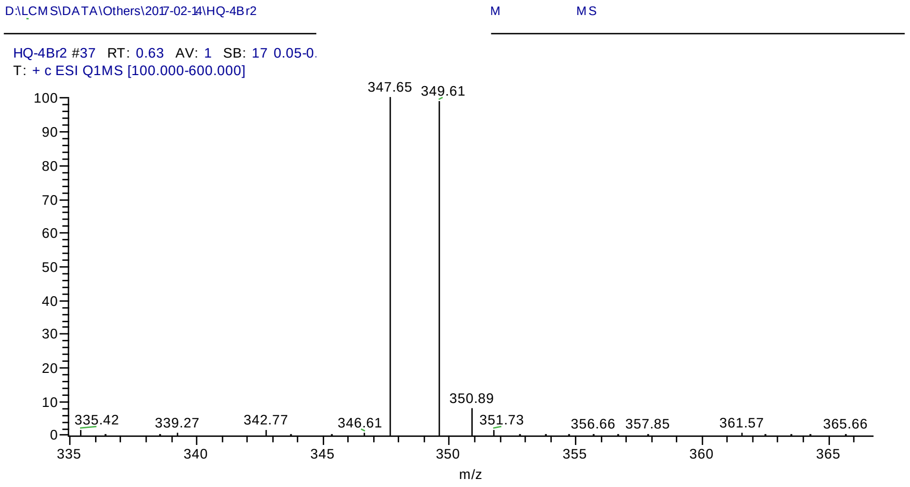

Figure S112. ESI-MS spectrum of (*Z*)-verbenone O-(4′-bromobenzoyl) oxime **4j**.

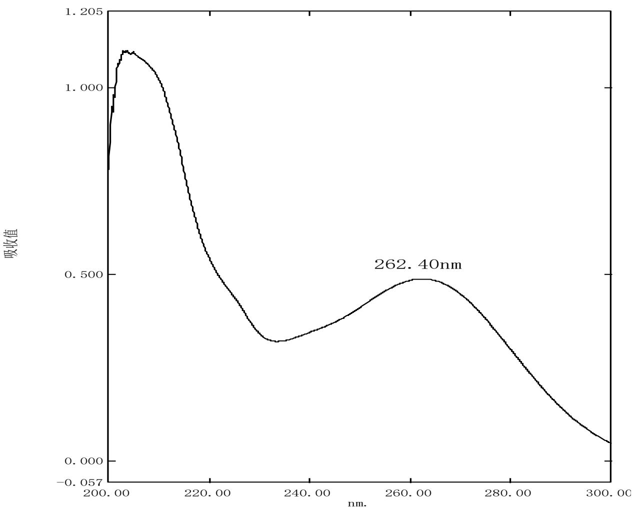

Figure S118. UV-vis spectrum of (*Z*)-verbenone O-(2′, 3′-dichlorobenzoyl) oxime **4k** in EtOH.

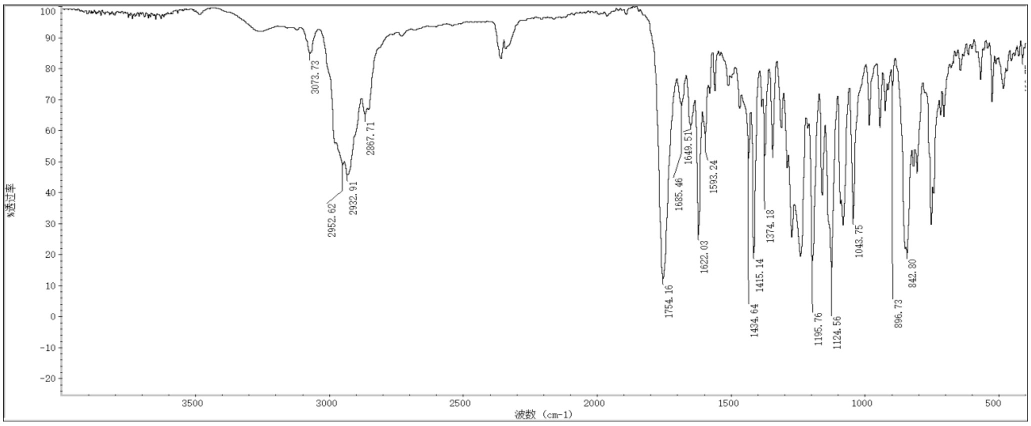

Figure S119. FTIR spectrum of (*Z*)-verbenone O-(2′, 3′-dichlorobenzoyl) oxime **4k**.

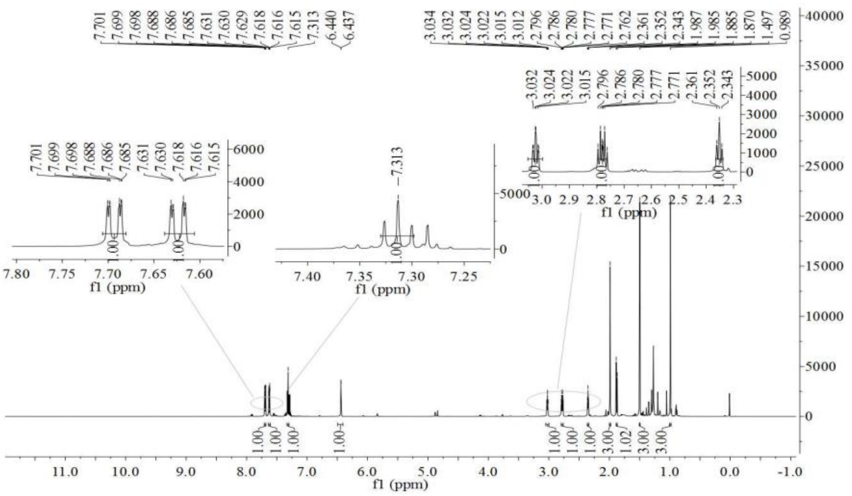

Figure S120. ^{1}H-NMR spectrum of (*Z*)-verbenone O-(2′, 3′-dichlorobenzoyl) oxime **4k** in $CDCl_3$.

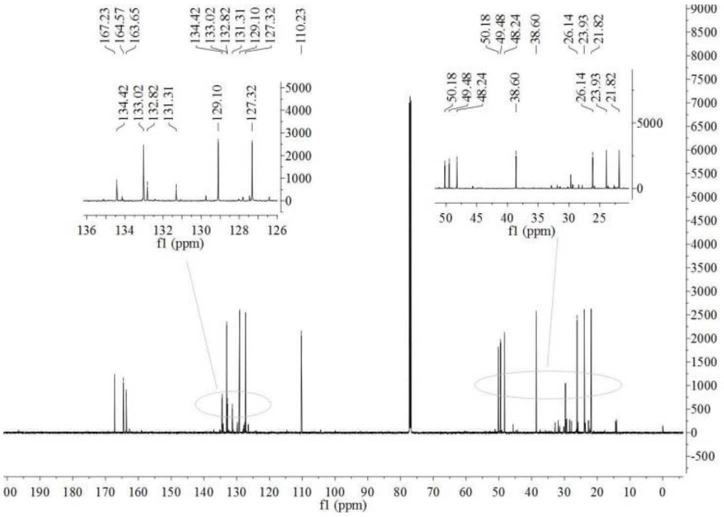

Figure S121. ^{13}C-NMR spectrum of (*Z*)-verbenone O-(2′, 3′-dichlorobenzoyl) oxime **4k** in $CDCl_3$.

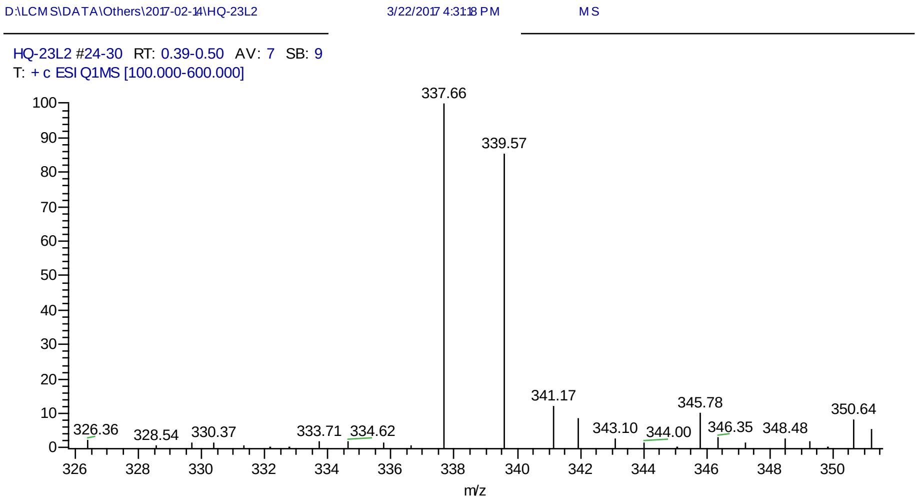

Figure S122. ESI-MS spectrum of (*Z*)-verbenone O-(2′, 3′-dichlorobenzoyl) oxime **4k**.

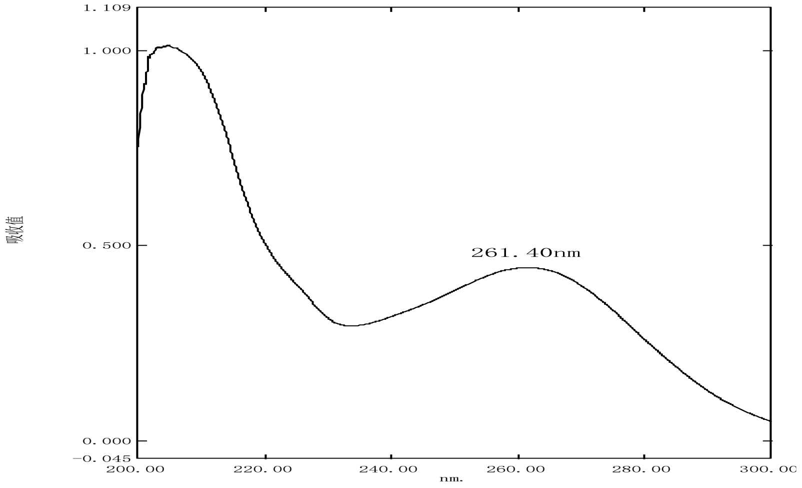

Figure S113. UV-vis spectrum of (*E*)-verbenone O-(2′, 3′-dichlorobenzoyl) oxime **4k** in EtOH.

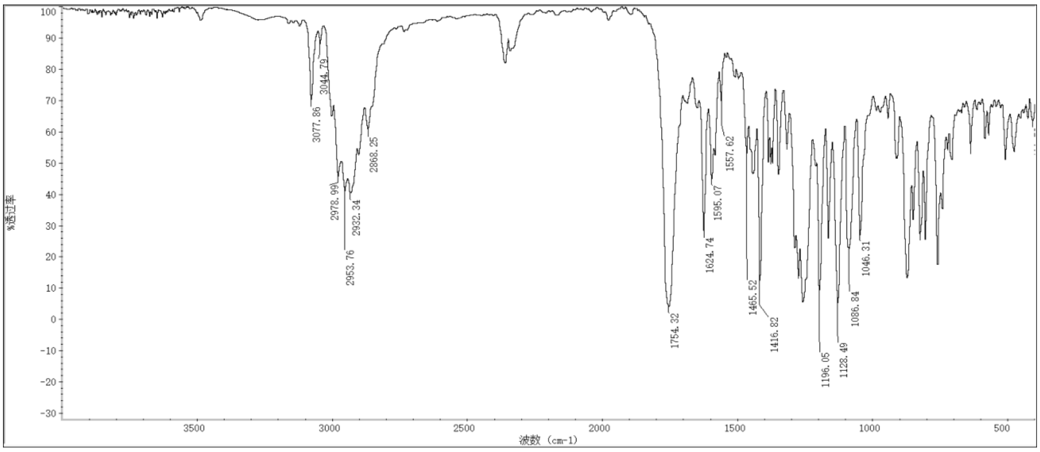

Figure S114. FTIR spectrum of (*E*)-verbenone O-(2′, 3′-dichlorobenzoyl) oxime **4k**.

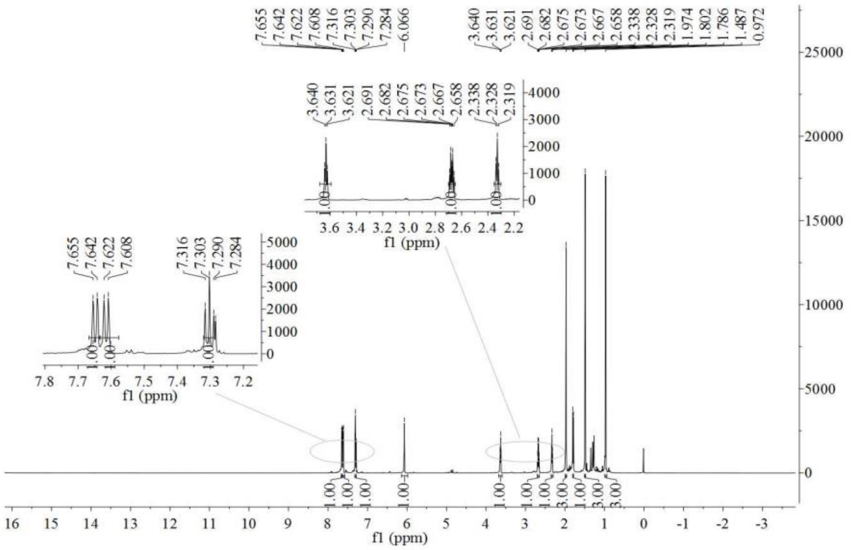

Figure S115. ^{1}H-NMR spectrum of (*E*)-verbenone O-(2′, 3′-dichlorobenzoyl) oxime **4k** in $CDCl_3$.

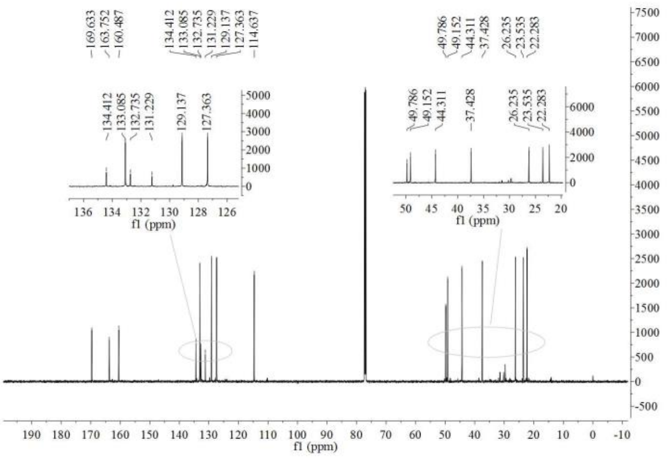

Figure S116. ^{13}C-NMR spectrum of (*E*)-verbenone O-(2′, 3′-dichlorobenzoyl) oxime **4k** in $CDCl_3$.

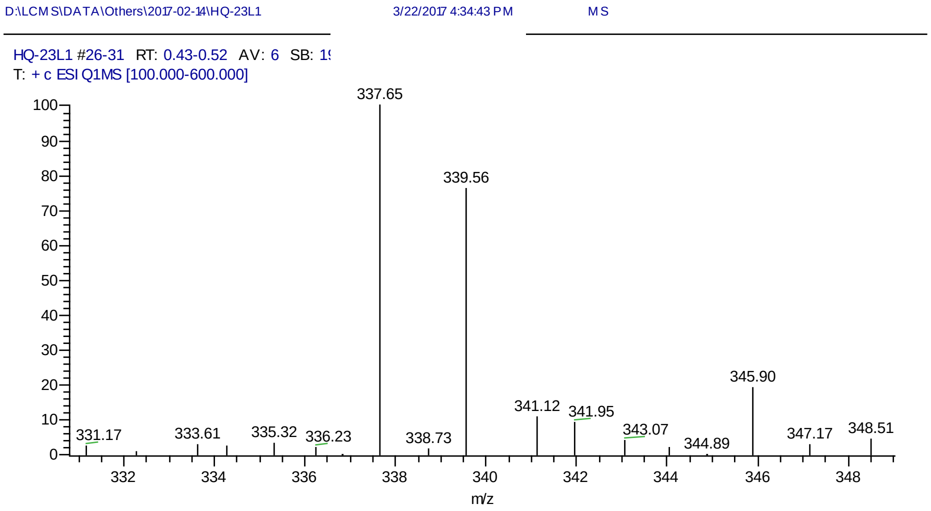

Figure S117. ESI-MS spectrum of (*E*)-verbenone O-(2′, 3′-dichlorobenzoyl) oxime **4k**.

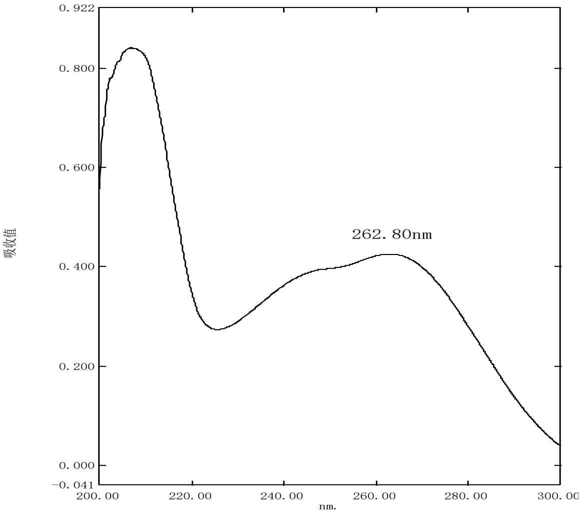

Figure S128. UV-vis spectrum of (*Z*)-verbenone O-(2′, 4′-dichlorobenzoyl) oxime **4l** in EtOH.

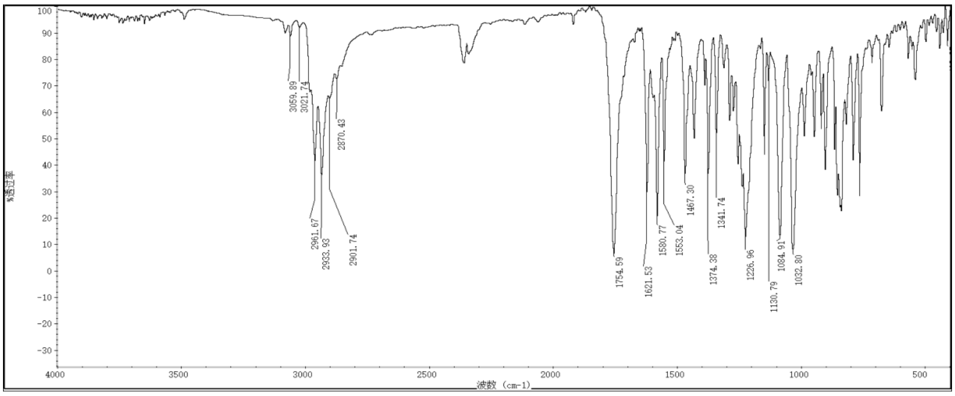

Figure S129. FTIR spectrum of (*Z*)-verbenone O-(2′, 4′-dichlorobenzoyl) oxime **4l**.

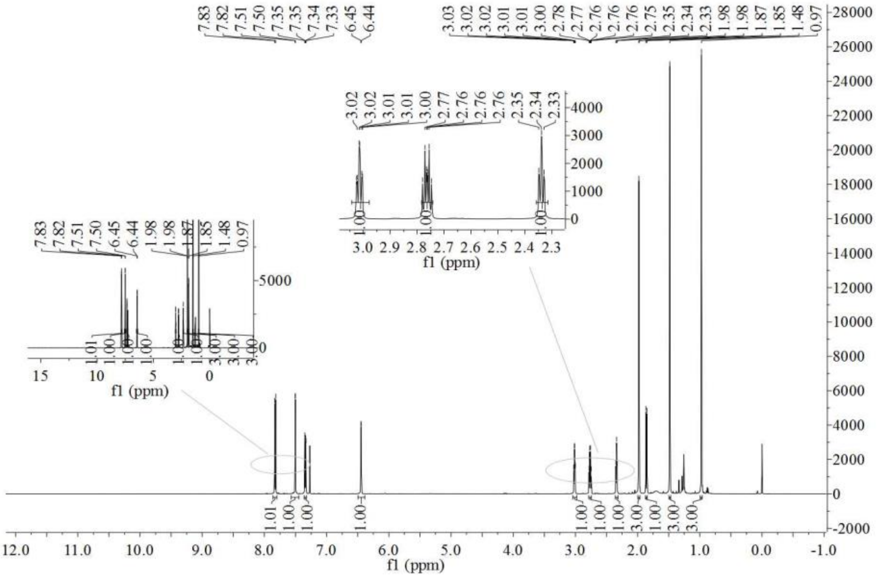

Figure S130. ^{1}H-NMR spectrum of (*Z*)-verbenone O-(2′, 4′-dichlorobenzoyl) oxime **4l** in $CDCl_3$.

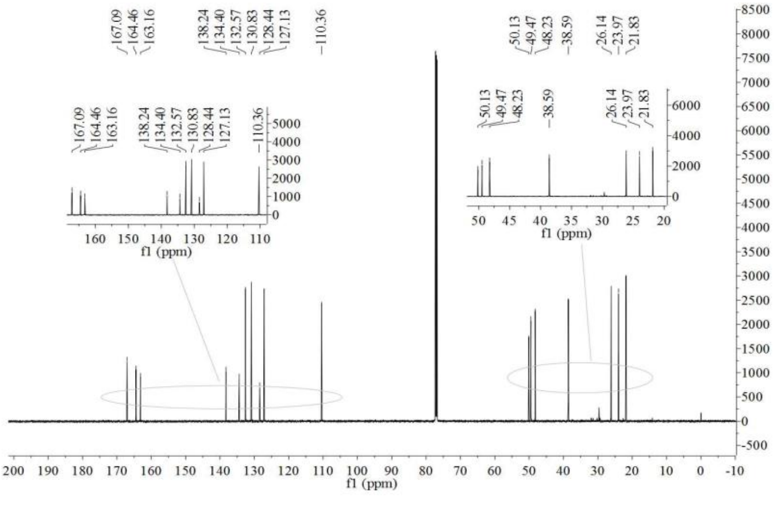

Figure S131. ^{13}C-NMR spectrum of (*Z*)-verbenone O-(2′, 4′-dichlorobenzoyl) oxime **4l** in $CDCl_3$.

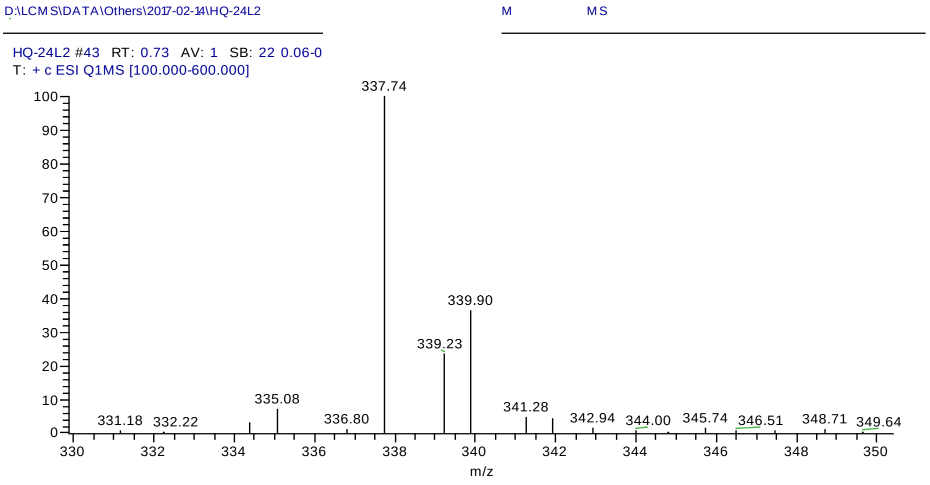

Figure S132. ESI-MS spectrum of (*Z*)-verbenone O-(2′, 4′-dichlorobenzoyl) oxime **4l**.

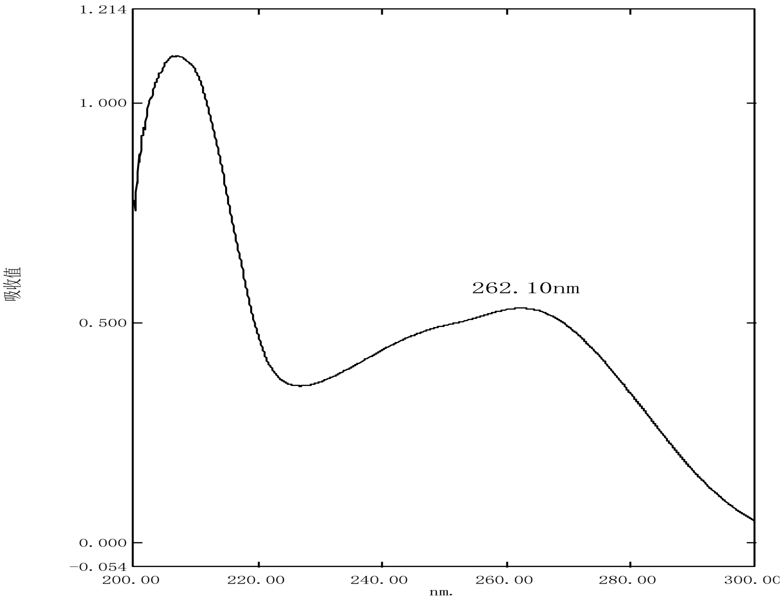

Figure S123. UV-vis spectrum of (*E*)-verbenone O-(2′, 4′-dichlorobenzoyl) oxime **4l** in EtOH.

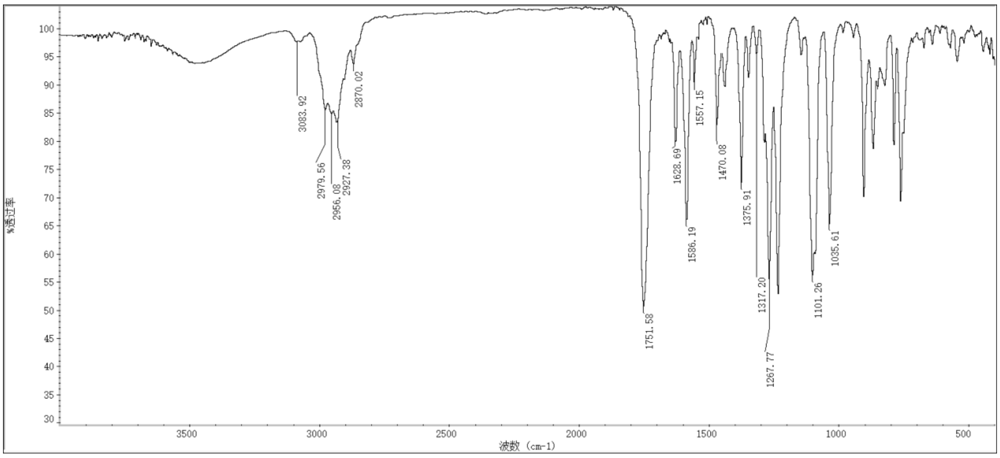

Figure S124. FTIR spectrum of (*E*)-verbenone O-(2′, 4′-dichlorobenzoyl) oxime **4l**.

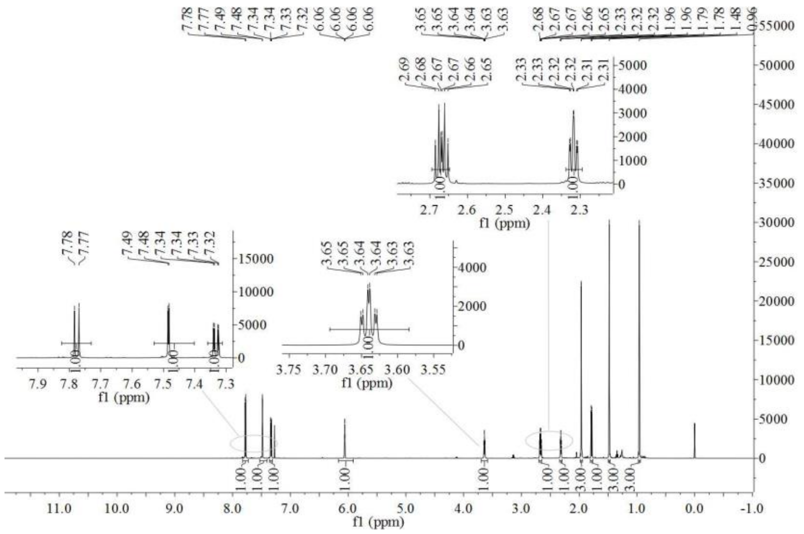

Figure S125. ^{1}H-NMR spectrum of (*E*)-verbenone O-(2′, 4′-dichlorobenzoyl) oxime **4l** in $CDCl_3$.

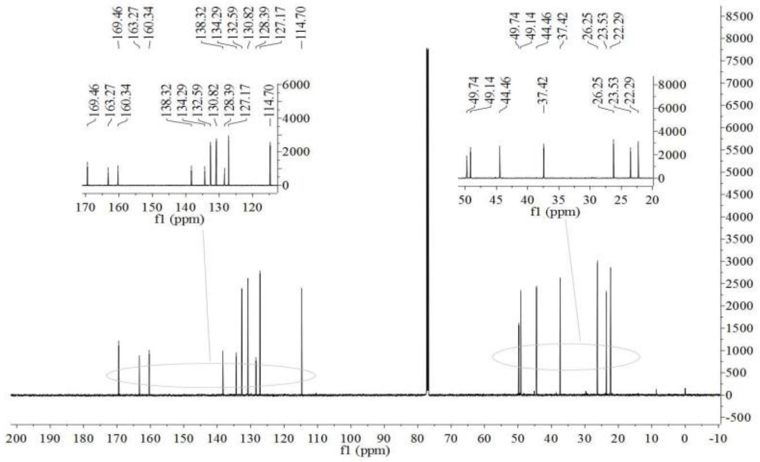

Figure S126. ^{13}C-NMR spectrum of (*E*)-verbenone O-(2′, 4′-dichlorobenzoyl) oxime **4l** in $CDCl_3$.

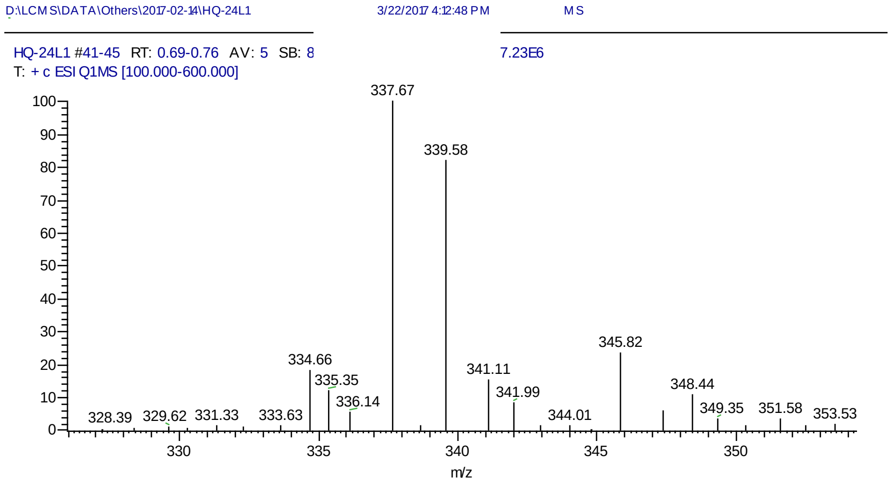

Figure S127. ESI-MS spectrum of (*E*)-verbenone O-(2′, 4′-dichlorobenzoyl) oxime **4l**.

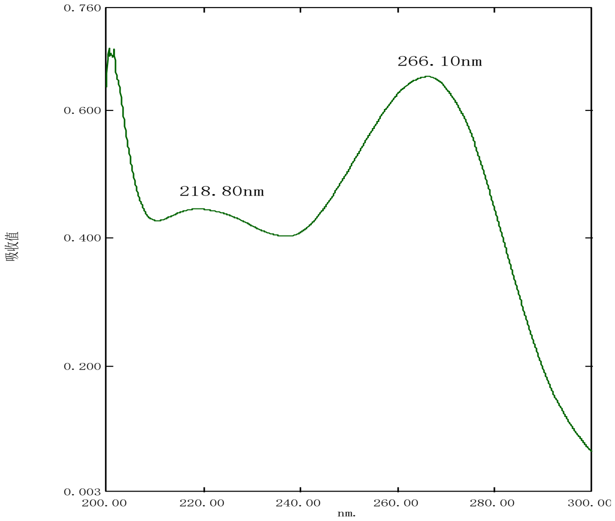

Figure S138. UV-vis spectrum of (*Z*)-verbenone O-2-chloropyridylcarbonyl oxime **4m** in EtOH.

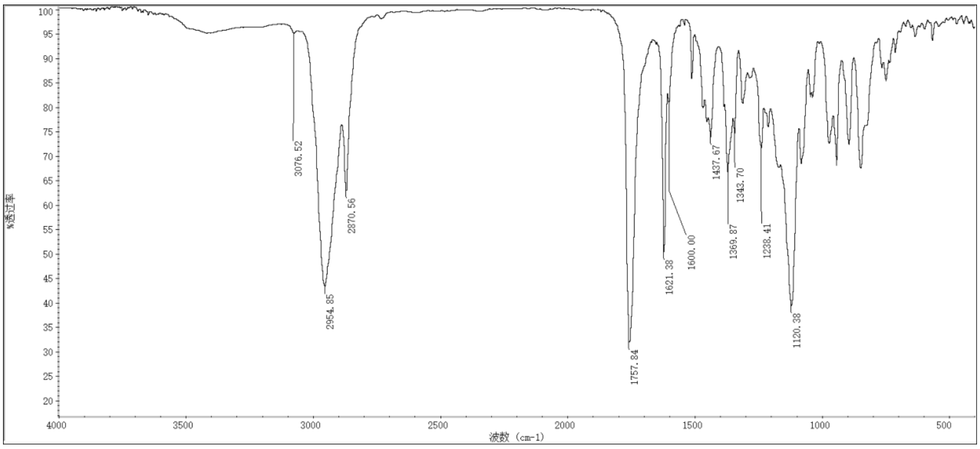

Figure S139. FTIR spectrum of (*Z*)-verbenone O-2-chloropyridylcarbonyl oxime **4m**.

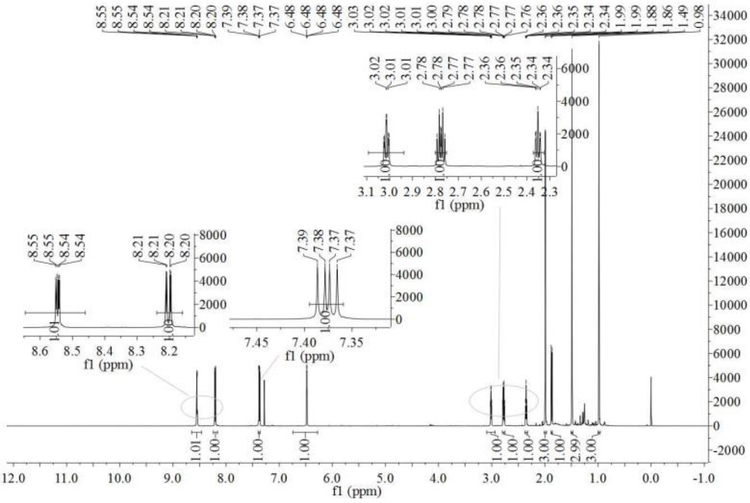

Figure S140. ^{1}H-NMR spectrum of (*Z*)-verbenone O-2-chloropyridylcarbonyl oxime **4m** in $CDCl_3$.

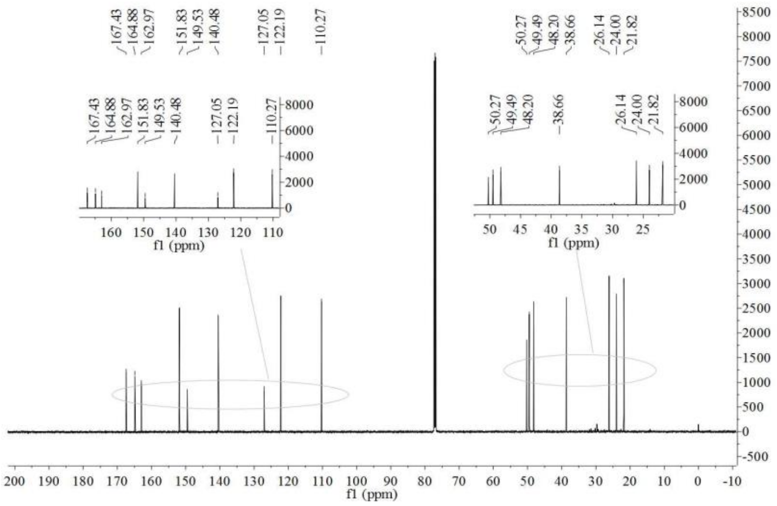

Figure S141. ^{13}C-NMR spectrum of (*Z*)-verbenone O-2-chloropyridylcarbonyl oxime **4m** in $CDCl_3$.

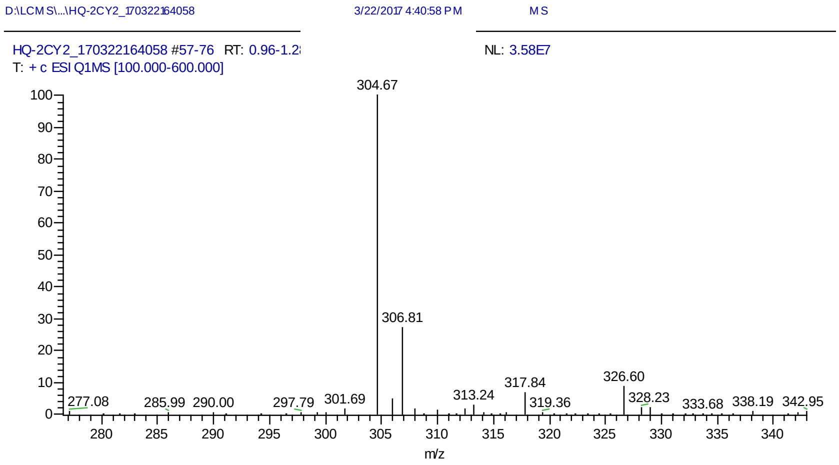

Figure S142. ESI-MS spectrum of (*Z*)-verbenone O-2-chloropyridylcarbonyl oxime **4m**

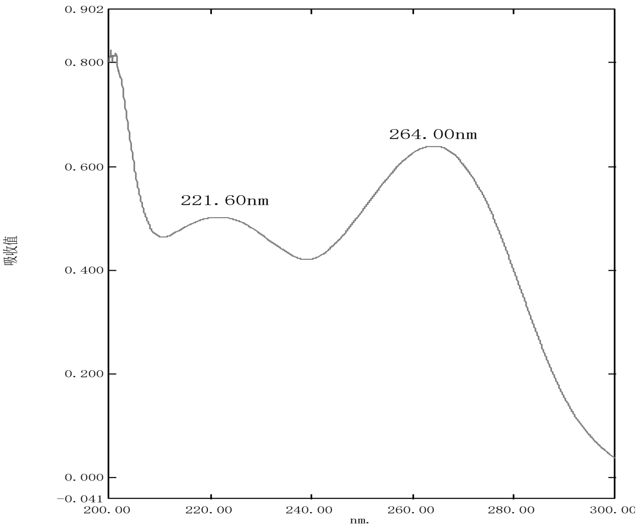

Figure S133. UV-vis spectrum of (*E*)-verbenone O-2-chloropyridylcarbonyl oxime **4m** in EtOH.

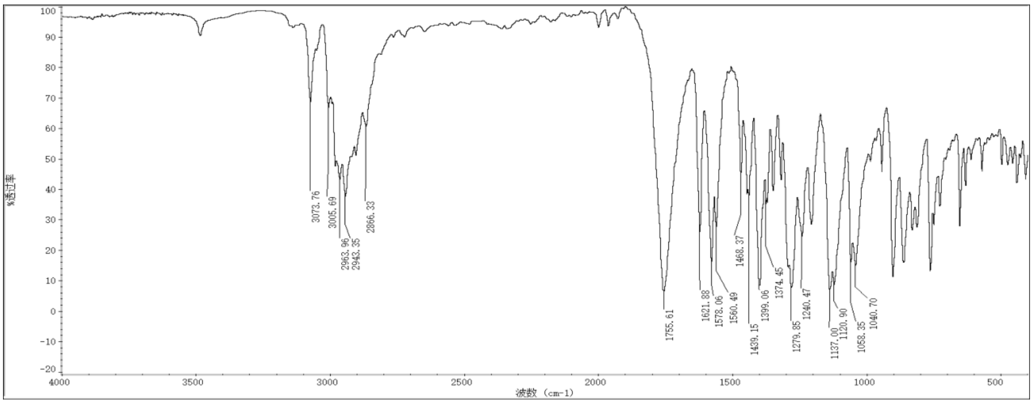

Figure S134. FTIR spectrum of (*E*)-verbenone O-2-chloropyridylcarbonyl oxime **4m**.

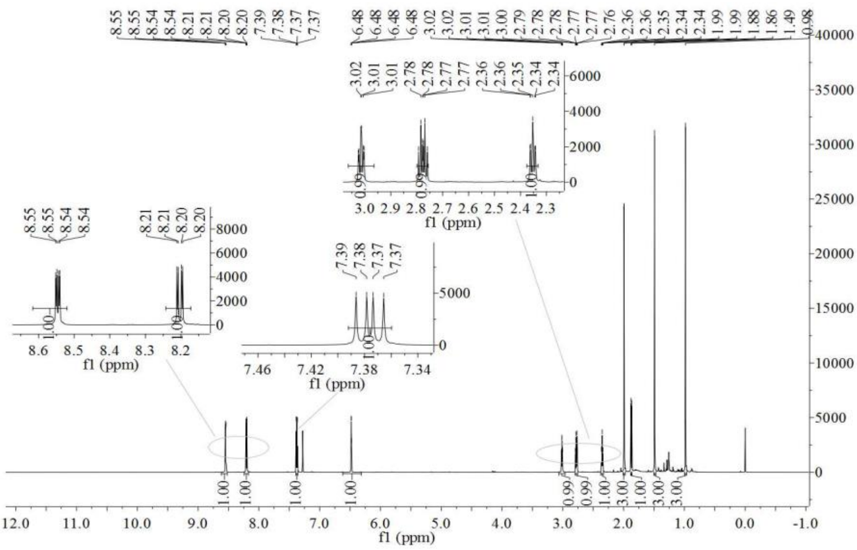

Figure S135. ^{1}H-NMR spectrum of (*E*)-verbenone O-2-chloropyridylcarbonyl oxime **4m** in $CDCl_3$.

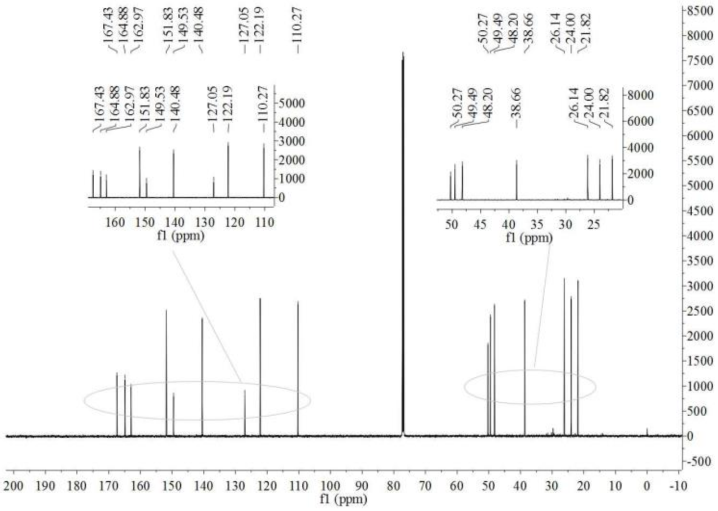

Figure S136. ^{13}C-NMR spectrum of (*E*)-verbenone O-2-chloropyridylcarbonyl oxime **4m** in $CDCl_3$.

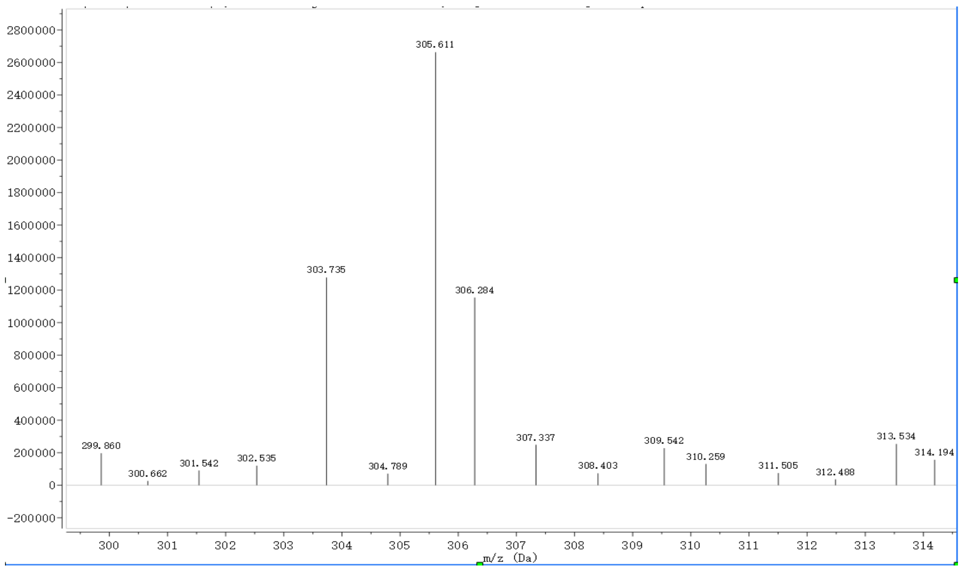

Figure S137. ESI-MS spectrum of (*E*)-verbenone O-2-chloropyridylcarbonyl oxime **4m**.

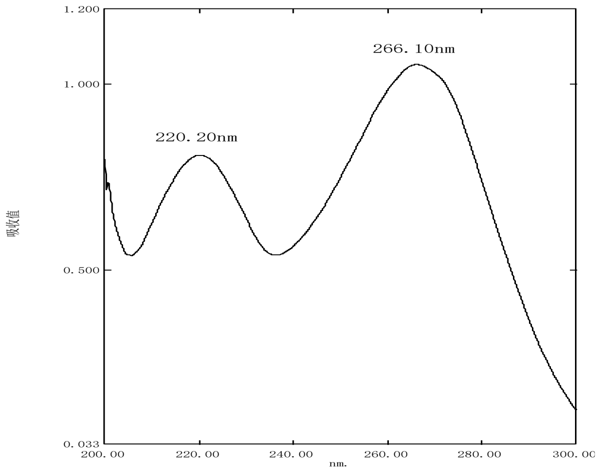

Figure S148. UV-vis spectrum of (*Z*)-verbenone O-*β*-pyridylcarbonyl oxime **4n** in EtOH.

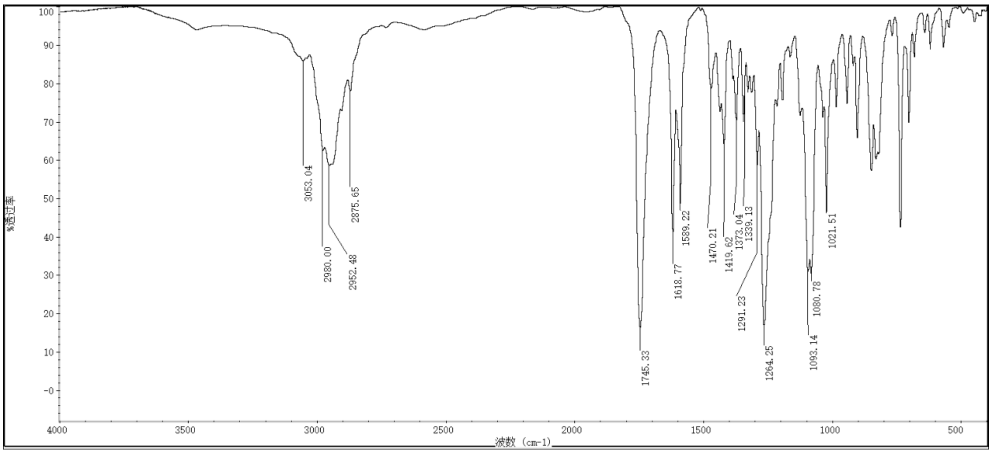

Figure S149. FTIR spectrum of (*Z*)-verbenone O-*β*-pyridylcarbonyl oxime **4n**.

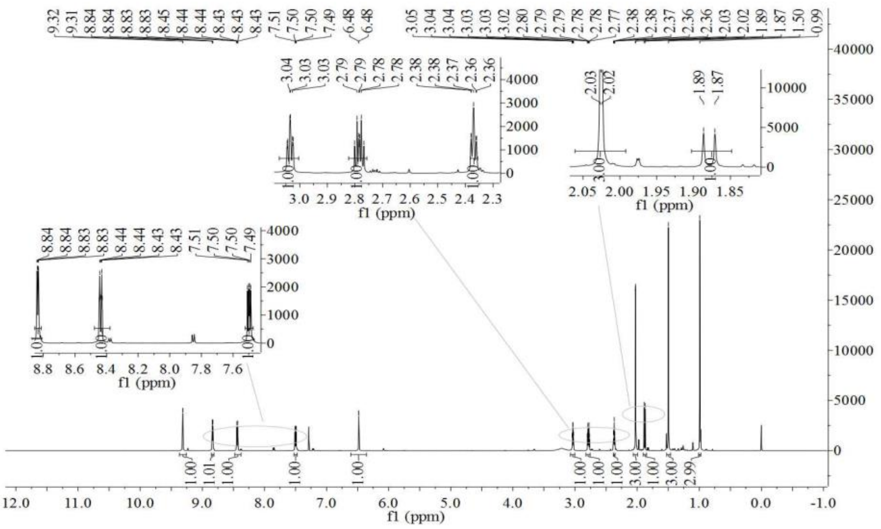

Figure S150. ^{1}H-NMR spectrum of (*Z*)-verbenone O-*β*-pyridylcarbonyl oxime **4n** in $CDCl_3$.

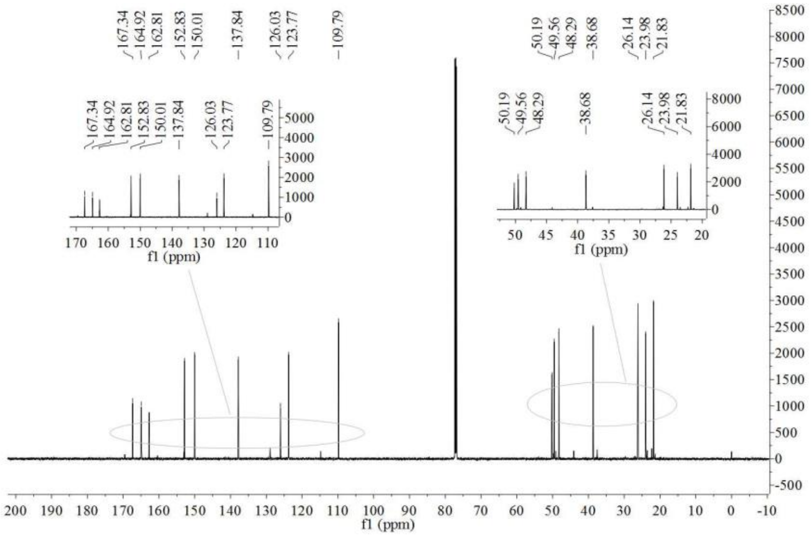

Figure S151. ^{13}C-NMR spectrum of (*Z*)-verbenone O-*β*-pyridylcarbonyl oxime **4n** in $CDCl_3$.

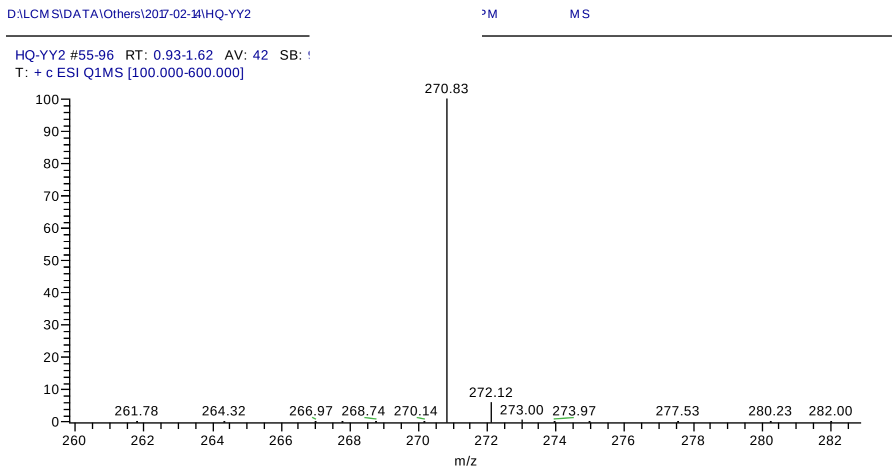

Figure S152. ESI-MS spectrum of (*Z*)-verbenone O-*β*-pyridylcarbonyl oxime **4n**.

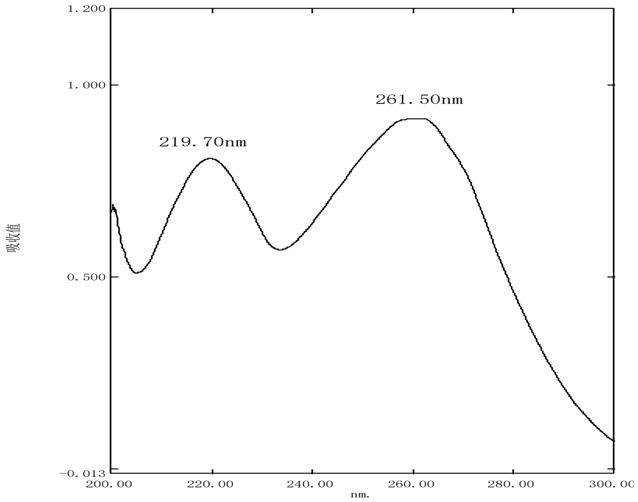

Figure S143. UV-vis spectrum of (*E*)-verbenone O-*β*-pyridylcarbonyl oxime **4n** in EtOH.

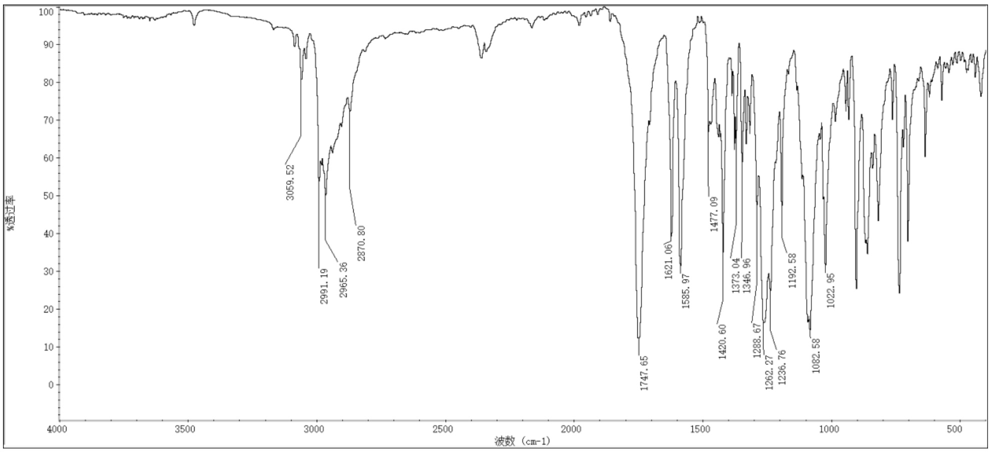

Figure S144. FTIR spectrum of (*E*)-verbenone O-*β*-pyridylcarbonyl oxime **4n**.

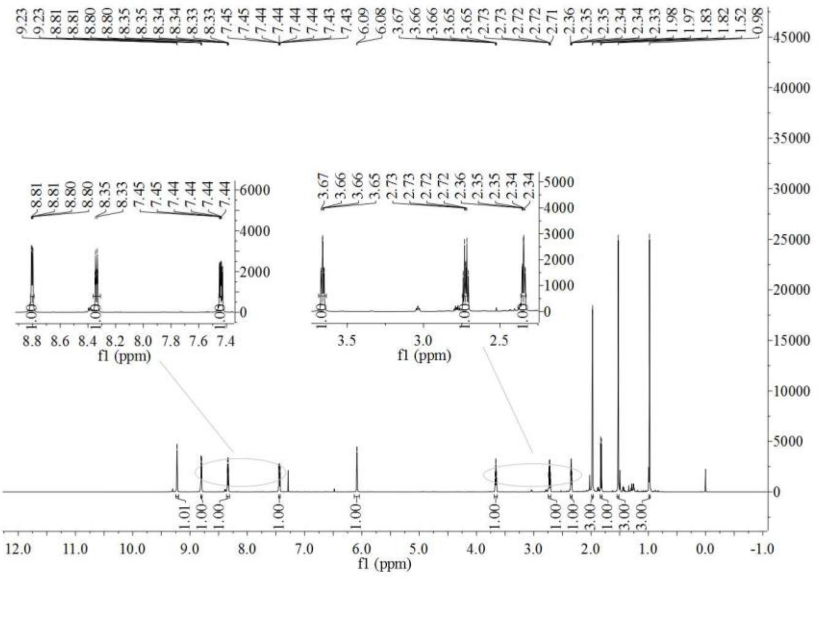

Figure S145. ^{1}H-NMR spectrum of (*E*)-verbenone O-*β*-pyridylcarbonyl oxime **4n** in $CDCl_3$.

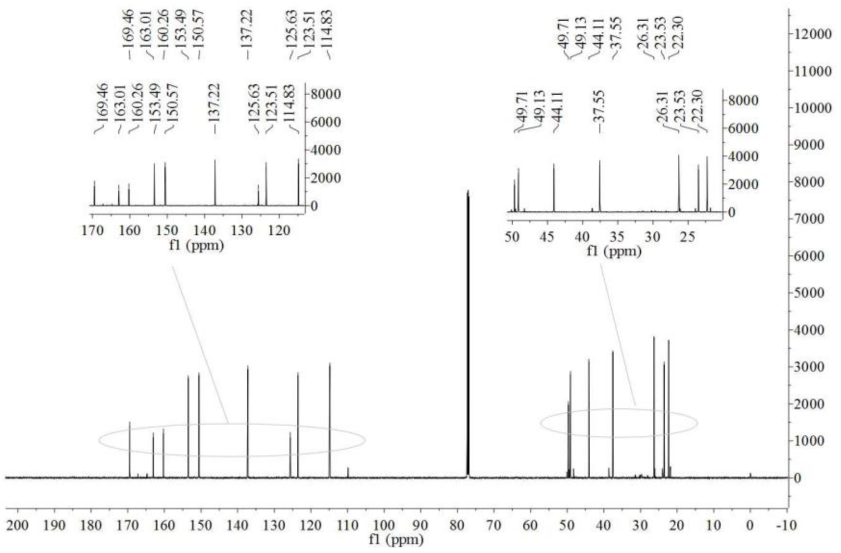

Figure S146. ^{13}C-NMR spectrum of (*E*)-verbenone O-*β*-pyridylcarbonyl oxime **4n** in $CDCl_3$.

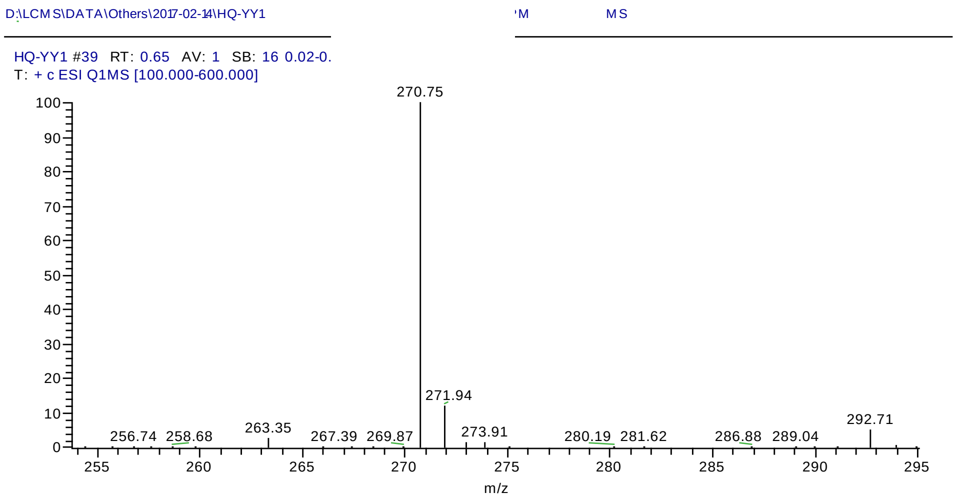

Figure S147. ESI-MS spectrum of (*E*)-verbenone O-*β*-pyridylcarbonyl oxime **4n**.
